# First-in-human, double-blind, randomized phase 1b study of peptide immunotherapy IMCY-0098 in new-onset type 1 diabetes

**DOI:** 10.1186/s12916-023-02900-z

**Published:** 2023-05-24

**Authors:** Jean Van Rampelbergh, Peter Achenbach, Richard David Leslie, Mohammad Alhadj Ali, Colin Dayan, Bart Keymeulen, Katharine R. Owen, Martin Kindermans, Frédéric Parmentier, Vincent Carlier, Roxana R. Ahangarani, Evelien Gebruers, Nicolas Bovy, Luc Vanderelst, Marcelle Van Mechelen, Pierre Vandepapelière, Christian Boitard

**Affiliations:** 1 Imcyse S.A., Avenue Pré-Aily 14, Angleur, 4031 Liège, Belgium; 2 Institute of Diabetes Research, Helmholtz Zentrum München, German Research Center for Environmental Health, Munich-Neuherberg, Germany; 3 Forschergruppe Diabetes, Technical University Munich, Klinikum Rechts Der Isar, Munich, Germany; 4 Department of Immunobiology, Queen Mary University of London, London, UK; 5 Diabetes Research Group, Cardiff University School of Medicine, Cardiff University, Cardiff, UK; 6 Member of Belgian Diabetes Registry, Academic Hospital and Diabetes Research Center, Vrije Universiteit Brussel, Brussels, Belgium; 7 Oxford Centre for Diabetes, Endocrinology and Metabolism, University of Oxford, Oxford, UK; 8 Oxford NIHR Biomedical Research Centre, Churchill Hospital, Oxford, UK; 9 Ariana Pharmaceuticals SA, Paris, France; 10 Inserm U1016, Cochin Institute, Paris, France; 11 Medical Faculty, Université de Paris, Paris, France

**Keywords:** Type 1 diabetes, Immunotherapy, T cells, Beta-cells, Clinical study, Safety

## Abstract

**Background:**

Type 1 diabetes (T1D) is a CD4^+^ T cell-driven autoimmune disease characterized by the destruction of insulin-producing pancreatic β-cells by CD8^+^ T cells. Achieving glycemic targets in T1D remains challenging in clinical practice; new treatments aim to halt autoimmunity and prolong β-cell survival. IMCY-0098 is a peptide derived from human proinsulin that contains a thiol-disulfide oxidoreductase motif at the N-terminus and was developed to halt disease progression by promoting the specific elimination of pathogenic T cells.

**Methods:**

This first-in-human, 24-week, double-blind phase 1b study evaluated the safety of three dosages of IMCY-0098 in adults diagnosed with T1D < 6 months before study start. Forty-one participants were randomized to receive four bi-weekly injections of placebo or increasing doses of IMCY-0098 (dose groups A/B/C received 50/150/450 μg for priming followed by three further administrations of 25/75/225 μg, respectively). Multiple T1D-related clinical parameters were also assessed to monitor disease progression and inform future development. Long-term follow-up to 48 weeks was also conducted in a subset of patients.

**Results:**

Treatment with IMCY-0098 was well tolerated with no systemic reactions; a total of 315 adverse events (AEs) were reported in 40 patients (97.6%) and were related to study treatment in 29 patients (68.3%). AEs were generally mild; no AE led to discontinuation of the study or death. No significant decline in C-peptide was noted from baseline to Week 24 for dose A, B, C, or placebo (mean change − 0.108, − 0.041, − 0.040, and − 0.012, respectively), suggesting no disease progression.

**Conclusions:**

Promising safety profile and preliminary clinical response data support the design of a phase 2 study of IMCY-0098 in patients with recent-onset T1D.

**Trial registration:**

IMCY-T1D-001: ClinicalTrials.gov NCT03272269; EudraCT: 2016–003514-27; and IMCY-T1D-002: ClinicalTrials.gov NCT04190693; EudraCT: 2018–003728-35.

**Supplementary Information:**

The online version contains supplementary material available at 10.1186/s12916-023-02900-z.

## Background

Type 1 diabetes (T1D) is a chronic autoimmune disease characterized by a loss of self-tolerance to autoantigens expressed by the insulin-producing β-cells in the pancreas. This leads to β-cell destruction, decline in endogenous insulin secretion, and consequent hyperglycemia [1]. T1D affects approximately 5–10% of people with diabetes; recent estimates suggest that approximately 5 million people worldwide will be diagnosed with T1D by 2050 [2–4]. The peak incidence of T1D is between 10 and 14 years, with half of the diagnoses made in individuals < 20 years old (T1D accounts for > 85% of all diabetes diagnoses in youth) [5].

Although a unique cause of T1D has not been identified, evidence suggests that T1D results from the interplay of a multigenic background and non-genetic components [6, 7]. Progression to absolute β-cell failure varies widely between individuals and may take several years [8]. While the exact triggers of the immune attack on β-cells are still unclear, it may arise from a combination of breaches in central and peripheral immune tolerance and interaction with the environment [9]. That adverse immune response in T1D can be directed against a number of self-antigens, including β-cell-specific preproinsulin, zinc transporter 8 (ZnT8), islet-glucose-6-phosphatase catalytic subunit-related protein, islet amyloid polypeptide and the more widely expressed glutamate decarboxylase (GAD65), insulinoma-associated antigen-2 (IA-2), glial fibrillary acidic protein and chromogranin A [8, 10]. Peptides derived from these antigens are presented on class I and class II major histocompatibility complex (MHC)/human leukocyte antigen (HLA) and recognized by autoreactive T lymphocytes. The highest level of genetic susceptibility to T1D is associated with DR3 and DR4 class II HLA haplotypes (odds ratio reaches 16.6 in DR3/DR4 heterozygote individuals) [11].

Despite the ongoing advances in T1D management, achieving good glycemic targets and preventing complications remains challenging [12, 13]. However, patients with persistent endogenous insulin secretion demonstrate improved metabolic control, less hypoglycemia, and lower risk of diabetic ketoacidosis [14–17]. Therefore, developing immunotherapies aimed at slowing the autoimmune process to achieve even minor degrees of β-cell preservation has received renewed attention.

General immunosuppression, which is partially effective in preserving β-cells from autoimmune destruction in recent-onset T1D, carries the risk of side effects that preclude its long-term use [18]. Antigen and peptide immunotherapy have been proposed as a strategy with a better risk/benefit ratio in humans. Results of early unsuccessful clinical studies that used GAD65-derived peptides suggested that peptide modification, selected administration routes and doses, and better patient selection should be explored in future studies of peptide immunotherapy for T1D [19]. Early clinical studies of different modified insulin-derived peptides demonstrated safety and variable efficacy in terms of clinical and immunological outcomes [20–23]. An immunodominant proinsulin peptide has proven to be well-tolerated and could potentially delay C-peptide decline [20]. The use of C-peptide as a surrogate for clinical outcomes in this setting was supported by a recent meta-analysis of four phase 2–3 randomized controlled trials in recent onset T1D, where the degree of preservation of C-peptide following treatment with GAD65 formulated with aluminum hydroxide (GAD-alum) was correlated with HbA1c changes [24].

Imotopes™ are linear synthetic peptides comprising an MHC/HLA class II-restricted T-cell epitope sequence linked to a thiol-disulfide oxidoreductase motif [25, 26]. In the non-obese diabetic mouse model, Imotope™ technology has been shown to generate antigen-specific cytolytic CD4^+^ T cells that have effector memory phenotype, express high levels of Granzyme B and are able to specifically eliminate antigen-presenting cells (APCs) that present this epitope [26]. Furthermore, these cytolytic CD4^+^ T cells eliminated autoreactive pathogenic T cells directed against this epitope and other epitopes presented on the same APC (bystander effect), thus protecting β-cells from autoimmune attack [25, 26].

**Fig. 1:**
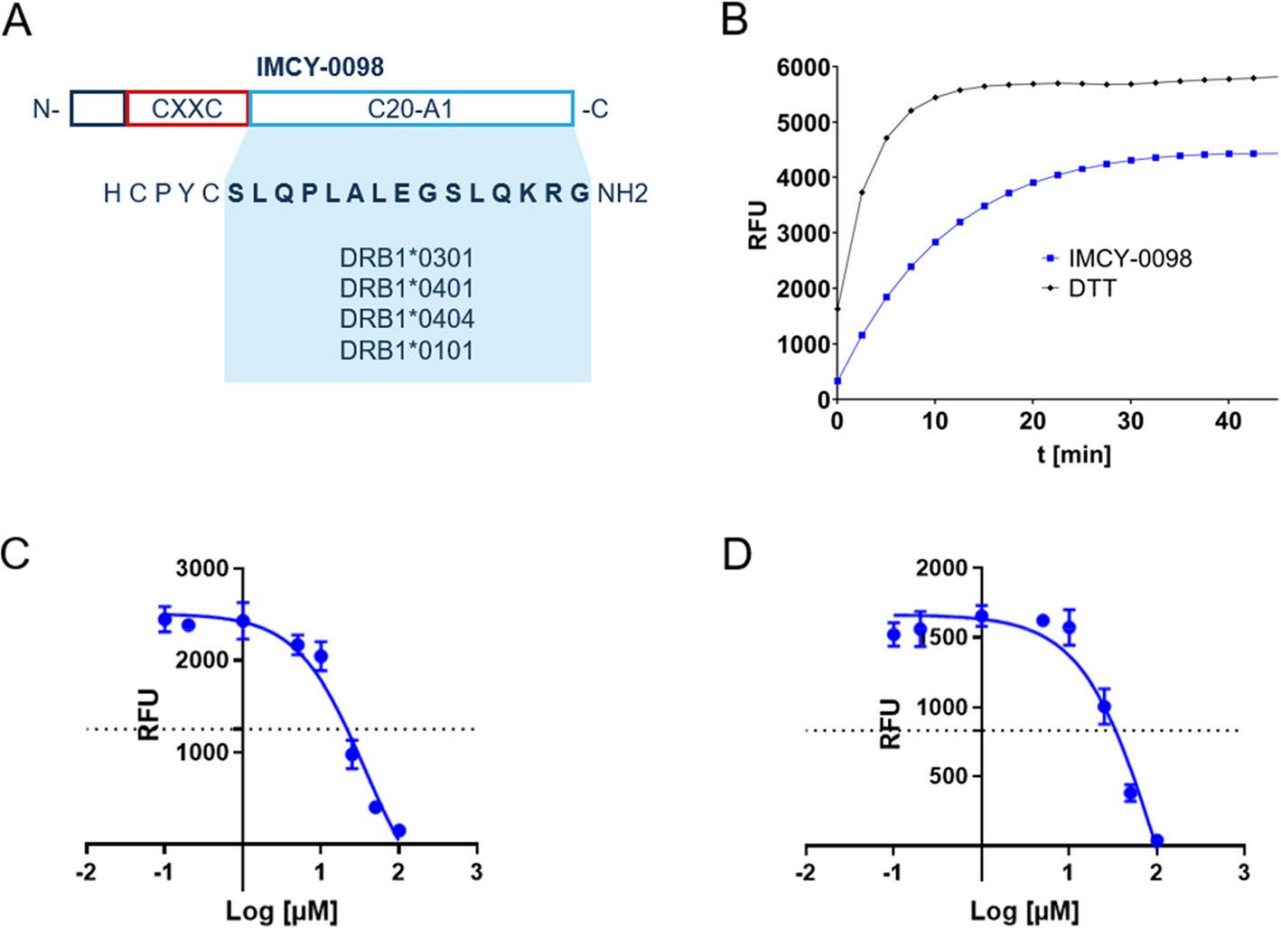
Figure to show summary of IMCY-0098 structure and the HLA class II epitopes it will bind based on previous literature [27] (**A**), in vitro thioredox data (**B**), in vitro binding data for DR3 (**C**), and DR4 (**D**). IMCY-009 sequence is shown as it overlaps with proinsulin (**A**). The design was based on previous literature [27]. Thiol-oxidoreductase activity was assessed in vitro on a disulfide-linked tripeptide substrate wherein a fluorescent signal was generated after disulfide bridge reduction using Sensolyte^®^ 520 Thiol Quantification kit. Results are expressed as a relative activity percentage compared to the dithiothreitol (DTT) after 45 min of incubation with the Thiol detection reagent (**B**). Binding affinities with DR3 and DR4 (**C** and **D**, respectively) were confirmed in a classical competition assay with a reference peptide (a high-affinity canonical 1–14 epitope derived from the YAR-B antigen), in which proinsulin-derived high-affinity binders would be identified

IMCY-0098 is the Imotope™ developed for T1D. The design of IMCY-0098 was based on previous literature [27] and in silico analysis, and was confirmed with in vitro thioredox and binding assays (Fig. 1). It was selected among a range of candidate peptides that were studied for their binding to HLA DR3 and DR4 class II molecules, which have a strong genetic link to T1D [11]. It contains the well-known C20-A1 sequence, a 15-amino acid peptide from the C domain of naturally processed proinsulin that has previously shown good binding capacity to the HLA-DRB1*0301 and HLA-DRB1*0401 (DR3 and DR4) polymorphisms, along with a proprietary thioreductase motif (Fig. 1.) [27]. The peptide has an amide group at the carboxyl terminus and a free amine at the amino terminus.

Here, we present the results of a first-in-human phase 1b study of Imotope™ technology platform using IMCY-0098 in patients with recent onset T1D across seven European countries (NCT03272269; EudraCT number 2016–003514-27). Alongside safety as a primary objective, multiple clinical response parameters were also examined to potentially inform future trials.

## Methods

### Study patients

This was a phase 1b, dose-escalation, randomized, placebo-controlled study of IMCY-0098 in patients with recent-onset T1D. Additional information on the conduct of the study can be found in the study protocol (Additional file 1). The study enrolled male and female participants 18–30 years of age who were diagnosed with T1D according to the American Diabetes Association/World Health Organization criteria within 6 months prior to participation in the study [28, 29]. The study included patients who were HLA DR3^+^ and/or DR4^+^; had ≥ 1 autoantibody against GAD65, IA-2, or ZnT8; and had fasting C-peptide at screening > 0.2 nmol/L and/or stimulated C-peptide ≥ 0.4 nmol/L. Key exclusion criteria were breastfeeding, pregnancy or planned pregnancy during the study, serious chronic liver conditions or hematologic disease, recent infection or antibiotic use, receipt of immunotherapy or any live, attenuated vaccine within 3 months before the first planned administration of study treatment, history of or current malignancy, or immune deficiency. Further information can be found in section 7.3.2 of the protocol (Additional file 1). Patients were recruited at 24 sites in 7 countries (Belgium, Denmark, France, Germany, Lithuania, Sweden, and the United Kingdom).

All participants provided signed informed consent. The study was conducted in accordance with the International Council for Harmonisation Good Clinical Practice guidelines, local national laws (as applicable), and the Declaration of Helsinki. The study was approved by Independent Ethics Committees according to local regulations across the participating countries. The study was registered on ClinicalTrials.gov (NCT03272269) and in the European Union Drug Regulating Authorities Clinical Trials Database (EudraCT number 2016–003514-27). A long-term follow-up of a subset of these patients was conducted under a separate protocol (ClinicalTrials.gov: NCT04190693; EudraCT number: 2018–003728-35).

### Study treatment

Study patients were randomly allocated in a 3:1 ratio to receive IMCY-0098 or placebo. Treatment allocation at each site was performed using central randomization via an Interactive Web Response System.

Treatment was injected subcutaneously in the upper arm (midway between the elbow and the shoulder); each dose was divided into two halves, which were administered concomitantly into both arms. Treatment was injected in both arms in order to stimulate the immune system via two draining lymph nodes and, consequently, to maximize the treatment-related immune response. Treatment was administered in four doses, given in 2-week intervals, with aluminum hydroxide adjuvant (alum) at a concentration of 500 μg/mL.

Patients allocated to receive IMCY-0098 were sequentially enrolled to receive dose A (first administration 50 μg, followed by 3 × 25 μg), dose B (first administration 150 μg, followed by 3 × 75 μg) or dose C (first administration 450 μg, followed by 3 × 225 μg). In each cohort, the first four patients stayed in the hospital for a 24-h safety follow-up after each administration, and an interval of 2 days was observed between these four patients and other patients enrolled to receive the same dose. Patients allocated to receive placebo received matching volumes of placebo solution to maintain blinding.

Interim safety evaluations were conducted by an independent Data and Safety Monitoring Board to allow inclusion of patients into the next cohort. Evaluations were conducted after four patients received their first administration and ≥ 3 patients received all four administrations of dose A (to allow inclusion of patients for dose B), and after four patients received their first administration and ≥ 3 patients received all four administrations of dose B (to allow inclusion of patients for dose C).

Data were collected in a double-blind manner until all patients completed the study to Week 24 or were prematurely withdrawn from the study. Patients could be withdrawn from the study due to an AE, development of illness, pregnancy, or loss to follow-up. If a patient’s body temperature was > 37.5 °C, injection of study treatment was to be postponed until the temperature was < 37.1 °C. Study treatment was to be discontinued in case of an anaphylactic reaction to administration, any AE Grade > 2 as defined by U.S. Department of Health and Human Services (grade 1 – mild; grade 2 – moderate; grade 3 – severe or medically significant; grade 4 – life-threatening; grade 5 – death related to AE) [30], a general immune disorder or if the patient was pregnant; these patients were encouraged to continue the other study procedures to ensure safety follow-up up to Week 24. Further information can be found in section 9.4.2 of the protocol (Additional file 1).

### Study objectives and endpoints

The primary objective was to assess the safety of IMCY-0098 at three different doses compared with placebo. Primary endpoints included adverse events (AEs), injection site reaction AEs, systemic AEs, serious adverse events (SAEs), laboratory abnormalities, vital signs (temperature, weight, systolic blood pressure, diastolic blood pressure, and heart rate), and fasting C-peptide levels. Fasting C-peptide was assessed at each visit to monitor disease progression and detect sudden short-term changes.

The secondary objective was to assess the clinical response to IMCY-0098 measured by disease activity. Secondary endpoints included post-challenge C-peptide (2 h after Mixed Meal Tolerance Test [MMTT]-stimulation), fasting C-peptide, glycated hemoglobin (HbA1c), insulin dose and glycemic profiles measured by the patient using the FreeStyle Libre Flash Glucose Monitoring System (i.e., frequency of hypoglycemic events [< 70 mg/dL] and to average glucose level in the last 14 days).

Exploratory endpoints to characterize the immune response to IMCY-0098 are under evaluation and will be published separately.

The main time point for assessment of primary and secondary endpoints was 24 weeks. More information can be found in Table 1 of the study protocol (Additional file 1). All patients who completed the study up to Week 24 and who were willing to participate were also included in a long-term follow-up of safety and underwent additional study visits at Week 36, and Week 48. The same primary endpoints as Week 24 were assessed and the same methodology was followed.

**Table 1:** Baseline patient and disease characteristics — safety analysis set

Characteristic	Placebo (*n* = 10)	IMCY-0098 dose A (*n* = 6)	IMCY-0098 dose B (*n* = 9)	IMCY-0098 dose C (*n* = 16)	Total (*N* = 41)
Age, years, mean ± SD	24.3 ± 3.37	22.2 ± 3.19	24.4 ± 4.53	24.2 ± 4.25	24.0 ± 3.91
Females:males^a^	5:5	2:4	2:7	4:12	13:28
BMI, kg/m^2^, mean ± SD	24.2 ± 2.73	23.3 ± 2.86	22.7 ± 2.56	22.9 ± 3.33	23.2 ± 2.92
Glycemic parameters at baseline, mean ± SD					
MMTT: C-peptide C_max_, nmol/L	0.867 ± 0.2	0.65 ± 0.221	0.873 ± 0.342	0.781 ± 0.251	0.803 ± 0.261
MMTT: Glucose at start, mmol/L	6.9 ± 1.19	7.9 ± 1.63	6.2 ± 1.24	7.0 ± 1.21	6.9 ± 1.33
MMTT: C-peptide normalized AUC, nmol/L	0.694 ± 0.218	0.515 ± 0.157	0.672 ± 0.278	0.585 ± 0.179	0.621 ± 0.213
Blood glucose, mmol/L^b^	7.06 ± 1.49	8.11 ± 1.29	6.7 ± 1.17	7.5 ± 1.47	7.31 ± 1.41
C-peptide/glucose ratio, nmol/mol^b^	49.9 ± 16.4	36.2 ± 14.5	54.9 ± 23.6	43.7 ± 11.6	46.6 ± 16.9
HbA1c, mmol/L	7.18 ± 1.299	7.45 ± 0.841	6.87 ± 1.442	6.72 ± 0.810	6.98 ± 1.108
Number of autoantibodies against GAD65, IA-2, or ZnT8, *n* (%)^a^					
1	4 (40.0)	1 (16.7)	1 (11.1)	2 (12.5)	8 (19.5)
2	3 (30.0)	0 (0.0)	3 (33.3)	5 (31.2)	11 (26.8)
3	3 (30.0)	5 (83.3)	5 (55.6)	9 (56.3)	22 (53.7)
Autoantibodies against insulin, *n* (%)	5 (50.0)	1 (16.7)	4 (44.4)	10 (62.5)	20 (48.8)
Time from diagnosis to first dose, days, mean ± SD	112 ± 51	106 ± 55	102 ± 52	116 ± 46	110 ± 49
HLA status, *n* (%)^a^					
DR3^+^/DR4^−^	2 (20.0)	2 (33.3)	1 (11.1)	5 (31.2)	10 (24.4)
DR4^+^/DR3^−^	2 (20.0)	3 (50.0)	6 (66.7)	8 (50.0)	19 (46.3)
DR3^+^/DR4^+^	6 (60.0)	1 (16.7)	2 (22.2)	3 (18.8)	12 (29.3)

Dose A: 50 μg at week 0 followed by 3 × 25 μg; dose B: 150 μg at week 0 followed by 3 × 75 μg; dose C: 450 μg at week 0 followed by 3 × 225 μg

*AUC* area under the curve, *BMI* body mass index, *C*_*max*_ maximum concentration, *GAD* glutamic acid decarboxylase, *HLA* human leukocyte antigen, *MMTT* Mixed Meal Tolerance Test, *SD* standard deviation

^a^These characteristics were imbalanced across treatment arms

^b^Mean value was calculated between Visit 1 (4 weeks before baseline) and visit 2 (baseline; time of first injection)

### Study procedures and assessments

#### Safety

Study visits and procedures are shown in Additional file 2: Fig. S1. Recording current insulin doses, complete physical examination, vital signs, and safety assessments were performed at each visit. AEs were described and coded according to the Medical Dictionary for Regulatory Activities (MedDRA). Solicited AEs were defined as any of the event types prespecified in the protocol and were collected in a solicited manner in the case report form and on the diary card, where the patient had to record the presence/absence of each event every day as well as the intensity, during a 7 day follow up period (the day of study product administration and the following 6 days). Solicited AEs were: injection site AEs, pain/tenderness, itching, swelling, redness, induration, and the systemic AEs (fever, headache, fatigue, malaise, and myalgia). Unsolicited AEs were defined as any event that was not prespecified. Solicited AEs that occurred outside of the 7-day follow-up period or with a duration longer than 7 days were recorded as unsolicited AEs. All AEs were graded according to the Common Terminology Criteria for Adverse Events (CTCAE) approach defined by the U.S. Department of Health and Human Services as specified in the protocol [30]. AEs were considered to be SAEs if any of the following criteria were met: an event that required hospitalization or prolongation of existing hospitalization, an event that resulted in disability, an event that caused congenital anomaly or birth defect in the offspring of a participant, a life-threatening event, death, or an event that jeopardized the patient and required medical or surgical intervention to prevent one of the other outcomes listed. More information can be found in section 11.2 of the protocol (Additional file 1).

The intensity of injection site reactions was determined by study center staff for the first four patients of each cohort, either by visual assessment or through questioning the patient, and self-reported by the rest of the patients. Injection site erythema/redness, inflammation/swelling, and induration were classified using the longest diameter of the affected skin area as mild (grade 1; 0–30 mm), moderate (grade 2; 30–120 mm), or severe (grade 3; ≥ 120 mm). Pain/tenderness was classified based on patient perception as mild (grade 1; injection site is painful when pressed), moderate (grade 2; interferes with activity) or severe (grade 3; prevents daily activity). Itching was classified as mild (grade 1; mild or localized), moderate (grade 2; intense or widespread, intermittent, limiting instrumental activities of daily living) or severe (grade 3; intense or widespread, constant, limiting self-care, instrumental activities of daily living or sleep). Blood samples were taken at each visit for clinical chemistry and hematology assessment, and were analyzed with standardized techniques.

#### Clinical response

Stimulated C-peptide secretion was assessed by the 2-h MMTT method at screening, Week 12, and Week 24. MMTT was performed in fasting patients using Ensure Plus (220 mL; 330 kcal; Abbott Laboratories) as a standardized meal administered between 07:00 h and 10:00 h, with 5 collection time points (before the meal and 30 ± 3 min, 60 ± 3 min, 90 ± 5 min and 120 ± 5 min after the meal). Patients were asked to withhold taking slow-acting insulin on the morning of the test but were allowed to take prandial insulin up to 2 h before the test. An additional clinical parameter was derived based on the difference between the normalized area under the curve (AUC) measured during MMTT tests and the AUC expected values given the general disease progression, as described previously [31].

Antibody levels were assessed using blood samples collected at screening and at Weeks 0, 6, 12, and 24. Autoantibodies directed against insulin, IA-2, and GAD were detected using radioimmunoassays (Euroimmun, Lubeck, Germany) and a gamma counter; autoantibodies directed against ZnT8 were detected using enzyme-linked immunosorbent assay (RSR Limited, Cardiff, UK) and Tecan microplate reader (Tecan Group, Manedorf, Switzerland); all assays were used according to manufacturers’ instructions.

### Statistical analysis

The study sample size was determined as adequate to provide a reliable safety assessment of the tested doses and to support the preliminary assessment of clinical response endpoints. The sample size used in this study was aligned with a similar study of peptide immunotherapy which found the safety profile to be as expected [20]. The sample size favored a higher patient number in the higher dose group as this dose was expected to achieve higher immunogenicity (exploratory endpoint), versus the low dose group. This approach was fully endorsed by seven regulatory authorities. All patients who received at least one dose of study treatment were included in the safety analysis set. All patients who received at least one dose of study treatment and had at least one post-dose clinical assessment were included in the intent-to-treat (ITT) analysis set. The results were summarized by descriptive statistics (continuous variables) or frequency tables (categorical variables), by dose and overall. There was no stratification according to DR3/DR4 haplotype.

All clinical response analyses were performed by dose and overall. For MMTT analysis, changes in the area under the curve (AUC) C-peptide from baseline at each visit were analyzed using a one-way analysis of covariance (ANCOVA) method. Fasting C-peptide was normalized by the glucose level and changes in fasting C-peptide and change in HbA1c were analyzed using one-way ANCOVA (F test). Change in insulin dose was analyzed using a *t*-test. For all clinical response endpoints, dose comparison was performed using a two-sided 95% confidence interval (CI) and *p*-value for the between-group differences in least squares means at Week 24.

Disease progression was measured as a primary endpoint. For this analysis, a linear mixed effect model (random effect – inter-patient variability; fixed effects – all available covariates) was used to model the progression of endpoint fasting C-peptide over time. Covariates were chosen from a list of candidates (baseline laboratory values, autoantibodies levels, HLA status, and patient characteristics); as there were multiple candidates, there were multiple possible combinations of covariates. Only covariates with strong associations with C-peptide/glucose at least at two visits were included. The combination of covariates was selected via a data-driven approach: they were retained if significantly associated with C-peptide/glucose change from baseline at Week 24 (*p* < 0.05 and Cohen’s index ≥ 0.8) in at least one subgroup (defined as a group of patients that carry a given level of each covariate). The best model was selected according to the Akaike Information Criterion which quantifies the quality of statistical models [32]. The model predicts the progression of C-peptide/glucose over time while taking into account the treatment arm, inter-subject variability, and fixed effect of seven additional covariates. Regression coefficients were analyzed via the Mann–Whitney-Wilcoxon test.

Autoantibody levels were analyzed separately for each autoantibody using an ANCOVA model with ratio to baseline as dependent variable, baseline value as covariate, and treatment as factor. The ratios between each treatment group and placebo were analyzed using confidence interval and *p*-value for the test of no difference. Only significant *p*-values (< 0.05) will be presented for clarity.

## Results

### Study patients

**Fig. 2:**
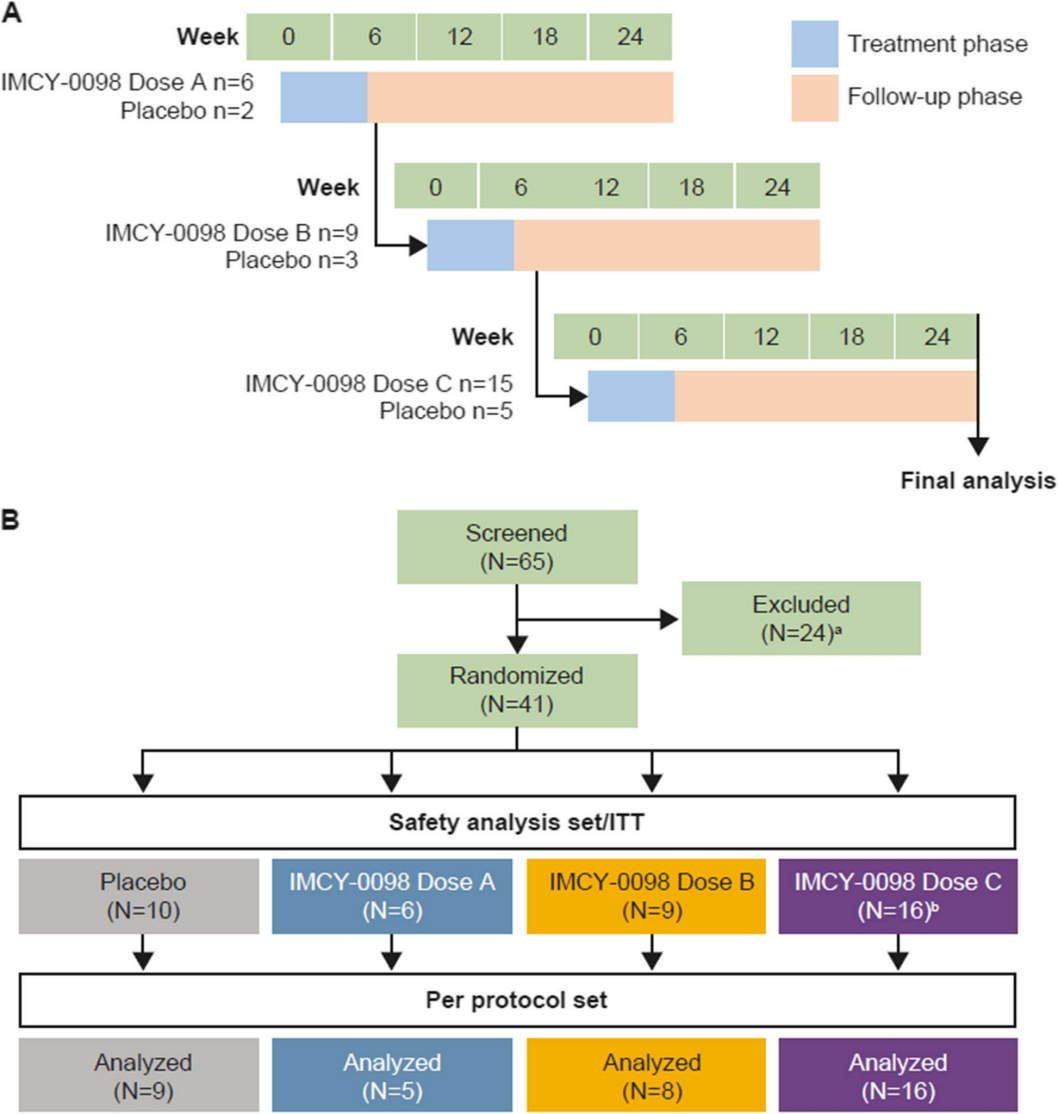
Figure to show the sequential study design (**A**) and patient disposition (**B**). Safety analysis set and ITT population were the same in this study. Per protocol set excluded three patients who received the incorrect dose of study medication at one of the four injections (one patient allocated to placebo treatment arm erroneously received IMCY-0098). ^a^Patients were excluded for the following reasons: HLA status (*n* = 10), withdrawal of consent (*n* = 7), C-peptide level (*n* = 3); time post-diagnosis (*n* = 2), autoantibody status (*n* = 1) and body-mass index (*n* = 1). ^b^A total of 15 patients were planned for dose C; however, 16 patients were randomized to receive this dose. dose A: 50 μg at week 0 followed by 3 × 25 μg; dose B: 150 μg at week 0 followed by 3 × 75 μg; dose C: 450 μg at week 0 followed by 3 × 225 μg. ITT, intent-to-treat. *N*/*n* refer to patients

Of 65 screened patients with recent-onset type 1 diabetes, 41 were randomized to receive placebo (*n* = 10) or IMCY-0098 dose A (*n* = 6), dose B (*n* = 9), or dose C (*n* = 16) (Fig. 2; Table 1). Three patients received the incorrect dose of study medication at one of the four injections and were excluded from the per protocol analysis (Fig. 2).

The mean age was 24.0 years, and 31.7% of all participants were women. Mean age, BMI, and time from diagnosis were similar across cohorts; however, there were differences in cohort composition in terms of gender and the proportion of patients with different numbers of autoantibodies. There were also differences in HLA haplotype across cohorts, with DR3^+^/DR4^+^ patients overrepresented in the placebo group. The baseline characteristics are summarized in Table 1.

### Safety

Safety results are presented in Table 2. A total of 315 AEs were reported in 40 patients (97.6%); AEs were considered related to study treatment in 28 patients (68.3%; [60%, 66.7%, 77.8%, and 68.8% in patients receiving placebo IMCY-0098 dose A, B, and C, respectively]), Additional file 2: Table S1. AEs were generally mild and of short duration in all treatment arms, none led to treatment discontinuation; there were no deaths. Twenty patients (48.8%) experienced injection site reactions. The safety profile was as expected when injecting an alum compound subcutaneously (pain, itching, and swelling at the injection site) [33]. No inferential statistical analysis was performed for adverse events due to the small sample size.

**Table 2:** Summary of adverse events — safety analysis set

AEs, *n* (%) [number of events]	Placebo (*n* = 10)	IMCY-0098 dose A (*n* = 6)	IMCY-0098 dose B (*n* = 9)	IMCY-0098 dose C (*n* = 16)	Total (*N* = 41)
All AEs	9 (90.0) [51]	6 (100) [38]	9 (100) [85]	16 (100) [141]	40 (97.6) [315]
Solicited AEs	7 (70.0) [24]	3 (50.0) [19]	7 (77.8) [40]	12 (75.0) [72]	29 (70.7) [156]
Unsolicited AEs	8 (80.0) [27]	6 (100) [19]	9 (100) [43]	16 (100) [69]	39 (95.1) [159]
AEs by grade					
Grade 1	5 (50.0)	5 (83.3)	4 (44.4)	6 (37.5)	20 (48.8)
Grade 2	3 (30.0)	1 (16.7)	5 (55.6)	10 (62.5)	19 (46.3)
Grade 3	1 (10.0)	0	0	0	1 (2.4)
Grade 4	0	0	0	0	0
Treatment-related AEs	6 (60.0) [25]	4 (66.7) [18]	7 (77.8) [41]	11 (68.8) [65]	28 (68.3) [150]
Injection site reactions	5 (50.0) [18]	3 (50.0) [12]	4 (44.4) [26]	8 (50.0) [50]	20 (48.8) [106]
Injection site pain	3 (30.0) [3]	3 (50.0) [9]	3 (33.3) [8]	6 (37.5) [28]	15 (36.6) [47]
Injection site erythema	3 (30.0) [11]	1 (16.7) [1]	2 (22.2) [9]	4 (25.0) [6]	10 (24.4) [27]
Injection site induration	1 (10.0) [1]	0	1 (11.1) [4]	3 (18.8) [5]	5 (12.2) [10]
Injection site pruritus	1 (10.0) [1]	1 (16.7) [1]	1 (11.1) [3]	2 (12.5) [4]	5 (12.2) [9]
Injection site swelling	0	1 (16.7) [1]	1 (11.1) [1]	2 (12.5) [3]	4 (9.8) [5]
Injection site bruising	0	0	1 (11.1) [1]	3 (18.8) [3]	4 (9.8) [4]
Injection site reaction	1 (10.0) [2]	0	0	0	1 (2.4) [2]
Injection site irritation	0	0	0	1 (6.3) [1]	1 (2.4) [1]
SAEs	0	0	1 (11.1) [2]^a^	1 (6.3) [1]	2 (4.9) [3]
Phimosis	0	0	1 (11.1) [1]	0	1 (2.4) [1]
Varicocele	0	0	1 (11.1) [1]	0	1 (2.4) [1]
Vestibular neuronitis	0	0	0	1 (6.3) [1]	1 (2.4) [1]
AEs leading to drug withdrawal	0	0	0	0	0
AEs leading to death	0	0	0	0	0

Dose A: 50 μg at week 0 followed by 3 × 25 μg; dose B: 150 μg at week 0 followed by 3 × 75 μg; dose C: 450 μg at week 0 followed by 3 × 225 μg

*AE* adverse event, *SAE* serious adverse event

^a^Both SAEs occurred in the same patient and were associated with surgery for a pre-existing condition

Solicited AEs included a selection of specific AE terms listed in the case report form and patient’s diary card (injection site reactions and systemic reactions, such as headache, fatigue, malaise, myalgia, and fever). Unsolicited AEs included any events that were not listed and any solicited AEs that occurred outside of the 7-day follow-up period. AEs were graded according to the Common Terminology Criteria for Adverse Events (CTCAE) approach defined by the US Department of Health and Human Services [30]. Both solicited and unsolicited AEs were evaluated for their relationship to study treatment. Treatment-related AEs were defined as those that were possibly related (unlikely related but could not be ruled out) or probably related (considered related with a high degree of certainty) to the study product

Three SAEs were recorded in two patients; one SAE was classified as possibly related to study treatment: a case of vestibular neuronitis in a patient who received Dose C of IMCY-0098, occurring 4 months after the last injection of study treatment. The patient was hospitalized and received treatment with intravenous methylprednisolone, oral acetyl-leucine, and oral domperidone. The event resolved without sequelae within 1 week. The principal investigator reviewing the event considered it moderate in intensity and possibly related to the study treatment. However, the length of the time-to-onset since the study treatment did not support a cause-and-effect relationship. Additionally, the subject reported feeling unwell the day before the event onset and so this event appeared more suggestive of a viral infection; the most common known etiology for vestibular neuronitis. The sponsor therefore assessed the event as unlikely related to IMCY-0098, based upon considerations of biological plausibility.

Clinical chemistry results are presented in Table 3 and hematology results are presented in Table 4. Hematology data showed no marked changes in any type of white blood cell, indicating an absence of general immune suppression, reflected in an absence of opportunistic infections. There were also no marked changes in vital signs data (Additional file 2: Table S2).

**Table 3:** Change in clinical chemistry parameters from screening to week 24 — safety analysis set

Parameter, screening mean, ± SD; [and change at week 24]	Placebo (*n* = 10)	IMCY-0098 dose A (*n* = 6)	IMCY-0098 dose B (*n* = 9)	IMCY-0098 dose C (*n* = 16)	Total (*N* = 41)
Alanine aminotransferase U/L	24.9, 10.0 [− 0.3, 12.1]	18.0, 5.7 [6.5, 9.2]	22.6, 11.5 [− 1.8, 7.7]	26.8, 23.7 [− 2.3, 17.0]	24.1, 16.5 [− 0.4,13.1]
Aspartate aminotransferase, U/L	23.4, 5.8 [− 4.9, 12.0]	21.3, 4.1 [1.2, 8.2]	28.1, 9.6 [− 5.4, 4.8]	24.0, 9.2 [− 0.3, 8.1]	24.4, 8.0 [− 2.3, 8.8]
Lactate dehydrogenase, U/L	143.1, 17.7 [− 6.6, 12.7]	142.5, 17.1 [14.7, 29.6]	154.0, 22.3 [− 17.0, 16.6]	152.5, 27.2 [− 2.1, 27.2]	149.1, 22.6 [− 4.0, 23.9]
Creatine kinase, U/L	98.8, 66.0 [− 217.7, 498.3]	109.5, 83.0 [− 11.7, 117.1]	262.6, 355.0 [− 74.6, 112.7]	160.1, 161.1 [35.8, 123.1]	160.2, 201.2 [− 57.2, 275.8]
Alkaline phosphatase, U/L	69.2, 20.1 [− 2.0, 19.8]	65.0, 11.4 [− 3.7, 3.4]	69.9, 13.4 [− 5.9, 10.2]	60.7, 17.3 [0.9, 10.1]	65.4, 16.5 [− 2.0, 12.5]
Bilirubin, µmol/L	8.2, 6.9 [− 1.9, 3.7]	7.4, 3.1 [− 0.1, 3.2]	6.6, 3.5 [0.9, 3.3]	15.9, 13.2 [− 2.9, 7.0]	10.8, 9.9 [− 1.4, 5.2]
Creatinine, µmol/L	70.5, 15.1 [2.91, 7.8]	65.6, 6.6 [1.2, 3.1]	79.4, 22.4 [− 0.78, 11.5]	73.6, 9.2 [− 1.5, 6.8]	73.0, 14.5 [0.13, 7.9]
Urea, mmol/L	4.6, 1.4 [− 0.5, 0.6]	4.2, 0.8 [− 0.1, 1.1]	5.8, 1.6 [− 1.5, 1.2]	4.2, 1.2 [− 0.2, 1.1]	4.7, 1.4 [− 0.5, 1.1]
Albumin, g/L	47.8, 3.6 [− 0.9, 3.4]	47.2, 2.1 [1.7, 2.7]	47.3, 3.5 [− 0.7, 2.8]	47.5, 3.2 [− 0.8, 3.2]	47.5, 3.1 [− 0.4, 3.1]
Gamma glutamyl transferase, U/L	20.1, 14.8 [3.7, 11.5]	15.5, 5.4 [2.0, 5.6]	15.1, 5.5 [0.1, 3.5]	14.0, 5.5 [− 1.6, 3.7]	16.0, 8.8 [0.6, 6.8]
Glucose, mmol/L	7.0, 1.1 [− 0.5, 2.3]	7.9, 1.6 [− 0.9, 1.7]	6.8, 2.3 [0.4, 1.8]	7.1, 1.2 [0.1, 2.9]	7.1, 1.5 [− 0.1, 2.4]

**Table 4:** Change in hematology parameters relative to normal range from screening to week 24 — safety analysis set

**Parameter,* n* (%)**		**Placebo (*n* = 10)**	**IMCY-0098 dose A (*n* = 6)**	**IMCY-0098 dose B (*n* = 9)**	**IMCY-0098 dose C (*n* = 16)**	**Total (*N* = 41)**
Erythrocytes (10^12/L)	*n* [1]	10	6	9	16	41
	Decrease to low	0	0	4 (44.4)	3 (18.8)	7 (17.1)
	Change to normal or no change	10 (100)	6 (100)	5 (55.6)	13 (81.3)	34 (82.9)
	Increase to high	0	0	0	0	0
Monocytes (10^9/L)	*n* [1]	10	6	9	16	41
	Decrease to low	0	0	2 (22.2)	0	2 (4.9)
	Change to normal or no change	9 (90.0)	6 (100)	6 (66.7)	14 (87.5)	35 (85.4)
	Increase to high	1 (10.0)	0	1 (11.1)	2 (12.5)	4 (9.8)
Lymphocytes/leukocytes (%)	*n* [1]	10	6	9	16	41
	Decrease to low	2 (20.0)	0	1 (11.1)	2 (12.5)	5 (12.2)
	Change to normal or no change	6 (60.0)	4 (66.7)	7 (77.8)	12 (75.0)	29 (70.7)
	Increase to high	2 (20.0)	2 (33.3)	1 (11.1)	2 (12.5)	7 (17.1)
Monocytes/leukocytes (%)	*n* [1]	10	6	9	16	41
	Decrease to low	1 (10.0)	1 (16.7)	0	0	2 (4.9)
	Change to normal or no change	8 (80.0)	3 (50.0)	9 (100)	15 (93.8)	35 (85.4)
	Increase to high	1 (10.0)	2 (33.3)	0	1 (6.3)	4 (9.8)
Granulocytes/leukocytes (%)	*n* [1]	0	0	0	1	1
	Decrease to low	0	0	0	0	0
	Change to normal or no change	0	0	0	1 (100)	1 (100)
	Increase to high	0	0	0	0	0
Neutrophile granulocytes (10^9/L)	*n* [1]	10	6	9	16	41
	Decrease to low	1 (10.0)	1 (16.7)	1 (11.1)	1 (6.3)	4 (9.8)
	Change to normal or no change	7 (70.0)	5 (83.3)	7 (77.8)	12 (75.0)	31 (75.6)
	Increase to high	2 (20.0)	0	1 (11.1)	3 (18.8)	6 (14.6)
Eosinophile granulocytes (10^9/L)	*n* [1]	10	6	9	16	41
	Decrease to low	1 (10.0)	0	0	1 (6.3)	2 (4.9)
	Change to normal or no change	8 (80.0)	6 (100)	8 (88.9)	14 (87.5)	36 (87.8)
	Increase to high	1 (10.0)	0	1 (11.1)	1 (6.3)	3 (7.3)
Bas. granulocytes (10^9/L)	*n* [1]	10	6	9	16	41
	Decrease to low	0	0	0	0	0
	Change to normal or no change	9 (90.0)	4 (66.7)	8 (88.9)	14 (87.5)	35 (85.4)
	Increase to high	1 (10.0)	2 (33.3)	1 (11.1)	2 (12.5)	6 (14.6)
Neutrophile granulocytes/leukocytes (%)	*n* [1]	10	6	9	16	41
	Decrease to low	0	0	1 (11.1)	1 (6.3)	2 (4.9)
	Change to normal or no change	9 (90.0)	6 (100)	8 (88.9)	13 (81.3)	36 (87.8)
	Increase to high	1 (10.0)	0	0	2 (12.5)	3 (7.3)
Neutr. granulocytes/leukocytes (%)	*n* [1]	0	2	0	1	3
	Decrease to low	0	1 (50.0)	0	0	1 (33.3)
	Change to normal or no change	0	1 (50.0)	0	1 (100)	2 (66.7)
	Increase to high	0	0	0	0	0
Eosinophile granulocytes/leukocytes (%)	*n* [1]	10	6	9	16	41
	Decrease to low	1 (10.0)	0	0	1 (6.3)	2 (4.9)
	Change to normal or no change	7 (70.0)	5 (83.3)	9 (100)	14 (87.5)	35 (85.4)
	Increase to high	2 (20.0)	1 (16.7)	0	1 (6.3)	4 (9.8)
Eosin. granulocytes/leukocytes (%)	*n* [1]	0	2	0	1	3
	Decrease to low	0	0	0	0	0
	Change to normal or no change	0	2 (100)	0	1 (100)	3 (100)
	Increase to high	0	0	0	0	0
Basophile granulocytes/leukocytes (%)	*n* [1]	0	2	0	0	2
	Decrease to low	0	0	0	0	0
	Change to normal or no change	0	0	0	0	0
	Increase to high	0	2 (100)	0	0	2 (100)
Bas. granulocytes/leukocytes (%)	*n* [1]	10	6	9	16	41
	Decrease to low	0	0	0	0	0
	Change to normal or no change	10 (100)	5 (83.3)	8 (88.9)	16 (100)	39 (95.1)
	Increase to high	0	1 (16.7)	1 (11.1)	0	2 (4.9)
Lymphocytes atypical/leukocytes (%)	*n* [1]	0	2	0	1	3
	Decrease to low	0	0	0	0	0
	Change to normal or no change	0	0	0	0	0
	Increase to high	0	2 (100)	0	1 (100)	3 (100)
Smudge cells/leukocytes (%)	*n* [1]	0	2	0	0	2
	Decrease to low	0	0	0	0	0
	Change to normal or no change	0	2 (100)	0	0	2 (100)
	Increase to high	0	0	0	0	0

*n *[1], subjects with at least one report

**Fig. 3:**
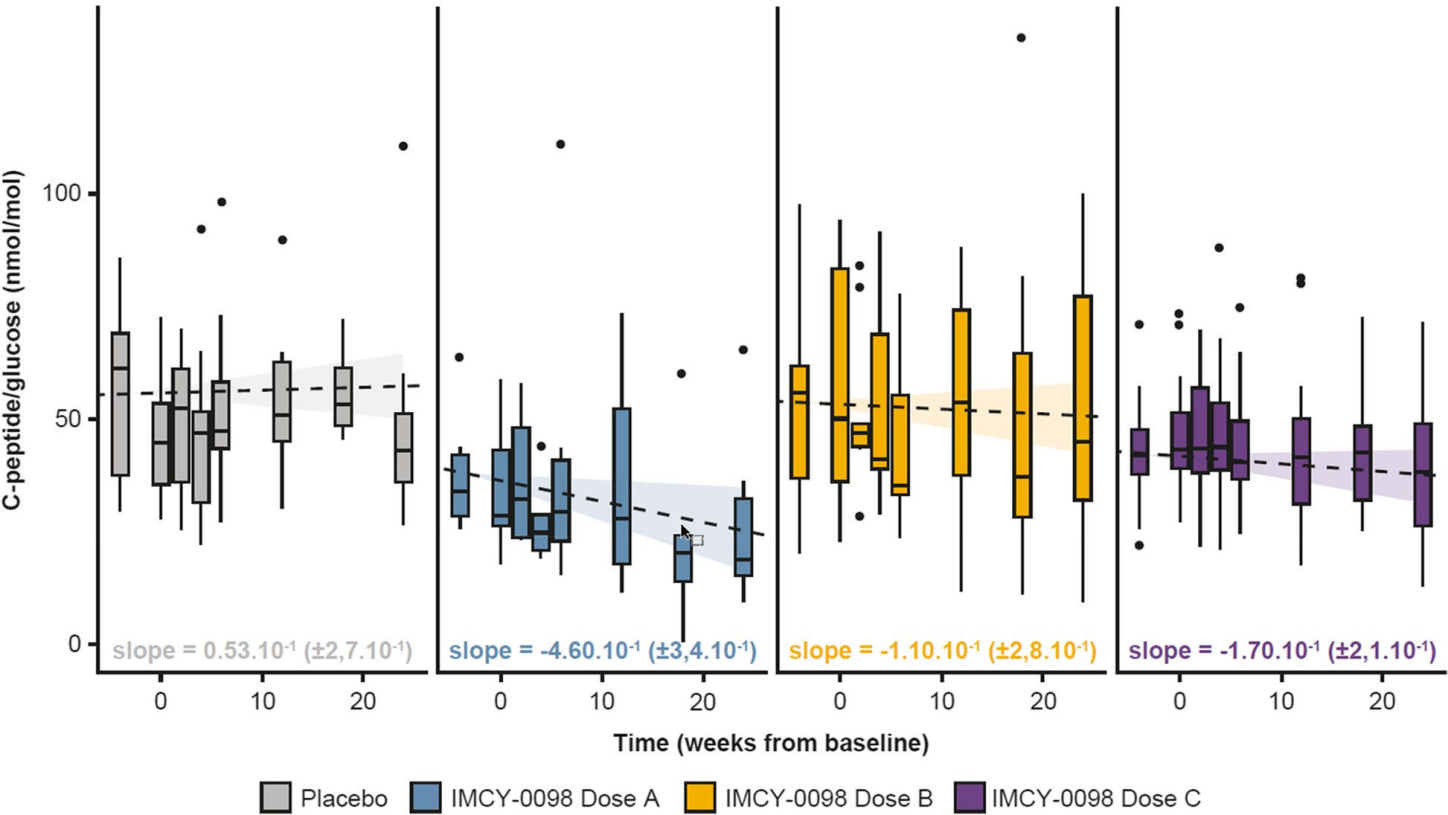
Linear mixed effect model to show C-peptide/glucose progression over time in the intent-to-treat population. Shown is the best of tested linear mixed effect models selected according to the Akaike Information Criterion. The model predicts the progression of C-peptide/glucose over time while taking into account treatment arm, inter-subject variability and fixed effect due to seven additional covariates: C-peptide/glucose at baseline, fasting C-peptide at baseline, glycemia at baseline, HLA-DQ8 status, HLA-DR3/HLA-DQ2 status, HLA-DR4 status, and gender. Regression coefficients were analyzed via Mann–Whitney-Wilcoxon test. Central lines represent median values, boxes represent interquartile range, and whiskers represent upper and lower 1.5 × interquartile range. Shaded bands around the regression lines represent 95% confidence intervals on the fitted values. The ranges displayed in brackets are 95% confidence intervals, which were assessed by computing a likelihood profile and finding the appropriate cutoffs based on the likelihood ratio test. All plotted data are biological replicates. Dose A: 50 μg at week 0 followed by 3 × 25 μg; dose B: 150 μg at week 0 followed by 3 × 75 μg; dose C: 450 μg at week 0 followed by 3 × 225 μg

Disease progression was assessed using the change in fasting C-peptide/glucose ratio during the study. To account for inter-subject variability, this change was assessed with a linear mixed effect model that took into account the interaction between time and the treatment arm, and an adjustment based on 7 covariates including gender, C-peptide, and glucose values at baseline and HLA type (Fig. 3). Out of all the combinations of candidate covariates, these were selected using the Akaike Information Criterion. The regression coefficients showed no disease progression across treatment arms: the fasting C-peptide/glucose ratio remained stable for all doses, from placebo (+ 0.11 [± 0.54)] %/day) to dose C (− 0.39 [± 0.48] %/day).

### Clinical response

**Fig. 4:**
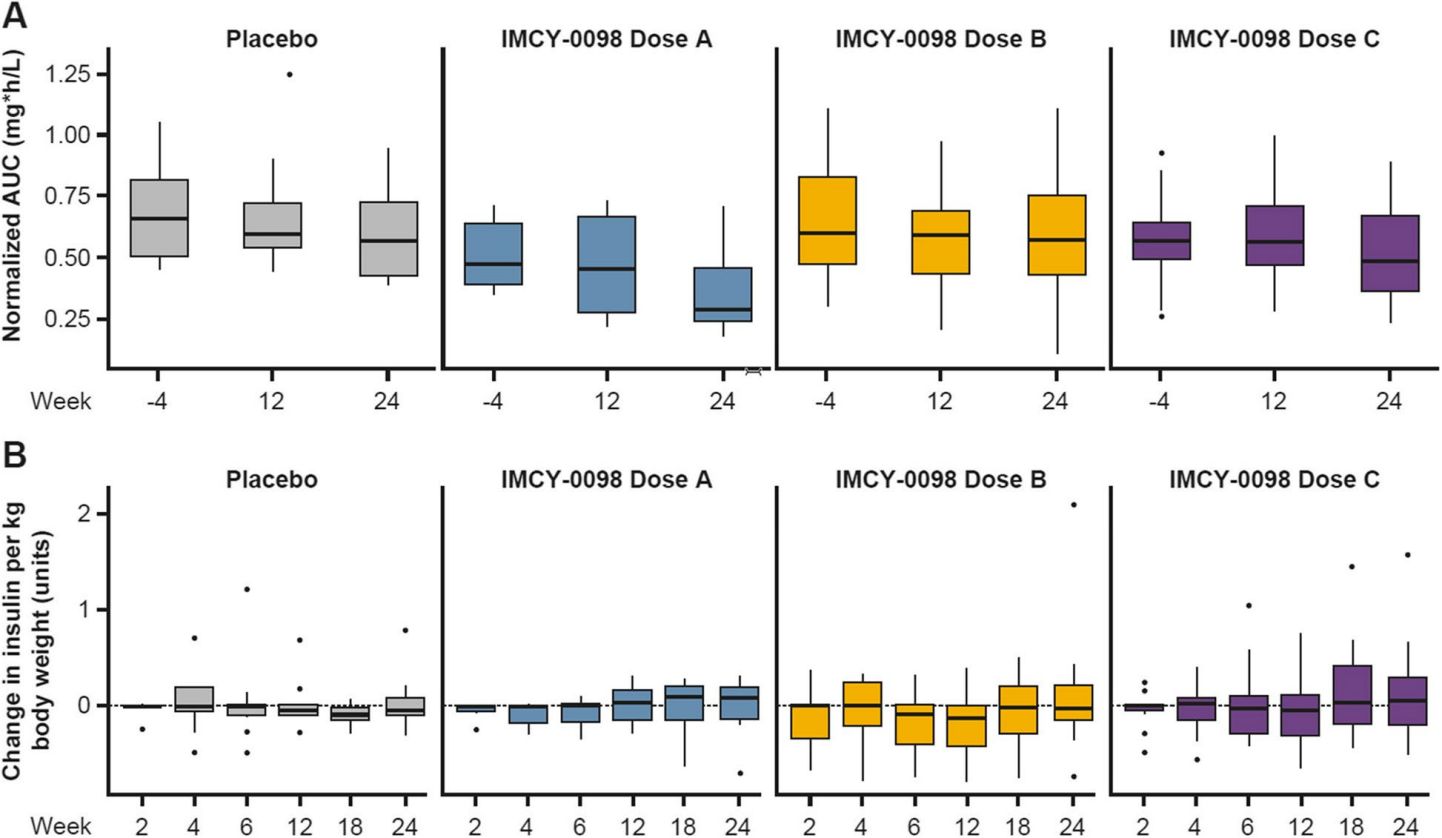
Progression of clinical response measured by AUC C-peptide from MMTT (**A**) and daily insulin consumption (**B**). Data are shown for the intent-to-treat population. Central lines represent median values, boxes represent interquartile range, and whiskers represent upper and lower 1.5 × interquartile range, respectively. Dose A: 50 μg at Week 0 followed by 3 × 25 μg; dose B: 150 μg at Week 0 followed by 3 × 75 μg; dose C: 450 μg at Week 0 followed by 3 × 225 μg. Normalized AUC refers to C-peptide normalized to glucose. All plotted data are biological replicates. Points represent subjects. AUC, area under the curve; MMTT, Mixed Meal Tolerance Test

In the ITT set, clinical response was assessed using the Mixed Meal Tolerance Test (MMTT) and total daily consumption of insulin (both slow- and fast-acting) (Fig. 4, Additional file 2: Tables S3 and S4). There were no statistically significant differences with these endpoints across study arms, as anticipated given the small patient number and the short follow-up time in an adult population. These results confirmed the absence of disease progression after treatment with IMCY-0098 as there was no acceleration in C-peptide decrease or significant increase in insulin dose as would have been expected.

During the study period, the levels of autoantibodies against GAD65 and ZnT8 decreased in IMCY-0098 dose C arm, and the level of autoantibodies against IA-2 decreased in IMCY-0098 dose A arm; however, the differences between treatment arms and placebo were not statistically significant, with the exception of ZnT8 at Week 24 (Additional file 2: Fig. S2).

The estimated mean change from baseline to Week 24 in fasting C-peptide were − 0.012 for placebo and − 0.108, − 0.041, and − 0.040 for the IMCY-0098 dose A, B, and C groups, respectively. When comparing each IMCY-0098 dose group against placebo, none of the three doses gave a significant change in fasting C-peptide at the 5% significance level. The estimated mean change from baseline to Week 24 in HbA1c was − 0.528 for placebo and − 0.049, − 0.254, and 0.104 for the IMCY-0098 dose A, B, and C groups, respectively.

**Fig. 5:**
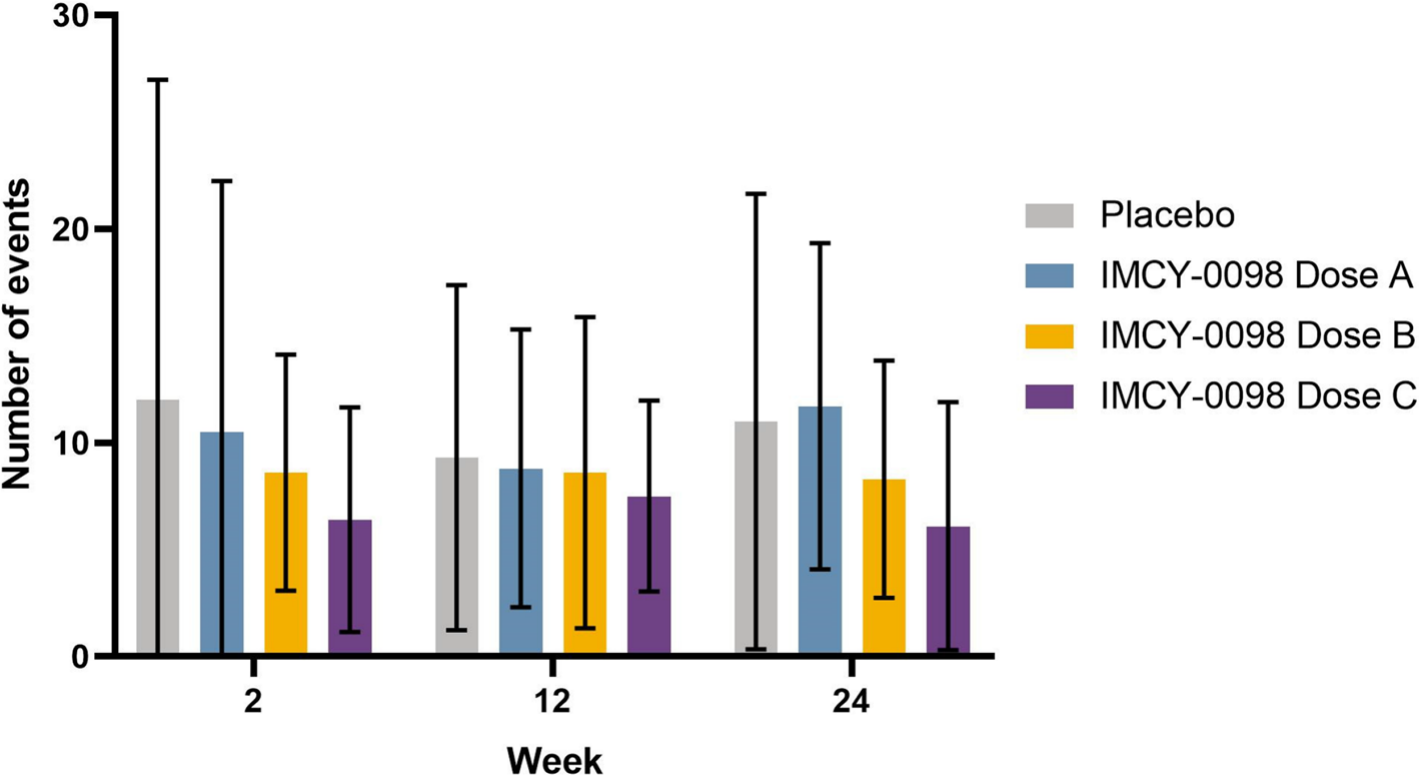
Mean total number of events of low glucose. Mean total number of low glucose events over the past 14 days. Data are shown for the intent-to-treat population. Error bars show standard deviation. Dose A: 50 μg at Week 0 followed by 3 × 25 μg; dose B: 150 μg at Week 0 followed by 3 × 75 μg; dose C: 450 μg at Week 0 followed by 3 × 225 μg. Events of low glucose were defined as being within the hypoglycaemic range (< 3.9 mmol/L or < 70 mg/dl or < 0.7 g/L)

Although no formal analysis was performed for the patient-reported glycemia data, no significant differences were observed for episodes of hypoglycemia (Fig. 5).

### Long-term follow-up

A total of 30 patients from the ITT population participated in the follow-up to Week 48: placebo (*n* = 7), IMCY-0098 dose A (*n* = 4), dose B (*n* = 7), or dose C (*n* = 12). Overall, 73% of patients rolled over to the long-term follow-up study in the placebo (70%), dose A (67%), dose B (78%), and dose C (75%) groups. A total of 24 AEs were reported and considered related to the study treatment (Additional file 2: Table S5). There were no SAEs. There were some small changes in clinical chemistry (Additional file 2: Table S6) and hematology parameters (Additional file 2: Table S7) from baseline to Week 36 and Week 48, but no consistent trends over time have been identified and no relevant differences were observed between the treatment groups. There were no clear changes in vital signs in any treatment group and no apparent differences were found between the groups (Additional file 2: Table S8). There were some small changes in ECG measurements from baseline to Week 48 but no consistent trends over time and no clear differences were found between groups (data not shown). Overall, no clinically significant differences were detected at Week 48 compared to baseline.

Mean fasting C-peptide was similar between the treatment groups at baseline and no notable changes were observed in any group. The mean (SD) change from baseline to Week 48 was − 0.02 (0.12) nmol/L in the placebo group, 0.02 (N/A) nmol/L in the dose A group, − 0.06 (0.17) nmol/L (SD:0.17) in the dose B group, and − 0.12 (0.15) nmol/L in the dose C group. There were no significant differences between groups.

## Discussion

Since the discovery of insulin therapy over 100 years ago, there have been no major changes in the clinical management of T1D apart from technical improvements in, for example, insulin variants, continuous glucose monitoring, and insulin pumps. These technologies clearly facilitate the daily control of blood glucose for patients but have had no impact on the underlying cause of the disease nor on the prevention of further disease progression. To date, there is still an important unmet need for disease-modifying therapies that would help patients with T1D achieve optimal glycemic targets, halt disease progression, and prevent β-cell loss. In this report, we have presented the first-in-human study of Imotope™ technology previously shown to eliminate pathogenic autoreactive T cells in an antigen- and disease-specific manner while preserving general immune system function in animal models.

The main focus of the study was safety. As T1D can be effectively controlled with insulin, clinical development of any novel treatments, including disease-modifying therapies, has to include a broad assessment of safety parameters to meet very stringent criteria before the treatment can enter clinical practice [34, 35]. IMCY-0098 demonstrated an encouraging safety profile up to 48 weeks at all doses and there were no major treatment-related safety issues throughout the study. Most AEs were transient and mild in nature; the only SAE considered as possibly related to study treatment was vestibular neuronitis in a patient who received the highest dose of IMCY-0098 (dose C), occurring 4 months after the last injection of study treatment. In addition, vestibular neuronitis is known to be associated with T1D; animal models of diabetes have shown pathophysiological changes in the peripheral vestibular apparatus and many potential mechanisms have been identified that may contribute to vestibular dysfunction in diabetes [36]. β-cells have been shown to express HLA class II molecules [37]; however, their capacity to act as APCs has been a matter of debate over the years with no conclusive evidence in either direction [38, 39]. If these cells do act as APCs, there would be a risk that treatment with IMCY-0098 could exacerbate T1D via direct attack of β-cells. It was therefore important to evaluate this risk during the study. The results show that in patients with recent-onset T1D treated with escalating doses of IMCY-0098, there was no evidence of disease progression. Furthermore, the overall decrease in C-peptide was lower than expected compared to a previous study of C-peptide levels following initial T1D diagnosis [31], except for in the low dose group where the decrease was as expected.

Overall, the lack of differences observed in different clinical parameters was expected due to the small sample size and short duration of follow-up among an adult population, who are known to show slower disease progression [31]. As this study is a first-in-human study, the dosing and treatment regimen may most likely be suboptimal and will need further exploration in future clinical studies. Notably, the clinical responses across all treatment arms (AUC C-peptide from MMTT, insulin consumption, HbA1c, and episodes of hypoglycemia) were within the expected range when compared with a previously published model [31].

Insulin therapy transformed the treatment of T1D, however, it does not modify the underlying cause of the disease or prevent complications [40]. Recently, therapies that specifically target underlying mechanisms of disease have shown efficacy in different settings. GAD65-alum antigen-specific therapy for the treatment of HLA DR3-associated β-cell destruction has shown a good efficacy and safety profile in clinical trials of newly diagnosed patients [41]. However, the intra-lymphatic delivery required needs high levels of expertise and may not be considered a patient-friendly approach. Subcutaneous delivery did not elicit the same immune response in a pilot trial of patients with T1D and was therefore not considered a suitable replacement [42, 43]. Teplizumab, an anti-CD3 antibody which binds the surface of T cells, has demonstrated the ability to delay stage 3 diagnoses in patients treated during stage 2 by an average of 2 year, and has recently been approved in this setting in the US [44]. Compared with GAD65-alum treatment, teplizumab is a broader immunosuppressive therapy that has some transient yet drastic effects on T cell populations, such as lymphopenia, as well as other adverse effects such as rash, cytokine release syndrome, and increased reactivation of Epstein-Barr virus among immunodeficient patients [44, 45]. Although such therapy may be beneficial for T1D patients, there is still concern regarding the use of such a broad immunosuppressive therapy, particularly in children. Therefore, the development of efficient therapies that combine antigen-specificity, ease of administration, and an excellent safety profile is still relevant in order to provide more options to patients with T1D.

This study has demonstrated an encouraging safety profile of IMCY-0098 for three dosages and with a dosing scheme including four administrations. Patients treated with IMCY-0098 dose C showed no deterioration and a tendency for a slight improvement in clinical parameters compared with the expected natural history of the disease, but the results from a larger, ongoing phase 2 study are needed to explore dosing regimens and make the final dosing decision. Future studies are also needed to fully elucidate the immune response and level of β-cell protection following treatment with IMCY-0098. As IMCY-0098 targets both DR3 and DR4, these HLA haplotypes being represented in 95% of the T1D population [46], it may be more broadly applicable in the treatment of T1D.

Autoantibody levels have been discussed in the context of assessing disease severity in T1D [47]. Here, we observed a downward trend in autoantibody levels in patients treated with IMCY-0098, suggesting no disease progression. Furthermore, based on the proposed mechanism of action of Imotopes™, there is a potential for cytolytic CD4^+^ T cells to destroy T-helper cells specific for β-cell autoantigens, leading to inhibition of autoantibody-producing B cells and a decreased production of autoantibodies. These preliminary results endorse investigation of autoantibody levels in future studies involving larger patient numbers.

Limitations of this study include the small sample size and short duration of the study. There were also imbalances in cohort composition in regards to DR3^+^/DR4^+^, as well as sex, which may reflect the differences in patterns of T1D onset over time between males and females [48]. There was also no stratification of cohorts which had to be considered when interpreting comparisons between groups.

## Conclusions

This first-in-human study showed that IMCY-0098 has a promising safety profile in patients with recent-onset T1D at all doses tested. The safety profile of IMCY-0098 supports further investigation and risk/benefit optimization for different doses and schedules of this novel treatment. This will be further evaluated in an ongoing phase 2 study (NCT: NCT04524949) with improved dosing and a higher sample size designed to provide clinical proof of concept of the Imotope™ technology as well as deeper characterization of the expected mechanism of action.

## Supplementary Information

**Additional file 1.** Study protocol.

**Additional file 2:** **Fig. S1.** Study visits and assessments. **Fig S2.** Autoantibody levels. **Table S1.** Adverse events to Week 24. **Table S2.** Vital signs to Week 24. **Table S3.** MMTT to Week 24. **Table S4.** Insulin dose to Week 24. **Table S5.** Adverse events Week 24-48. **Table S6. **Clinical chemistry parameters to Week 48. **Table S7.** Hematology parameters to Week 48. **Table S8.** Vital signs to Week 48.

## Data Availability

De-identified patient data can be provided to independent qualified researchers upon submission of a written application/research proposal that should be approved by the study sponsor, Imcyse S.A.

## Abbreviations

AE: Adverse event
ANCOVA: Analysis of covariance
APC: Antigen-presenting cell
AUC: Area under the curve
CI: Confidence interval
GAD65: Glutamic acid decarboxylase
HbA1c: Glycated hemoglobin
HLA: Human leukocyte antigen
IA-2: Insulinoma-associated antigen-2
ITT: Intent-to-treat
MHC: Major histocompatibility complex
MMTT: Mixed Meal Tolerance Test
SAE: Serious adverse event
T1D: Type 1 diabetes
ZnT8: Zinc transporter 8
