# Supplementary material for: First-in-human, double-blind, randomized phase 1b study of peptide immunotherapy IMCY-0098 in new-onset type 1 diabetes
Source: BMC Med. 2023 May 24;21:190. doi: 10.1186/s12916-023-02900-z (PMC10210318; doi:10.1186/s12916-023-02900-z)
Supplement: Supplementary file 1 — Additional file 1. Study protocol. [file 12916_2023_2900_MOESM1_ESM.pdf]

Imcyse SA
[redacted]

CLINICAL STUDY PROTOCOL

**Study products**	Imcyse IMCY-0098 combined with Alum adjuvant
**Study number and abbreviated title**	IMCY-T1D-001: Phase I study of IMCY-0098 in Recent Onset Type 1 Diabetes
**EudraCT number**	2016-003514-27
**Version**	2.0
**Version date**	[redacted]
**Title**	A PHASE I, PLACEBO CONTROLLED, DOUBLE-BLIND, DOSE ESCALATION CLINICAL TRIAL TO EVALUATE THE SAFETY AND IMMUNE RESPONSES OF IMCYSE's IMCY-0098 IN PATIENTS WITH RECENT ONSET TYPE 1 DIABETES
**Coordinating author**	[redacted]<br>Chief Executive Officer & Chief Medical Officer,<br>Imcyse SA
**Sponsor**	Imcyse SA, [redacted]

– CONFIDENTIAL –

This document and its contents are the property of and confidential to Imcyse. Any unauthorized copying or use of this document is prohibited.

Imcyse APPROVAL

______________________________ Date: ___________________________

[redacted]
(Chief Executive Officer & Chief Medical Officer)

INVESTIGATOR PROTOCOL AGREEMENT PAGE

Protocol Number and Abbreviated Title:	IMCY-T1D-001: Phase I study of IMCY-0098 in Recent Onset Type 1 Diabetes

I agree:

- To assume responsibility for the proper conduct of the study at this site.
- To conduct the study in compliance with this protocol, any mutually agreed future protocol amendments, and with any other study conduct procedures provided by Imcyse.
- To ensure that all persons assisting me with the study are adequately informed about the Imcyse investigational product and other study-related duties and functions as described in the protocol.
- Not to implement any changes to the protocol without agreement from Imcyse and prior review and written approval from the Institutional Review Board (IRB) or Independent Ethics Committee (IEC), except where necessary to eliminate an immediate hazard to the patients, or where permitted by all applicable regulatory requirements (for example, for administrative aspects of the study).
- That I am thoroughly familiar with the appropriate use of the study product, as described in this protocol, and any other information provided by Imcyse, including, but not limited to, the following: the current Investigator's Brochure (IB) or equivalent document, IB supplement (if applicable).
- That I am aware of, and will comply with, "Good Clinical Practice" (GCP) and all applicable regulatory requirements.
- That I have been informed that certain regulatory authorities require Imcyse to obtain and supply, as necessary, details about the Investigator's ownership interest in Imcyse or the investigational product, and more generally about his/her financial ties with Imcyse. Imcyse will use and disclose the information solely for the purpose of complying with regulatory requirements.

Hence I:

- Agree to supply Imcyse with any necessary information regarding ownership interest and financial ties (including those of my spouse and dependent children).
- Agree to promptly update this information if any relevant changes occur during the course of the study and for 1 year following completion of the study.
- Agree that Imcyse may disclose any information it has about such ownership interests and financial ties to regulatory authorities.
- Agree to provide Imcyse with an updated Curriculum Vitae.

Agreed by:

____

Investigator Name, Signature and Date

LIST OF CONTACTS

Sponsor:	Imcyse SA<br>[redacted]
Study Principal Investigator	[redacted]<br>Hôpital Cochin<br>[redacted]
Sponsor's Responsible Medical Director:	[redacted]<br>Imcyse SA<br>[redacted]
Study Manager:	[redacted]<br>Imcyse SA<br>[redacted]

1. STUDY SYNOPSIS

**Title of Study:**	A phase I placebo-controlled, double-blind, dose escalation clinical trial to evaluate the safety and immune responses of Imcyse's IMCY-0098 in patients with recent onset type 1 diabetes
**Protocol Number:**	IMCY-T1D-001
**Phase of Development:**	1
**Study Rationale:**	This clinical study will evaluate an innovative autoantigen-targeted immune-modulation therapy for the treatment of type 1 diabetes (T1D). This immunotherapeutic consists of one modified peptide that covers a well-characterized epitope from an auto-antigen, proinsulin, combined with an adjuvant system (alum) used in registered vaccines. It is expected to be disease-modifying by stopping the auto-immune-mediated destruction of islet β-cells in the pancreas through an antigen-specific mechanism of action whilst preserving overall immune competence of the patients.<br><br>At present, there is no cure or approved disease-modifying therapy available for T1D patients today. T1D is a chronic autoimmune disease, with circulating autoantibodies to islet autoantigens such as insulin, GAD65, IA-2 or ZnT8, in which insulin-producing β-cells located in the pancreatic islets of Langerhans are gradually destroyed by autoreactive T cells, involving CD4+ and CD8+ T lymphocytes. CD4+ T cells are believed to play a major role in the orchestration of the effector CD8+ T cells responsible for islet β-cell destruction. The pathophysiology of T1D with known islet cell autoantigens and T cell epitopes makes this disease a particularly attractive indication for development of an immunotherapeutic based on the Imcyse technology.<br><br>The platform technology of Imcyse makes use of short synthetic peptides encompassing epitopes recognised by CD4+ T lymphocytes flanked by a thioreductase motif. These cells are converted into cytolytic CD4+ T lymphocytes (cCD4) following interaction with antigen-presenting cells (APC) displaying both antigen and class II major histocompatibility complexes (MHC). These cCD4 are able to induce apoptosis of (1) the APC present in the draining lymph node of the pancreas and with which an immune synapse is formed and (2) diabetogenic T cells recognising the same or alternative epitopes on the same APC. The net result of such an interaction is to suppress the ongoing auto-immune response. Therefore, the Imcyse technology presents an opportunity to silence the autoimmune reaction at an early stage of the disease in an antigen-specific manner and may provide long term islet β-cell preservation.<br><br>To date, preclinical proof-of-concept (PoC) for T1D has been extensively demonstrated in Non-Obese Diabetic (NOD) mice, which are commonly used and recognized as a preclinical model of T1D.<br><br>The objectives of this phase I clinical trial in subjects recently diagnosed with T1D are: (1) to investigate safety features of Imcyse's IMCY-0098 (2) to further explore and document in humans the immune mechanism of action particularly through the induction of cCD4 T cells associated with expected β-cell preservation activity, and (3) to find a dose or dose range to be carried forward in clinical studies.

**Objectives:**	**Primary Objective**<br><br>The primary objective of this study is to assess, in adults with recent onset T1D, the safety of IMCY-0098 at three different doses and of placebo.<br><br>**Secondary Objective**<br><br>The secondary objective of this study is to evaluate the clinical response to IMCY-0098 by assessing disease activity.<br><br>**Exploratory Objectives**<br><br>• To evaluate and characterize the proinsulin-specific CD4+ T cells induced by IMCY-0098<br>• To evaluate the impact of IMCY-0098 on autoreactive T-cell responses specific for autoantigens expressed by islet β-cells (proinsulin, GAD65, IGRP)<br>• To evaluate the impact of IMCY-0098 on autoantibodies against GAD65, IA-2, ZnT8 and insulin
**Study Design:**	This is a phase 1, randomized, double-blind, dose escalation, placebo-controlled, multicenter clinical trial.<br><br>For each patient, the study comprises a total of 7 visits occurring over a period of approximately 24 weeks (from the first administration of study product to the last planned visit). The patients will undergo planned assessments and procedures as outlined in the table of study procedures.<br><br>Study patients will be sequentially enrolled into one of the three cohorts and allocated to receive IMCY-0098 or placebo in a 3:1 randomization ratio, as outlined below:<br><br>• Cohort 1: One administration of IMCY-0098, 50 micrograms (µg) combined with alum adjuvant, followed by 3 administrations of IMCY-0098 25 µg combined with alum adjuvant OR four administrations of matching placebo with alum adjuvant<br>• Cohort 2: One administration of IMCY-0098, 150 µg combined with alum adjuvant, followed by 3 administrations of IMCY-0098 75 µg combined with alum adjuvant OR four administrations of matching placebo with alum adjuvant<br>• Cohort 3: One administration of IMCY-0098, 450 µg combined with alum adjuvant, followed by 3 administrations of IMCY-0098 225 µg combined with alum adjuvant OR four administrations of matching placebo with alum adjuvant<br><br>A total of 40 patients (6 active, 2 placebo patients in Cohort 1, 9 active, 3 placebo patients in Cohort 2, and 15 active, 5 placebo patients in Cohort 3) are planned to be enrolled.<br><br>In each cohort, the first 4 patients will stay in the hospital for a period of 24 hours to allow close safety follow-up after each of the four administrations.<br><br>In each cohort, an interval of 2 days between each of the first 4 patients will be respected at each administration. Patients subsequently enrolled at the same dose (4 in Cohort 1, 8 in Cohort 2, and 16 in Cohort 3) will be included without limitation in time.<br><br>When 4 patients in Cohort 1 have received their first administration AND at least 3 patients have received all 4 administrations (week 6 + 2 days), a safety evaluation will be conducted by an independent Data and Safety

	Monitoring Board (DSMB) to allow inclusion of patients in Cohort 2 once Cohort 1 has been fully recruited.<br><br>When 4 patients in Cohort 2 have received their first administration AND at least 3 patients have received all 4 administrations, a safety evaluation will be conducted by the DSMB to allow inclusion of patients in Cohort 3 once Cohort 2 has been fully recruited.<br><br>If the DSMB recommends not to enroll patients at a higher dose and if there is no recommendation to stop the study, additional patients may be enrolled in currently recruiting or previous cohorts.<br><br>Should one of the pre-determined events occur, an ad hoc DSMB meeting will be organized and recruitment of patients will be temporarily halted. Treatment of patients already enrolled will be stopped and patients will be followed-up according to the protocol Patients who have been already fully treated (4 administrations completed) will also be followed-up according to the protocol. Recommendations of the DSMB will be followed for either restart (under DSMB defined conditions) or premature end of study.<br><br>The pre-determined events which will trigger an ad hoc DSMB meeting are:<br>• Investigator request<br>• Suspected Unexpected Serious Adverse Reaction (SUSAR)<br>• Anaphylactic shock<br><br>The safety profile of the different doses will be based on the following **safety considerations**:<br>• Occurrence, intensity and relationship of any solicited local and general adverse event (AE) during a 7-day follow-up period (i.e. day of study drug administration and 6 subsequent days) after each IMCY-0098 or placebo dose<br>• Occurrence, intensity and relationship of unsolicited local and general AEs occurring throughout the study period<br>• Occurrence and relationship of all serious adverse events (SAEs) occurring throughout the study period<br>• Occurrence, intensity and relationship of any abnormality in physical examination, vital signs, 12-lead ECG<br>• Hematological and biochemical levels within or outside the normal ranges<br>• Evolution of C-peptide concentration over the 6 months duration of the study.<br><br>This safety analysis should support the selection of a dose level to be used in future clinical trials.<br><br>**Planned analyses**<br><br>As described above, an analysis and review of blinded safety data will be performed prior to enrollment of patients into Cohort 2 and Cohort 3, respectively.<br><br>The final analysis will be performed when all subjects will have completed the Week 24 visit.<br><br>**Data Safety Monitoring Board (DSMB)**<br><br>A DSMB will oversee the conduct of the study and ensure the safety of participating patients. The role and responsibilities of the DSMB will be outlined in detail in a DSMB charter.

**Eligibility Criteria:**	**Target population**: Adult patients with recently diagnosed T1D with residual β cell function<br><br>**<u>Inclusion Criteria</u>**<br><br>1. Male or female 18 to 30 years of age<br>2. Initial diagnosis of Type 1 diabetes according to ADA/WHO criteria within the past 6 months<br>3. Insulin requirement, as determined by the investigator<br>4. Presence of at least one autoantibody (GAD65, IA-2, or ZnT8)<br>5. Fasting C-peptide at screening > 0.2 nmol/L and/or stimulated C-peptide ≥ 0,4 nmol/L.<br>6. HLADR3-positive and/or HLADR4-positive<br>7. Willingness to undergo the insulin treatment prescribed by the physician<br>8. Body mass index (BMI) between 17–28 kg/m^2 at screening<br>9. Fully informed written consent obtained<br>10. Males with reproductive potential should use barrier method of contraception (condom) from screening up to 90 days after last treatment with investigational product.<br>11. Women of childbearing potential should use an highly effective contraception method from screening and for the whole duration of the study.<br><br>*Of child-bearing potential is defined as being post onset of menarche and not meeting any of the following conditions:*<br>*- menopausal for at least 2 years,*<br>*- having undergone bilateral tubal ligation at least 1 year previously*<br>*- having undergone bilateral oophorectomy or hysterectomy.*<br>*HIGHLY EFFECTIVE contraceptive measures acceptable for the whole duration of the study have been defined based on the CTFGs recommendations on contraception and are the following:*<br>*- Combined (estrogen and progestogen containing) hormonal contraception associated with inhibition of ovulation (oral, intravaginal, transdermal),*<br>*- Progestogen-only hormonal contraception associated with inhibition of ovulation (oral, injectable, implantable).*<br>*- Intrauterine device (IUD)*<br>*- intrauterine hormone-releasing system (IUS)*<br>*- Monogamous relationship with vasectomized partner. Partner must have been vasectomized for at least 6 months prior to the patient's entry into the study*<br>*- Abstinence or absence of sexual relations with men.*<br><br>**<u>Exclusion criteria</u>**<br><br>1. Ongoing or planned pregnancy during the whole duration of the study or lactation<br>2. Presence of significant medical conditions in particular chronic liver condition, chronic hematological disease, renal dysfunction of grade 2 or more according to the World Health Organization (WHO) Toxicity Scale .<br>3. Has any current signs or symptoms of infection at entry or within 2 weeks of entry or has received intravenous antibiotics within 2

	months prior to the first planned administration of the study product<br>4. Has received any live, attenuated vaccine within 3 months prior to the first planned administration of the study product (i.e. oral poliomyelitis vaccine, measles-mumps-rubella vaccine, yellow fever vaccine, Japanese encephalitis vaccine, dengue vaccine, rotavirus vaccine, varicella vaccine, live-attenuated zoster vaccine, Bacillus Calmette-Guérin [BCG] vaccine, oral typhoid vaccine)<br>5. History of, or current malignancy (except excised basal cell skin cancer)<br>6. Clinical evidence of a diabetes-related complication that could interfere with patient's participation/completion of study<br>7. Primary or secondary immune deficiency disorders<br>8. Human Immunodeficiency virus (HIV), chronic hepatitis B virus (HBV) or hepatitis C virus (HCV) infection<br>9. Presence at screening of abnormal laboratory values grade 2 or more according to the World Health Organization (WHO) Toxicity Scale<br>10. Anti-diabetic treatments other than insulin in the week prior to first study drug administration<br>11. Ongoing treatment with immunosuppressive agents or treatment within the past year with the exception of topical or intra nasal corticosteroids.<br>12. Treatment with immunotherapy within the past 3 months<br>13. Treatment with an investigational drug within the past 3 months.<br>14. Patients with a known hypersensitivity to any component of the drug product should be excluded from the study<br>15. Patients under treatment with statins at the time of screening.
**Investigational Therapy:**	The investigational medicinal product (IMP) consists in a small synthetic peptide (20 amino acids – IMCY-0098) combining a known human epitope of proinsulin flanked with a thioredox motif, presented in the form of a powder and solvent for subcutaneous (SC) administration. The solvent includes the adjuvant aluminum hydroxide (alum) at a concentration of 500 µg/mL.<br><br>Treatment will consist of 4 immunizations (separated by 14 days) of the IMP or the placebo by sc. Half of the dose to be administered will be injected concomitantly in two sites (the upper arm, in the region of the lateral part of the arm, midway between the elbow and the shoulder).<br><br>The low dose (Cohort 1) will consist of the SC administration of 50 µg of peptide in two separate injections of 25 µg each (100 µL each) followed by three consecutive injections of 25 µg of peptide in two separate injections of 12.5 µg each (50 µL each).<br><br>The medium dose (Cohort 2) will consist of the SC administration of 150 µg of peptide in two separate injections of 75 µg each (300µL each) followed by three consecutive injections of 75 µg of peptide in two separate injection of 37.5 µg each (150 µL each).

	The higher dose (Cohort 3) will consist of the SC administration of 450 µg of peptide in two separate injection of 225 µg each (900µL each) followed by three consecutive injections of 225 µg of peptide in two separate injection of 112.5 µg each (450 µL each).<br>A single batch of IMP will be used for the whole study.
**Treatment Duration:**	The study duration will be 24 weeks for each patient enrolled.<br>Each patient will receive four administrations of study drug or placebo, with 2 weeks interval between each administration.
**Mandatory Concomitant Therapy**	Insulin: all patients should be treated according to an optimized conventional treatment with 1-2 injections of slow acting insulin per day and one dose of fast acting insulin prior to each meal.
**Prohibited Concomitant Therapies**	• Anti-diabetic drugs other than insulin should be avoided during the study<br>• Immunosuppressive therapies, with the exception of topical or intra nasal corticosteroids<br>• Live, attenuated vaccines<br>• Statins<br>• Investigational or non-registered pharmaceutical or biological product other than the study products
**Study Endpoints:**	**Primary Safety Endpoints**<br>• Occurrence, intensity and relationship of any solicited injection site and systemic AEs during a 7-day follow-up period (*i.e.*, day of study product administration and 6 subsequent days) after each IMCY-0098 or placebo dose<br>• Occurrence, intensity and relationship of any unsolicited injection site and systemic AEs throughout the study period<br>• Occurrence and relationship of all serious adverse events (SAEs) throughout the study period<br>• Occurrence, intensity and relationship of any abnormality in physical examination, vital signs, 12-lead ECG<br>• Hematological and biochemical levels outside the normal ranges<br>• C-peptide concentrations throughout the study period<br><br>**Secondary Efficacy Endpoints**<br>• Post challenge C-peptide (2 hr AUC of MMTT)<br>• Fasting C-peptide<br>• HbA1c<br>• Insulin dose<br>• Glycemic profile as measured by the patient through the use of the Freestyle Libre system.<br><br>**Exploratory Immunogenicity Endpoints**<br>• Suppression of CD4+/CD8+ effector responses by CD4+ T cells specific for IMCY-0098 peptide<br>• CD4+ T cell response specific for IMCY-0098 peptide<br>• CD4+ and CD8+ T cell responses specific for insulin, GAD65 and IGRP<br>• Change in diabetes auto-antibody levels (GAD65, IA-2, ZnT8, insulin)

	Correlations between immunogenicity responses, clinical and/or biological parameters will be performed on an exploratory basis.
**Statistical Design and Sample Size:**	The study sample size has been fixed without statistical power consideration, but is estimated as adequate to provide a reliable safety assessment of the tested doses, to provide a first information on the safety and preliminary efficacy/immunogenicity dose-response, and to obtain preliminary efficacy/immunogenicity assessments to inform the next steps of the clinical development.<br>All primary, secondary and exploratory endpoints will be summarized by descriptive statistics (continuous variables) or frequency tables (categorical variables), by dose group and overall.<br>If deemed relevant from the results of the descriptive analyses, exploratory statistical analyses or modeling will be performed to further characterize the efficacy dose-response.

Table 1 Table of study procedures

**Visit**	**1**	**2**	**3**	**4**	**5**	**6**	**7**	**8**
**Study week**	**Week -4 to 0**	**Week 0**	**Week 2**	**Week 4**	**Week 6**	**Week 12**	**Week 18**	**Week 24**
**Study product administration**		**1**	**2**	**3**	**4**			
Informed consent	●							
Collection of demographic data	●							
Evaluation of eligibility	●	●						
T1D specific and general medical history	●							
Recording of prior and current medication	●							
Record current insulin doses	●	●	●	●	●	●	●	●
Complete physical examination[1]	●	●	●	●	●	●	●	●
Vital signs[2]	●	●[8]	●[8]	●[8]	●[8]	●	●	●
ECG (12-lead)[12]	●	●[12]	●[12]	●[12]	●[12]			●
Serum pregnancy test[3]	●							
Urine pregnancy test[3]		●	●	●	●			
Urinalysis including dipstick and microscopic examination	●	●				●		●
Virology: anti-HIV, anti-HCV, HBsAg/anti-HBc	●							
Check contraindications		●	●	●	●			
Treatment allocation by IWRS		●	●	●	●			
Administer study product		●	●	●	●			
**Clinical assessments**								
Hematology and biochemistry[4]	●	●[8]	●[8]	●[8]	●[8]	●	●	●
Fasting C peptide[4]	●	●	●	●	●	●	●	●
Perform MMTT	●					●		●
HBA1c[4]		●				●		●
Glycemia[4]	●	●[8]	●[8]	●[8]	●[8]	●	●	●
Patient-reported Glycemia[11]		●	●	●	●	●	●	●
**Immuno assessments**								
Diabetes auto-antibodies[9]	●					●		●
HLA class I / class II genotype determination[9]	●							
Immuno-regulatory CD4+ T cell response[4, 10]		●			●	●		●
Effector CD4+/CD8+ T cell reponses[4, 10]		●			●	●		●

Visit	1	2	3	4	5	6	7	8
**Study week**	**Week -4 to 0**	**Week 0**	**Week 2**	**Week 4**	**Week 6**	**Week 12**	**Week 18**	**Week 24**
**Safety assessments**								
Record concomitant medication/medical conditions		●	●	●	●	●	●	●
Record injection site reactions one-hour post study product administration[5]		●[8]	●[8]	●[8]	●[8]			
Record solicited systemic AEs one-hour post study product administration[6]		●[8]	●[8]	●[8]	●[8]			
Provision of diary card for daily recording of solicited AEs by the patient[7]		●	●	●	●			
Return, checking and transcription of diary card			●	●	●	●		
Recording of non-serious and serious AEs	●	●[8]	●[8]	●[8]	●[8]	●	●	●
Study conclusion								●

[1] Including body temperature, weight and height; height being measured only during screening.
[2] Vital signs include brachial pulse and blood pressure. They will be performed after patient has been in a supine position for 3 minutes. At Visits 2, 3, 4, 5, vital signs will be performed prior to and 1 hour post study product administration.
[3] Serum and urine pregnancy tests for female patients of childbearing potential only.
[4] The blood samples are to be taken prior to the planned administrations of the study drug at that visit, when applicable
[5] Injection site reactions will be recorded by study staff 1 hour post study product administration by asking the patient about his/her perception of pain/tenderness and itching sensation and via visual assessment of erythema/redness, inflammation/swelling and induration
[6.] Solicited systemic AEs will be recorded by study staff one hour post study product administration, in particular headache, fatigue, malaise and myalgia. Temperature will also be recorded.
[7] Daily recording by patients of the presence or absence and severity of solicited local reactions and systemic adverse events on the day of the administration and the six subsequent days (i.e., 7 days in total)
[8] For the first 4 patients in each cohort, this assessment will be repeated every 6h during the 24h hospitalization at these visits. Vitals signs will include measure of the body temperature. Pre-dose blood sampling for hematology and biochemistry analysis will be processed through the central laboratory. Hematology and biochemistry analysis at 6h, 12h, 18h and 24h post dose will be done locally and will include glucose measurement. (S)AEs will be recorded throughout the 24h hospital stay.
[9] Samples for autoantibodies detection and HLA genotyping will be processed through the central laboratory used for Hematology and Biochemistry analysis.
[10] These blood samples will be processed through a lab specialized in immunology, different from the central lab used for Hematology and Biochemistry.
[11] At visit 2, patient will receive the FreeStyle Libre Flash Glucose scanner and the first sensor will be applied. At each subsequent visit, data from the previous period will be downloaded from the scanner and new sensor(s) will be delivered to cover the period until the next study visit.
[12] An ECG must be performed and reported for any of the first 4 patients if the vital signs results are abnormal at any time point during the 24h hospitalization period.

2. TABLE OF CONTENTS

PAGE

1. STUDY SYNOPSIS......5

2. TABLE OF CONTENTS......14

LIST OF TABLES......18

LIST OF FIGURES......18

LIST OF APPENDICES......18

3. LIST OF ABBREVIATIONS......19

4. BACKGROUND AND RATIONALE......21

4.1 Introduction......21
4.2 Therapeutic Strategy......22
4.3 Imcyse IMCY-0098......22
4.3.1 Product information......22
4.3.2 Preclinical experience......23
4.3.3 Justification of doses, safety margin and treatment schedule......23
4.3.4 Previous human experience......25
4.4 Summary of Known and Potential Risks and Benefits to Humans......25

5. STUDY OBJECTIVES......25

5.1 Primary Objective......25
5.2 Secondary Objective......25
5.3 Exploratory Objectives......25

6. INVESTIGATIONAL PLAN......26

6.1 Description of Overall Study Design and Plan......26
6.1.1 Study design......26
6.1.2 Planned analyses......29
6.2 Rationale and Discussion of Study Design......29

7. STUDY POPULATION......29

7.1 Number of Subjects......29
7.2 Recruitment Methods......30
7.3 Eligibility Criteria......30
7.3.1 Inclusion criteria......30
7.3.2 Exclusion criteria......31

8. INVESTIGATIONAL PRODUCT AND ADMINISTRATION 32
8.1 Study Products 32
8.1.1 IMCY-0098 32
8.1.2 Placebo 32
8.2 Dosage and Administration 33
8.3 Packaging and Labelling 34
8.4 Storage and Return 34
8.5 Treatment Allocation and Randomization 34
8.5.1 HLA A2 genotype and randomization 34

9. STUDY PROCEDURES 35
9.1 Subject Identification 35
9.2 Method of Blinding and Breaking the Study Blind 35
9.3 Independent Data and Safety Monitoring Board 36
9.4 Treatment Modification and Discontinuation Criteria 36
9.4.1 Study hold 36
9.4.2 Subject withdrawal from the study 36
9.4.3 Contraindications to administration of subsequent doses of study product 37
9.5 Concomitant Medications 38
9.5.1 Mandatory medications during the study 38
9.5.2 Prohibited medications during the study 38
9.6 Detailed Description of Study Procedures 39
9.6.1 Visit 1 (Screening) 40
9.6.2 Visit 2 (Study Day 1) = baseline 41
9.6.3 Visit 3 (Week 2), Visit 4 (Week 4) and Visit 5 (Week 6) 42
9.6.4 Visit 6 (Week 12) 43
9.6.5 Visit 7 (Week 18) 43
9.6.6 Visit 8 (Week 24) = End-of-study visit or Premature Discontinuation 44
9.6.7 End of trial 45

10. DESCRIPTION OF ASSESSMENTS 45
10.1 Informed Consent 45
10.2 Administration of Study Product 45
10.3 Safety Assessments 45
10.3.1 Medical history 45
10.3.2 Physical examination and vital signs 46
10.3.3 Recording of concomitant medications/medical conditions 46
10.3.4 Recording of adverse events and serious adverse events 46
10.3.5 Pregnancy and Contraception 46

10.3.6 24 hour hospitalization for the first 4 patients in each cohort 47
10.4 Laboratory Assessments 48
10.4.1 Sample handling and analysis 48
10.4.2 Sample volumes and sample handling 48
10.4.3 Laboratory parameters and assays 48
10.5 Clinical Assessments 51
10.5.1 Complete physical examination and temperature measurement 51
10.5.2 Vital signs 51
10.5.3 12-lead electrocardiogram 51
10.5.4 Mixed-meal tolerance test (MMTT) 52
10.5.5 Glycemic profile 54

11. ADVERSE EVENTS AND SERIOUS ADVERSE EVENTS 54
11.1 Definition of an Adverse Event 55
11.2 Assessment of Adverse Event Intensity 55
11.2.1 Injection site reactions 55
11.2.2 Systemic solicited adverse events 56
11.2.3 Unsolicited adverse events 57
11.3 Assessment of Adverse Event Outcome 58
11.4 Assessment of the Relationship to Study Products 58
11.5 Serious adverse events 59
11.6 Adverse Event Recording 60
11.7 Adverse Event and Pregnancy Reporting 60
11.7.1 Adverse event reporting 60
11.7.2 Pregnancy reporting 61
11.8 Emergency Medication 61

12. STATISTICAL ANALYSES 61
12.1 Determination of Sample Size 61
12.2 Primary Safety Endpoints 61
12.3 Secondary Efficacy Endpoints 62
12.4 Exploratory immunogenicity endpoints 62
12.5 Analysis Populations 62
12.6 Statistical Considerations 62
12.7 Statistical Methods 63
12.7.1 Subject disposition 63
12.7.2 Protocol deviations 63
12.7.3 Demographic, baseline and follow-up characteristics 63
12.7.4 Analysis of the primary safety endpoints 63
12.7.5 Analysis of the secondary efficacy endpoints 64
12.7.6 Analysis of the exploratory immunogenicity endpoints 64

13. STUDY MANAGEMENT 65
13.1 Regulatory Guidelines 65
13.2 Independent Ethic Committees 65
13.3 Informed Consent 65
13.4 Insurance and Indemnity 66
13.5 Protocol Adherence - Amendments 66
13.6 Compliance Control and Monitoring 66
13.6.1 Patient compliance 66
13.6.2 Site compliance and monitoring 66
13.6.3 Medical monitor 67
13.6.4 Case report forms and data entry 67
13.7 Reporting and Communication of Results 67
13.8 Quality Assurance 68
13.9 Retention of Records 68
13.10 Patient Data Protection 68
13.11 Confidentiality 69
13.12 Publication 69

14. REFERENCES 71

LIST OF TABLES

PAGE

Table 1 Table of study procedures 12

Table 2 Exploratory objectives 26

Table 3 Time window between study visits 39

Table 4 Laboratory parameters and laboratories 48

Table 5 Intensity scale for injection site reactions 56

Table 6 Intensity scale for systemic solicited adverse events 57

LIST OF FIGURES

PAGE

Figure 1 IMCY-T1D-001 study design 27

LIST OF APPENDICES

PAGE

APPENDIX 1 72

APPENDIX 2: MODIFIED WHO TOXICITY SCALE 79

3. LIST OF ABBREVIATIONS

**γGT**	Gamma glutamyl transferase
**ADL**	Activities of Daily Living
**AE**	Adverse event
**ALP**	Alkaline phosphatase
**ALT**	Alanine aminotransferase
**APC**	Antigen-presenting cell
**AST**	Aspartate aminotransferase
**BMI**	Body mass index
**CA**	Competent Authorities
**cCD4**	Cytolytic CD4+ T cells
**cGMP**	current Good Manufacturing Practice
**CPK**	Creatine phosphokinase
**CRF**	Case Report Form
**DSMB**	Data and Safety Monitoring Board
**ECG**	Electrocardiogram
**GAD**	Glutamic acid decarboxylase
**GAD65**	Glutamic acid decarboxylase 65
**GCP**	Good Clinical Practice
**HBV**	Hepatitis B virus
**HCG**	β- Human chorionic gonadotropin
**HCV**	Hepatitis C virus
**HIV**	Human immunodeficiency virus
**IA-2**	Islet antigen 2
**IB**	Investigator Brochure
**ICF**	Informed consent form
**ICH**	International Conference on Harmonisation of Technical Requirements for Registration of Pharmaceuticals for Human Use
**IEC**	Independent Ethics Committee
**IGRP**	Islet-specific glucose-6-phosphatase catalytic subunit-related protein
**IMP**	Investigational medicinal product
**ITT**	Intent-to-treat
**IWRS**	Interactive Web Response System
**LDH**	Lactate dehydrogenase
**µg**	Microgram
**µL**	Microliter
**MedDRA**	Medical Dictionary for Regulatory Activities
**MHC**	Major histocompatibility complexes
**NCI**	National Cancer Institute
**NOD**	Non-Obese Diabetic
**PoC**	Proof-of-concept
**PP**	Per-protocol
**RBC**	Red blood cell
**SAE**	Serious adverse event
**SAF**	Safety

**SAP**	Statistical Analysis Plan
**SC**	Subcutaneous
**SOP**	Standard Operating Procedure
**SUSAR**	Suspected unexpected serious adverse reaction
**T1D**	Type 1 diabetes
**TEAE**	Treatment emergent adverse events
**TPN**	Total parenteral nutrition
**WBC**	White blood cell
**WMA**	World Medical Association
**ZnT8**	Zinc transporter 8

4. BACKGROUND AND RATIONALE

4.1 Introduction

Type 1 diabetes (T1D) is a hormonal deficiency disorder, in which there is loss of insulin secretion due to autoimmune destruction of the β-cells located in the pancreatic islets of Langerhans. Patients develop hyperglycemia that clinically manifests with polyuria, polydipsia and weight loss. Long-term complications of T1D can be life-threatening and include cardiovascular disease (*e.g.*, coronary artery disease, atherosclerosis and hypertension), neuropathy, nephropathy, retinopathy and an increased susceptibility to bacterial and fungal infections.

At present, there is no cure or disease-modifying therapy available for T1D patients. The mainstay of treatment is the subcutaneous administration of insulin on a daily basis. However even with treatment, the need for frequent self-monitoring of plasma glucose levels and the obligation to plan routine activities such as eating and exercising, make living with T1D problematic.

Unlike most autoimmune diseases, T1D is more common in males than females. The incidence of T1D peaks at 5 to 7 years of age and at puberty; symptoms of T1D can also appear in adulthood. The prevalence of T1D is highest in the age ranges of 35–60 years. The incidence rate varies significantly by geographical region. The highest incidence of T1D (age-adjusted rate of > 20/100,000 patient years) is observed in the Nordic countries, United Kingdom, and Sardinia, whereas China and South America have the lowest incidence of T1D (< 1/100,000 patient years) [as reviewed in Simmons and Michels, 2015]. The annual incidence of T1D is increasing globally by 2.3% per year.

Autoantibodies to islet antigens such as insulin, GAD65, IA-2 or ZnT8 can be measured in the serum of individuals at risk for T1D long before the onset of clinical symptoms. Indeed, the immunological diagnosis of T1D is presently based upon the detection of autoantibodies in the serum. T cell responses to islet autoantigens involving CD4+ and CD8+ T lymphocytes and leading to the destruction of β-cells by autoreactive T cells can also be detected [Roep ,and Peakman, 2012]. CD4+ T cells are believed to play a major role in the orchestration of the effector CD8+ T cells responsible for islet β-cell destruction.

4.2 Therapeutic Strategy

The pathophysiology of T1D with known islet cell autoantigens and T cell epitopes makes this disease a particularly attractive indication for development of an immunotherapeutic based on the Imcyse technology.

The platform technology of Imcyse makes use of short synthetic peptides encompassing epitopes recognised by CD4+ T lymphocytes flanked by a thioreductase motif. These cells are converted into cytolytic CD4+ T lymphocytes (cCD4) following interaction with antigen-presenting cells (APC) displaying both antigen and class II major histocompatibility complexes (MHC). These cCD4 are able to induce apoptosis of (1) the APC present in the draining lymph node of the pancreas and with which an immune synapse is formed and (2) diabetogenic T cells recognising the same or alternative epitopes on the same APC. The net result of such an interaction is to suppress the ongoing auto-immune response. Therefore, the Imcyse technology presents an opportunity to silence the autoimmune reaction at an early stage of the disease in an antigen-specific manner and may provide long term islet β-cell preservation.

4.3 Imcyse IMCY-0098

4.3.1 Product information

The investigational medicinal product (IMP) consists in a small synthetic peptide (20 amino acids – IMCY-0098) that contains a known human epitope from proinsulin flanked with a thioredox motif presented in the form of a powder and solvent for subcutaneous (sc) administration. The solvent includes the adjuvant aluminum hydroxide (alum).

Administration of synthetic peptides with no adjuvant would result in a lack of APC activation. In addition, it is mandatory to elicit immunogenicity in an environment which is maintained as physiological as possible, without triggering undue inflammatory conditions. This is required, as the mechanism of action follows a natural pathway by which CD4+ T cells are activated. For these reasons, aluminum hydroxide was chosen, as it results in the elaboration of a pathway used spontaneously by the body when dealing with mild stress conditions. Aluminum hydroxide has been used over the last 60 years in clinical vaccination and is reported to be safe [reviewed in Lindblad, 2004].

After reconstitution, the concentration of IMCY-0098 will be 250 micrograms (µg) per mL. Treatment will consist of 4 immunizations (separated by 14 days) of the IMP or placebo (see Section 8.2).

4.3.2 Preclinical experience

Imcyse has performed a number of studies in animal models to generate proof of concept (PoC) data validating a therapeutic approach in different pathologies (*e.g.*, see Carlier *et al.*, 2012; Malek Abrahimians *et al.*, 2015).

Preclinical PoC for T1D has been extensively demonstrated in Non-Obese Diabetic (NOD) mice, which are commonly used and recognised as a preclinical model of T1D (Kikutani and Makino, 1992).

Given the potential of the IMP for preventing and suppressing an auto-immune response, immune safety experiments have also been conducted. It has been shown that class II-restricted responses towards an unrelated antigen (*e.g.,* ovalbumin) were fully maintained in animals actively vaccinated with peptides containing epitopes and an oxido-reductase motif. The experiments were extended towards T cell independent immune responses and class I-restricted responses. Data obtained to date indicate that alternative aspects of the immune system integrity are maintained.

The risk of observing cytolytic cells as elicited by the technology reverting to an effector memory phenotype, with potential pathogenic consequences, was evaluated with both mouse and human CD4+ T cells. No concern was raised as, in culture, and under very stringent conditions forcing cells into polarization, it was not possible to revert the phenotype.

Please refer to the current Investigator Brochure (IB) for detailed information regarding the preclinical studies.

4.3.3 Justification of doses, safety margin and treatment schedule

- The maximal human dose (450µg) used for priming has been selected in the classical range of peptide treatment in clinical trials (Candia 2016).

  There is indication in the literature that priming with a full dose and boosting with lower amount of antigen can optimize the immune response. Indeed, for the RTS,S Malaria vaccine for which protective efficacy is known to be associated with specific CD4 T cells (Lumsden et al 2011), boosting with a lower dose induces better protection than with dose equivalent to priming dose (Regulates et al 2016). Therefore the boosting doses have been set at half of the priming dose.

- Regarding the toxicity, IMCY-0098 has undergone a GLP repeated dose and local tolerance study in mice. Results showed that 5 SC administrations of 225µg of the product did not induce safety concerns in mice. In this context, mice (25g) received a dose of 9000 µg/kg while the maximal human dose is 6.4 µg/kg for a subject of 70 Kg. Therefore, the relative safety margin is 1400 times in the case of a full human dose and 2800 in the case of a half full human dose . In addition, one more injection (n=5) was made in animals than in man.

  Therefore, based on the results of the GLP toxicity study and on the safety margin, no safety concerns are expected in humans. More details on GLP toxicity studies can be found in the current Investigator's Brochure.

- For the first 4 patients at each dose, a minimum interval of 48h between two patients will be respected at each injection timepoint. Although rare, acute and severe allergic reactions, such as anaphylactic reactions can occur following injection of a vaccine. Such events, when they declare, appear very quickly and within the first 24 hours following injection of a vaccine. This minimum interval of 48h has therefore been included to allow detection and management in one single subject of these potential acute severe side-effects.

- This protocol is designed to promote amplification and development of memory CD4 T cells with cytolytic potential and to limit the induction of terminally differentiated effector T cells and IMCY-0098 specific regulatory T cells which would reduce the functionality of the cytolytic CD4 T cells.

  Experiments conducted in mice indicate that four to five dosing were able to induce functional responses (memory phenotype with potent cytolytic properties and effector memory characteristics) which is in line with classical vaccines for which multiple dosing are required to induce the expected response. Therefore, a four injections' schedule has been selected for the Phase 1 trial.

- Finally, the schedule (1 injection every 2 weeks) has been selected to minimize the risk of induction of anergy/tolerance often observed following repeated treatment with short interval (e.g. 1 week) and favour the amplification and differentiation of memory cytolytic CD4+ T cells.

  Longer interval has not been selected to initiate this Phase 1 trial to reduce the time of the treatment and allow more rapid potential clinical benefit to the

selected patients (T1D, recently diagnosed) characterized by a fast evolution of their immune compartments leading to worsened pathology.

4.3.4 Previous human experience

To date, there is no experience in human subjects with IMCY-0098.

4.4 Summary of Known and Potential Risks and Benefits to Humans

Known and potential risks are those related to any SC injection (*i.e.*, erythema/redness, induration and swelling following injection). In addition, similarly to classical vaccines, local reactions can include pain/tenderness and itching. Systemic reactions, such as malaise, headache, and/or fever, can also occur. These events tend to be mild to moderate and transient. Rarely, acute and severe allergic reactions, such as anaphylactic reactions can occur following injection of a vaccine.

Since the immunomodulating effects of the Imotope™ are long-lasting in animal models, deleterious immunosuppression could also be long lasting. This is unlikely as all experiments in animal models have not shown any immunosuppressive effect.

Potential benefits could be a slowing down of the destruction of beta cells of the pancreas with a preservation of some insulin secretion hence a slower evolution of the disease worsening towards fully insulin dependent diabetes.

5. STUDY OBJECTIVES

5.1 Primary Objective

The primary objective of this study is to assess, in adults with recent onset T1D, the safety of IMCY-0098 at three different doses and of placebo.

5.2 Secondary Objective

The secondary objective of this study is to evaluate the clinical response to IMCY-0098 by assessing disease activity.

5.3 Exploratory Objectives

The exploratory objectives of this study are:

- To evaluate and characterize the proinsulin-specific CD4+ T cells induced by IMCY-0098
- To evaluate the impact of IMCY-0098 on autoreactive T-cell responses specific for autoantigens expressed by islet β-cells (proinsulin, GAD65, IGRP)
- To evaluate the impact of IMCY-0098 on autoantibodies against GAD65, IA-2, ZnT8 and insulin

Supplementary information on the biomarkers relevant for each exploratory objective is provided in Table 2.

Table 2 Exploratory objectives

Exploratory objective	Response	Biomarker
Evaluation and characterization of the proinsulin-specific CD4+ T cells induced by IMCY-0098	Immune regulation	Frequency of cytolytic CD4+ T cells
		Functionality of CD4+ T cells
		Suppression of effector cells
Evaluation of the impact of IMCY-0098 on autoreactive T-cell responses specific for autoantigens expressed by islet β-cells	Effector response	Functionality of effector cells
		Frequency of islet-specific CD4+ response
		Frequency of islet-specific CD8+ response
Evaluation of the impact of IMCY-0098 on autoantibodies against GAD65, IA-2, ZnT8 and insulin	Effector response	Islet auto-antibodies

6. INVESTIGATIONAL PLAN

6.1 Description of Overall Study Design and Plan

This is a phase 1, randomized, double-blind, dose escalation, placebo-controlled, multicenter clinical trial to evaluate the safety, immune responses and clinical effects of IMCY-0098 in adult patients with recent onset T1D. All planned assessments and procedures are presented in Table 1 and in Section 9.6.

The study comprises a total of 7 visits occurring over a period of approximately 24 weeks (from the first administration of study product to the last planned visit).

6.1.1 Study design

A graphical representation of the study design is provided in Figure 1.

Figure 1 IMCY-T1D-001 study design

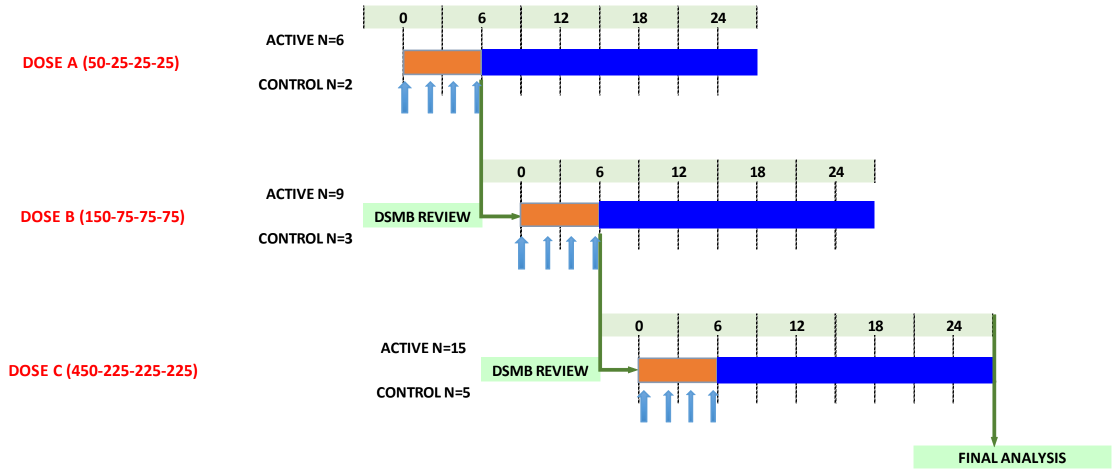

Study patients will be sequentially enrolled into one of the three cohorts and allocated to receive IMCY-0098 or placebo in a 3:1 randomization ratio, as outlined below:

- Cohort 1: One administration of IMCY-0098, 50 micrograms (µg) combined with alum adjuvant (500 µg/mL of aluminum hydroxide), followed by 3 administrations of IMCY-0098 25 µg combined with alum adjuvant OR four administrations of matching placebo with alum adjuvant
- Cohort 2: One administration of IMCY-0098, 150 µg combined with alum adjuvant, followed by 3 administrations of IMCY-0098 75 µg combined with alum adjuvant OR four administrations of matching placebo with alum adjuvant
- Cohort 3: One administration of IMCY-0098, 450 µg combined with alum adjuvant, followed by 3 administrations of IMCY-0098 225 µg combined with alum adjuvant OR four administrations of matching placebo with alum adjuvant

A total of 40 patients (6 active, 2 placebo patients in Cohort 1 and 9 active, 3 placebo patients in Cohort 2 and 15 active, 5 placebo patients in Cohort 3) are planned to be enrolled.

In each cohort, the first 4 patients will stay in the hospital for a period of 24 hours to allow close safety follow-up after each of the four administrations.

In each cohort, an interval of 2 days between each of the first 4 patients will be respected at each administration as per section 4.3.3. Patients subsequently enrolled at the same dose (4 in Cohort 1, 8 in Cohort 2, and 16 in Cohort 3) will be included without limitation in time.

When 4 patients in Cohort 1 have received their first administration AND at least 3 patients have received all 4 administrations (week 6 + 2 days), a safety evaluation will be conducted by an independent Data and Safety Monitoring Board (DSMB) to allow inclusion of patients in Cohort 2 once Cohort 1 has been fully recruited.

When 4 patients in Cohort 2 have received their first administration AND at least 3 patients have received all 4 administrations, a safety evaluation will be conducted by the DSMB to allow inclusion of patients in Cohort 3 once Cohort 2 has been fully recruited.

If the DSMB recommends not to enroll patients at a higher dose and if there is no recommendation to stop the study, additional patients may be enrolled in currently recruiting or previous cohorts.

Should one of the pre-determined events occur, an ad hoc DSMB meeting will be organized and recruitment of patients will be temporarily halted. Treatment of patients already enrolled will be stopped and patients will be followed-up according to the protocol. Patients who have been already fully treated (4 administrations completed) will also be followed-up according to the protocol. Recommendations of the DSMB will be followed for either restart (under DSMB defined conditions) or premature end of study.

The pre-determined events which will trigger an ad hoc DSMB meeting are:

- Investigator request
- Suspected Unexpected Serious Adverse Reaction (SUSAR)
- Anaphylactic shock

The safety profile of the different doses will be based on the following **safety considerations**:

- Occurrence, intensity and relationship of any solicited injection site and systemic adverse event (AE) during a 7-day follow-up period (i.e. day of study drug administration and 6 subsequent days) after each IMCY-0098 or placebo dose
- Occurrence, intensity and relationship of unsolicited injection site and systemic AEs occurring throughout the study period
- Occurrence and relationship of all serious adverse events (SAEs) occurring throughout the study period

- Occurrence, intensity and relationship of any abnormality in physical examination, vital signs, 12-lead ECG
- Hematological and biochemical levels within or outside the normal ranges
- Evolution of C-peptide concentration over the 6 months duration of the study.

This safety analysis should allow the selection of a dose level to be used in future clinical trials.

6.1.2 Planned analyses

When 4 patients in Cohort 1 have received their first administration AND at least 3 patients have received all 4 administrations, a safety evaluation will be conducted by an independent DSMB to allow inclusion of patients into Cohort 2. A similar analysis will be performed prior to inclusion of patients into Cohort 3.

The final analysis will be performed when all subjects will have completed the Week 24 visit.

6.2 Rationale and Discussion of Study Design

The proposed study design and sample size are estimated as adequate to provide a reliable safety assessment of each dose level and to provide preliminary efficacy/immunogenicity dose-response data which will be informative for subsequent clinical trials.

The use of a placebo control group in this study is considered to be acceptable in that all patients will remain under treatment with insulin over the entire study period.

7. STUDY POPULATION

7.1 Number of Subjects

This study plans to enroll 40 patients with T1D. Patients will be randomized to treatment in a 3:1 allocation ratio, with 30 patients receiving IMCY-0098 and 10 patients receiving matched placebo.

7.2 Recruitment Methods

This study will be conducted in multiple centers. Investigators will be responsible for calling patients who might be interested in participating in the study. Company advertisements could also be used to recruit patients if deemed relevant.

7.3 Eligibility Criteria

7.3.1 Inclusion criteria

A subject who meets all of the following criteria will be eligible to participate in this study:

1. Male or female 18 to 30 years of age
2. Initial diagnosis of Type 1 diabetes according to ADA/WHO criteria[1] within the past 6 months
3. Insulin requirement, as determined by the investigator
4. Presence of at least one autoantibody (GAD65, IA-2, or ZnT8)
5. Fasting C-peptide at screening > 0.2 nmol/L and/or stimulated C-peptide ≥ 0,4 nmol/L.
6. HLADR3-positive and/or HLADR4-positive
7. Willingness to undergo the insulin treatment prescribed by the physician
8. Body mass index (BMI) between 17–28 kg/m^2 at screening
9. Fully informed written consent obtained
10. Males with reproductive potential should use barrier method of contraception (condom) from screening up to 90 days after last treatment with investigational product.
11. Women of childbearing potential should use an highly effective contraception method from screening and for the whole duration of the study.

Of child-bearing potential is defined as being post onset of menarche and not meeting any of the following conditions:

- Menopausal for at least 2 years,
- Having undergone bilateral tubal ligation at least 1 year previously
- Having undergone bilateral oophorectomy or hysterectomy.

[1] As described in Diabetes Care Volume 39, Supplement 1, January 2016

HIGHLY EFFECTIVE contraceptive measures acceptable for the whole duration of the study have been defined based on the CTFGs recommendations on contraception and are the following:

- Combined (estrogen and progestogen containing) hormonal contraception associated with inhibition of ovulation (oral, intravaginal, transdermal),

- Progestogen-only hormonal contraception associated with inhibition of ovulation (oral, injectable, implantable).

- Intrauterine device (IUD)

- intrauterine hormone-releasing system (IUS)

- Monogamous relationship with vasectomized partner. Partner must have been vasectomized for at least 6 months prior to the patient's entry into the study

- Abstinence or absence of sexual relations with men.

7.3.2 Exclusion criteria

A subject who meets any of the following criteria will not be enrolled in the study:

1. Ongoing or planned pregnancy during the whole duration of the study or lactation.
2. Presence of significant medical conditions, in particular chronic liver condition, chronic hematological disease and renal dysfunction, of grade 2 or more according to the World Health Organization (WHO) Toxicity Scale (see appendix 2)
3. Has any current signs or symptoms of infection at entry or within 2 weeks of entry or has received intravenous antibiotics within 2 months prior to the first planned administration of the study product
4. Has received any live, attenuated vaccine within 3 months prior to the first planned administration of the study product (i.e. oral poliomyelitis vaccine, measles-mumps-rubella vaccine, yellow fever vaccine, Japanese encephalitis vaccine, dengue vaccine, rotavirus vaccine, varicella vaccine, life attenuated zoster vaccine, Bacillus Calmette-Guérin [BCG] vaccine, oral typhoid vaccine)
5. History of, or current malignancy (except excised basal cell skin cancer)
6. Clinical evidence of a diabetes-related complication that could interfere with patient’s participation/completion of study
7. Primary or secondary immune deficiency disorders
8. Human Immunodeficiency virus (HIV), chronic hepatitis B virus (HBV) or hepatitis C virus (HCV) infection

9. Presence at screening of abnormal laboratory values grade 2 or more according to the World Health Organization (WHO) Toxicity Scale
10. Anti-diabetic treatments other than insulin in the week prior to first study drug administration
11. Ongoing treatment with immunosuppressive agents or treatment within the past year, with the exception of topical or intra nasal corticosteroids
12. Treatment with immunotherapy within the past 3 months
13. Treatment with an investigational drug within the past 3 months.
14. Patients with a known hypersensitivity to any component of the drug product should be excluded from the study
15. Patients under treatment with statins at the time of screening.

8. INVESTIGATIONAL PRODUCT AND ADMINISTRATION

8.1 Study Products

8.1.1 IMCY-0098

IMCY-0098 has been developed by Imcyse SA. Information on the manufacturing processes is provided in the IB. Only pharmacopeia grade ingredients were used in the production of the intermediate and final products. Manufacturing facilities operate under current Good Manufacturing Practice (cGMP) guidelines.

IMCY-0098 will be provided as freeze-dried sterile powder for reconstitution with the diluent, which contains 500 µg/mL of aluminium hydroxide (Alhydrogel 2%). The diluent is a phosphate buffer (50mM), pH 7.4 containing NaCl as tonicity agent.

The Quality Control Standards and Requirements for IMCY-0098 are described in separate release protocols; all required approvals have been obtained.

8.1.2 Placebo

Placebo will be provided as a freeze-dried sterile powder made of 10 mg of mannitol for reconstitution with the same diluent as for IMCY-0098 (see Section 8.1.1).

8.2 Dosage and Administration

Study products must be administered by suitably trained clinical staff.

At the time of administration, IMCY-0098 or placebo will be reconstituted with 2.25 mL of diluent containing the adjuvant.

Study products will be injected in the upper arm, in the region of the lateral part of the arm, midway between the elbow and the shoulder. When two separate injections are needed, they will be administered concomitantly in both arms: injection 1 in the right arm and injection 2 in the left arm.

The low dose (Cohort 1) will consist of the SC administration of 50 µg of peptide in two separate injections of 25 µg each (100 µL each) followed by three consecutive injections of 25 µg of peptide as two separate injections of 12.5 µg each (50 µL each).

The medium dose (Cohort 2) will consist of the SC administration of 150 µg of peptide in two separate injections of 75 µg each (300 µL each) followed by three consecutive administrations of 75 µg of peptide as two separate injections of 37.5 µg each (150 µL each).

The higher dose (Cohort 3) will consist of the SC administration of 450 µg of peptide in two separate injections of 225 µg each (900 µL each) followed by three consecutive administrations of 225 µg of peptide as two separate injections of 112.5 µg each (450 µL each).

In each cohort, patients randomized to placebo will receive identical volumes of placebo solution to maintain study blind.

Detailed instructions are provided in the "Guide to Preparation and Administration of Study Product" leaflet.

Patients will be required to stay for 1 hour after study product administration for evaluation of any AEs (see Section 10.3.2). In each cohort, the first 4 patients will be required to stay overnight in the hospital after each administration for surveillance of potential AEs.

8.3 Packaging and Labelling

All clinical trial packaging and labeling operations will be performed according to cGMP. The contents of the label will be in accordance with all applicable regulatory requirements.

8.4 Storage and Return

Specific forms will be used to maintain records of the delivery and return of study products/materials.

The Investigator will dispense materials only to the participants of this study, following the procedures described in this study protocol. Administration of study products must be documented in the appropriate section of the Case Report Form (CRF).

The site will maintain a temperature log to ensure that study products are stored within the correct temperature range. Other study materials will be stored in a securely locked area that is accessible only to authorized personnel.

After completion of the study, all unused study product and/or materials will be returned to Imcyse.

8.5 Treatment Allocation and Randomization

A total of 40 patients are planned to be randomized to treatment in a 3:1 ratio (30 patients will receive IMCY-0098 and 10 patients will receive matching placebo).

Treatment allocation at each site will be performed using central randomization via an Interactive Web Response System (IWRS).

At the time of randomization, the IWRS will assign the subject to a treatment arm and will provide the number of the treatment vials which is to be used for the first administration of study product. With each dose of study product, a new treatment vial number will be attributed by the IWRS.

8.5.1 HLA A2 genotype and randomization

HLA-A2 is one of the most prevalent class I alleles, with a frequency of >60% in T1D patients[2].

[2] As described in *Physiol Rev* 91: 79–118, 2011

In order to mimic as much as possible the T1D population, from a HLA A2 perspective, a maximum of 16 patients (=40%) with a status HLA A2-negative will be allowed in the study. If and once the 16th HLA A2 negative patient has been randomized in the study, the randomization system will block the randomization of any HLA A2-negative patients and only allow the randomization of HLA A2-positive patients. Investigators will be informed regularly on the progress of the inclusion of HLA A2-negative patients. This process will allow to have sufficient number of HLA A2 positive subjects in placebo and vaccine groups to compare immune responses using HLA A2+ pentamers.

9. STUDY PROCEDURES

9.1 Subject Identification

Once informed consent has been obtained, patients will be attributed a unique identification number which will be used throughout the study. The identification number will consist of seven digits (three digits to represent the country, one to represent the site in the country, and three to represent the patient's order of inclusion at the site, *i.e.*, 056-2-001).

9.2 Method of Blinding and Breaking the Study Blind

Data will be collected in a double-blind manner until all patients who have been randomized have completed the study to Week 24 or have been prematurely withdrawn from the study. During this period, the patients and those responsible for the evaluation of any study endpoints (*e.g.*, safety and efficacy) will be unaware of the identity of the product (IMCY-0098 or placebo) administered to a particular patient.

If knowledge of the study product (IMCY-0098 or placebo) is necessary for optimal emergency treatment, then the Investigator may break the treatment code through the IWRS. Whenever possible, the Medical Monitor should be consulted before breaking the study blind, using the contact numbers provided in Section 13.6.3.

The Investigator must record the reason for breaking the study blind in the CRF.

9.3 Independent Data and Safety Monitoring Board

An independent DSMB consisting of experts in the appropriate disciplines will oversee the conduct of the study and ensure the safety of participating subjects. The role and responsibilities of the DSMB, as well as the data review process are outlined in detail in a separate DSMB Charter.

In addition to planned DSMB reviews, unscheduled meetings may be triggered in case of emerging safety concerns. Specifically, unscheduled meetings may be called at the request of any DSMB member, the Principal Investigator, or the Sponsor at any time during the study.

Safety data (as described in Section 11) will be reviewed by the DSMB which may recommend an amendment to the study protocol, or premature termination of the study; any such recommendations will be submitted to all Independent Ethics Committees (IECs) and national Competent Authorities (CA).

9.4 Treatment Modification and Discontinuation Criteria

9.4.1 Study hold

The administration of IMCY-0098 within the study will be put on hold by Imcyse and/or the DSMB if:

- The number and/or severity of AEs justifies halting the study.
- New data raise concerns about the safety of IMCY-0098 and continued administration would pose potential risks to the patients.

The IECs and CA will be notified if the study is put on hold.

9.4.2 Subject withdrawal from the study

Patients will be informed that they have the right to withdraw from the study at any time, without prejudice to their medical care, and that they are not obliged to state their reasons.

The following events will lead to the patient being withdrawn from the study:

- AE: Clinical or laboratory event(s) occurred that in the medical judgment of the Investigator for the best interest of the patient are grounds for discontinuation.

This includes serious adverse events (SAEs) and non-serious AEs regardless of the relationship to study product

- Development of an intercurrent illness, condition or procedural complication, which would interfere with the patient's continued participation
- Pregnancy
- Loss to follow-up: If the patient does not come to the study site for visits, study personnel should endeavor to contact the patient to determine the reasons for withdrawal. If the patient cannot be contacted, the patient will be considered as lost to follow-up

A patient will be considered to have completed the study when he or she completes the final assessments at Visit 8. Patients who discontinue from the study prematurely will be asked to return for an early termination visit that will include all assessments scheduled at Visit 8 (see Section 9.6.6). All patients who are withdrawn from the study because of AEs or clinically significant laboratory abnormalities will be followed up at suitable intervals in order to evaluate the course of the AEs or laboratory abnormalities and to ensure reversibility or stabilization. The subsequent outcomes of these events will be recorded in the CRF.

The termination page in the CRF should be completed for every patient, whether or not the patient completed the study. The reason for any early discontinuation should be indicated on this form.

Patients who are withdrawn from the study will not be replaced. Nevertheless, if any of the first four patients of a given cohort is withdrawn during the treatment period (from Week 0 till Week 6 included) and the 5th patient has not yet been included in the cohort, the withdrawn patient(s) will be replaced. The replacement patient(s) will follow the schedule of events as described in Section 10.3.6.

9.4.3 Contraindications to administration of subsequent doses of study product

If a patient has a temperature elevation (>37.5°C) at the time the first injection is planned (Week 0, Day 1), this initial injection (week 0) should be delayed up to the normalization of the temperature (<37,1°C) and respecting the maximum period of 28 days between screening and randomization.
If a patient has a temperature elevation (>37.5°C) at the time of any subsequent study product injection (Visits 3, 4 and 5), the injection will be postponed using the 2 days visit

window if relevant and possible. The temperature should be normalized (<37,1°C) before proceeding to the injection.

If a temperature elevation > 37.5°C persists longer and prevents the injection within the allowed visit window, the underlying cause must be investigated and the injection should be rescheduled to the earliest possible time when the temperature has normalized (<37.1°C) with a maximum of 14 days delay compared to the initial planned date. The remaining injection(s), if any, need to be rescheduled from that new date with an interval of 14 days +/- 2 days as described in Table 3.

In case, the temperature elevation would last for longer than 14 days, underlying causes must be evaluated and the injection scheme should be definitely stopped. All efforts should be made to follow the patient according to the visit schedule up to the end of the study. In case of patient refusal to remain in the study and perform the planned visits, procedures for patient withdrawal should be performed as described in section 9.4.2 above.

If any of the below AEs or conditions occur during the study, the patient must not receive additional doses of study product but should be encouraged to continue other study procedures to ensure safety follow-up to Week 24. The subject must be followed until resolution of these events or conditions.

- Anaphylactic reaction following the administration of study product
- Any event with a grade > 2 according to the modified WHO toxicity scale in appendix 2 and which, in the opinion of the Investigator, would be exacerbated by further administration of study product.
- Generalized immune disorder.
- In case a female subject becomes pregnant.

9.5 Concomitant Medications

9.5.1 Mandatory medications during the study

All patients should be treated according to an optimized conventional treatment with 1-2 injections of slow acting insulin per day and one dose of fast acting insulin prior to each meal.

9.5.2 Prohibited medications during the study

Patients will be withdrawn from the study if any of the following are administered:

- Immunosuppressive therapies, with the exception of topical or intra nasal corticosteroids
- Any live, attenuated vaccine during the whole study duration (i.e. oral poliomyelitis vaccine, measles-mumps-rubella vaccine, yellow fever vaccine, Japanese encephalitis vaccine, dengue vaccine, rotavirus vaccine, varicella vaccine, life attenuated zoster vaccine, Bacillus Calmette-Guérin [BCG] vaccine, oral typhoid vaccine)
- Statins
- Investigational or non-registered pharmaceutical or biological product other than the study products

End-of-study procedures should be performed.

Patients who use anti-diabetic medications other than insulin should not be withdrawn from the study but may be excluded from certain statistical analyses.

9.6 Detailed Description of Study Procedures

When materials are provided by Imcyse or its designated laboratory, it is mandatory that all clinical samples (including serum samples) are collected and stored exclusively using these materials and in the appropriate manner. The Investigator must ensure that his/her personnel and the laboratory(ies) under his/her supervision comply with this requirement. However, when Imcyse or its designated laboratory does not provide material for collecting and storing clinical samples, then appropriate materials from the Investigator's site are to be used.

It is the Investigator's responsibility to ensure that the intervals between visits are strictly followed as per Table 3.

Table 3 Time window between study visits

Interval	Length of interval
2 weeks/14 days	+/- 2 days
6 weeks/42 days	+/- 6 days

9.6.1 Visit 1 (Screening)

Patients will undergo screening within a maximum of 28 days before starting the study to be evaluated for eligibility. However, among all assessments listed below, two of them are not subject to the 28-day window:

- Assessment of the presence of diabetes auto-antibodies
- HLA class I / class II genotype determination

These 2 assessments can be performed as soon as the informed consent was signed. All other assessments need to be done within the 28 days preceding the randomization.

After patients have provided signed informed consent to participate in the study (which can be done outside of the 28 days window), the Investigator/designate will:

- Collect demographic data: age and gender
- Evaluate eligibility for enrollment
- Record T1D-specific and general medical history
- Record prior and current medication
- Record current insulin doses
- Perform a complete physical examination, including measurement of body temperature, weight and height
- Measure vital signs (brachial pulse and blood pressure) after the patient has been in a supine position for 3 minutes
- Perform a 12-lead electrocardiogram (ECG)
- Perform MMTT
- Obtain blood samples for:
    - Serum pregnancy test for female subjects of childbearing potential
    - Hematology, biochemistry
    - Measurement of fasting C peptide
    - Measurement of glycemia
    - Virology: anti-HIV antibodies, anti-HCV antibodies, HBsAg/anti-HBc antibodies
    - Assessment of the presence of diabetes auto-antibodies
    - HLA class I / class II genotype determination
- Obtain a urine sample for dipstick analysis and microscopic examination

All AEs (non-serious and serious) occurring after the patient has provided informed consent need to be reported – see Section 10.3.4 and Section 11 for further details.

9.6.2 Visit 2 (Study Day 1) = baseline

IMPORTANT – FIRST 4 PATIENTS IN EACH COHORT. These patients will be required to stay in the hospital for a period of 24 hours at this visit to allow close safety follow-up after injection (see Section 10.3.6 for specific assessments in addition to those listed hereafter).

During Visit 2, the Investigator/designate will:

- Evaluate eligibility for enrollment
- Perform a complete physical examination, including measurement of body temperature and weight
- Measure vital signs (brachial pulse and blood pressure) after the patient has been in a supine position for 3 minutes. Vital signs should be measured prior to, and 1 hour after, study product administration
- Record concomitant medication/medical conditions
- Record current insulin doses
- Record non-serious and serious AEs which have occurred since the previous visit
- Obtain blood samples for:
    - Hematology, biochemistry
    - Measurement of fasting C peptide
    - Measurement of HBA1c
    - Measurement of glycemia
    - Quantification and characterization of immuno-regulatory CD4+ T cell response
    - Quantification and characterization of the effector CD4+/CD8+ T cell response
- Obtain a urine sample for:
    - Urine pregnancy test for female subjects of childbearing potential
    - Dipstick analysis and microscopic examination
- Check contraindications to vaccination
- Obtain treatment number by IWRS. At this visit, the IWRS will randomly assign the subject to a treatment arm; a patient is considered as enrolled once he/she has been randomized. Subjects who provide informed consent but are not randomized will be considered as screen failures.
- Administer study product
- Record injection site reactions and systemic AEs for 1 hour following study product administration.

- Provide diary card for the daily recording of solicited AEs by the patient
- Provide FreeStyle Libre Flash Glucose scanner and the first sensor

9.6.3 Visit 3 (Week 2), Visit 4 (Week 4) and Visit 5 (Week 6)

IMPORTANT – FIRST 4 PATIENTS IN EACH COHORT. These patients will be required to stay in the hospital for a period of 24 hours at these visits to allow close safety follow-up after injection (see Section 10.3.56 for specific assessments in addition to those listed hereafter).

Enrolled patients will return to the clinic every 14 days after completing Visit 2 for Visits 3, 4 and 5. During these visits, the Investigator/designate will:

- Perform a complete physical examination, including measurement of body temperature and weight
- Measure vital signs (brachial pulse and blood pressure) after the patient has been in a supine position for 3 minutes. Vital signs should be measured prior to, and 1 hour after, study product administration
- Obtain blood samples for:
  - Hematology, biochemistry
  - Measurement of fasting C peptide
  - Measurement of glycemia

  At Visit 5 (Week 6) only:
  - Quantification and characterization of immuno-regulatory CD4+ T cell response
  - Quantification and characterization of the effector CD4+/CD8+ T cell response
- Obtain a urine sample for urine pregnancy test for female subjects of childbearing potential
- Collect diary cards provided at the previous visit. Check recorded information with the patient, and transcribe the information in the CRF
- Record concomitant medication/medical conditions
- Record current insulin doses
- Record non-serious and serious AEs which have occurred since the previous visit
- Check contraindications to vaccination
- Obtain the treatment number from IWRS
- Administer study product

- Record injection site reactions and systemic AEs for 1 hour following study product administration.
- Provide diary card for the daily recording of solicited AEs by the patient
- Provide new FreeStyle Libre sensor(s) and collect data from previous period

9.6.4 Visit 6 (Week 12)

Enrolled patients will return to the clinic 42 days after completing Visit 5. During Visit 6, the Investigator/designate will:

- Perform a complete physical examination, including measurement of body temperature and weight
- Measure vital signs (brachial pulse and blood pressure) after the patient has been in a supine position for 3 minutes
- Collect diary cards provided at the previous visit. Check recorded information with the patient, and transcribe the information in the CRF
- Record concomitant medication/medical conditions
- Record current insulin doses
- Record non-serious and serious AEs which have occurred since the previous visit
- Perform MMTT
- Obtain blood samples for:
  - Hematology, biochemistry
  - Measurement of HBA1c
  - Measurement of glycemia
  - Assessment of the presence of diabetes auto-antibodies
  - Quantification and characterization of immuno-regulatoryCD4+ T cell response
  - Quantification and characterization of the effector CD4+/CD8+ T cell response
- Obtain a urine sample for dipstick analysis and microscopic examination
- Provide new FreeStyle Libre sensors and collect data from previous period

9.6.5 Visit 7 (Week 18)

Enrolled patients will return to the clinic 42 days after completing Visit 6. During Visit 7, the Investigator/designate will:

- Perform a complete physical examination, including measurement of body temperature and weight

- Measure vital signs (brachial pulse and blood pressure) after the patient has been in a supine position for 3 minutes
- Record concomitant medication/medical conditions
- Record current insulin doses
- Record non-serious and serious AEs which have occurred since the previous visit
- Obtain blood samples for:
  - Hematology, biochemistry
  - Measurement of fasting C peptide
  - Measurement of glycemia
- Provide new FreeStyle Libre sensor and collect data from previous period

9.6.6 Visit 8 (Week 24) = End-of-study visit or Premature Discontinuation

Enrolled patients will return to the clinic 42 days after completing Visit 7 for Visit 8. Visit 8 is the last planned study visit. Assessments listed for this visit should be performed at last contact for all patients who prematurely discontinue the study, whatever the reason.

During this visit, the Investigator/designate will:

- Perform a complete physical examination, including measurement of body temperature and weight
- Measure vital signs (brachial pulse and blood pressure) after the patient has been in a supine position for 3 minutes.
- Perform a 12-lead ECG
- Perform MMTT. An MMTT is not required for premature discontinuations if this test has been performed at a planned visit within the previous 6 weeks.
- Record concomitant medication/medical conditions
- Record current insulin doses
- Record non-serious and serious AEs which have occurred since the previous visit
- Obtain blood samples for:
  - Hematology, biochemistry
  - Measurement of HBA1c
  - Measurement of glycemia
  - Assessment of the presence of diabetes auto-antibodies
  - Quantification and characterization of immuno-regulatoryCD4+ T cell response
  - Quantification and characterization of the effector CD4+/CD8+ T cell response
- Obtain a urine sample for dipstick analysis and microscopic examination

- Complete study conclusion sheet in the CRF
- Collect FreeStyle Libre data from previous period.

9.6.7 End of trial

The end of this clinical trial will be the last visit (at week 24) of the last subject.

10. DESCRIPTION OF ASSESSMENTS

10.1 Informed Consent

No study procedures will begin until the patient has given written informed consent to participate in the study.

10.2 Administration of Study Product

Study product will be administered to patients by the subcutaneous route at 2-week intervals at Visit 2 (Week 0), Visit 3 (Week 2), Visit 4 (Week 4) and Visit 5 (Week 6).

See also section 9.5.3 for instructions in case a patient would present with a temperature elevation > 37.5°C at the time of one of the visits where an injection of the study product is planned.

Appropriate medical treatment should be readily available in case of a rare anaphylactic reaction or other relevant medical event, which includes direct and quick access to reanimation material and emergency unit.

10.3 Safety Assessments

10.3.1 Medical history

A history-directed medical interview and examination of records will be performed during Visit 1. Any pre-existing conditions or signs and/or symptoms present in a patient prior to the start of the study will be recorded in the CRF.

Treatment of any abnormality observed during this examination has to be performed according to local medical practice outside of this study.

10.3.2 Physical examination and vital signs

A full physical examination will comprise measurements of body weight and height (Visit 1 only) and body temperature. A routine medical examination of body systems (cardiovascular system, pulmonary system, abdominal assessments) will also be performed.

10.3.3 Recording of concomitant medications/medical conditions

Any concomitant medication (name, dose, unit, frequency, route of administration, reason for medication, start and end dates) will be recorded at each visit.

10.3.4 Recording of adverse events and serious adverse events

An assessment of injection site and systemic solicited AEs will be performed one hour post study product administration by the study site personnel. Study staff will ask the patient about his/her perception of pain/tenderness and itching; erythema/redness, inflammation/swelling and induration will be assessed visually. The presence of solicited systemic AEs (headache, fatigue, malaise, myalgia, and fever) will also be recorded by study staff. (See section 10.3.56 for a description of the additional procedures to be performed during the 24-hour hospital stay.)

Patients will be also provided with a diary card to record the presence or absence and severity of specific injection site reactions and systemic signs and symptoms on the day of the injection and the 6 subsequent days, in the evening. The patients will be asked to bring the diary card back at the next visit. The Investigator will then review and complete the diary card with the patient and transcribe it into the CRF (see Section 11).

Additionally, at each visit, the Investigator will record any AEs that have occurred since the last visit.

Patients will be instructed to contact the Investigator immediately if they experience any signs or symptoms that they perceive to be serious. SAE reporting procedures are described in Section 11.5.

10.3.5 Pregnancy and Contraception

Females will be advised not to become pregnant from the screening visit onwards and during the whole study and to continue using adequate contraception as described at study entry until the end of the study.

Males will be asked to use barrier method contraception (condom) and not to father a child from the screening visit onwards and up to 90 days following the date of their final treatment of IMP.

Donation of sperm is not allowed up to 3 months following the last dose of IMCY-0098.

Donation of oocytes (egg cells) is not allowed during the whole duration of the study.

Pregnancy in either a participant or the partner of a participant taking trial medication will be recorded on a pregnancy notification form and the subject will be followed up until child birth

If a female subject becomes pregnant:

- No further doses will be given and the subject will be invited to attend trial follow up visits, to obtain outcome data in accordance with the planned analysis.
- The pregnancy will be recorded on a pregnancy notification form and the subject will be followed up until child birth. The same will be done for the female partner of a study participant in case this partner becomes pregnant during the treatment period and up to 90 days following the date of the final treatment of IMP

10.3.6 24 hour hospitalization for the first 4 patients in each cohort

The first 4 patients in each cohort will stay in the hospital for a period of 24 hours after each administration (Visits 2, 3, 4 and 5) to allow close safety follow-up.

In addition to the assessments performed for all other patients at these visits, the following additional assessment will be performed:

- Vital signs (brachial pulse, blood pressure and body temperature) will be taken at 6, 12, 18 and 24 hours after study product administration. In case abnormalities are detected during recording of these vital signs at any time point above, the investigator should perform an ECG (see also 10.5.3).
- Blood sampling for hematology and biochemistry testing will be performed and analyzed through the local laboratory at 6, 12, 18 and 24 hours after study product administration.
- Injection site reaction as described in Table 5 will be assessed at 6, 12, 18 and 24 hours after study product administration.
- Any AE or SAE occurring during this period will be recorded in the patient's CRF.

10.4 Laboratory Assessments

10.4.1 Sample handling and analysis

Samples will be coded only with the identification number of the patient.

Collected samples may be used for purposes related to the quality assurance of the laboratory tests described in this protocol. This may include the management of the quality of these current tests, the maintenance or improvement of these current tests, the development of new test methods for the markers described in this protocol, as well as making sure that new tests are comparable to previous methods and work reliably. Any sample testing will be done in line with the signed consent of the individual patient for the present study.

With the patient's consent, collected samples will be stored for up to 15 years, as measured from the date of the last study visit for the last patient, unless local rules, regulations, or guidelines require different timeframes or different procedures.

10.4.2 Sample volumes and sample handling

The maximum volume of blood collected from each patient during the study will range between approximately 500 mL for the patients not hospitalized and 600 ml if the patient is hospitalized (first 4 patients in each cohort).

The exact blood volumes planned to be withdrawn from each patient during the course of the study and the methods to be used for preparation of the samples for analysis are detailed in the Laboratory Manual.

10.4.3 Laboratory parameters and assays

10.4.3.1 Laboratory parameters

The laboratory parameters which will be measured during this study are presented in Table 4 (The timing of each assessment is shown in Table 1 – Table of study procedures).

Detailed information can be found in the Laboratory Manual.

Table 4 Laboratory parameters and laboratories

Read-out	Marker	Laboratory
Hematology	Complete blood cell count (red blood cells [RBCs], white blood cells [WBCs], and platelets)	Central laboratory

Read-out	Marker	Laboratory
	and differential count (neutrophils, lymphocytes, monocytes, basophils, and eosinophils: absolute and relative numbers)	
Hematology (for 24h hospitalized patients in each cohort)	Complete blood cell count (red blood cells [RBCs], white blood cells [WBCs], and platelets) and differential count (neutrophils, lymphocytes, monocytes, basophils, and eosinophils: absolute and relative numbers)	**Investigator Site (at 6h, 12h, 18h and 24h after injection)**
Biochemistry	- Alanine aminotransferase (ALT)<br>- Aspartate aminotransferase (AST)<br>- Lactate dehydrogenase (LDH)<br>- Creatine phosphokinase (CPK)<br>- Alkaline phosphatase (ALP)<br>- Total bilirubin<br>- Serum creatinine<br>- Urea<br>- Albumin<br>- Gamma Glutamyl Transferases (γGT)	Central laboratory
Biochemistry (for 24h hospitalized patients in each cohort)	- Alanine aminotransferase (ALT)<br>- Aspartate aminotransferase (AST)<br>- Lactate dehydrogenase (LDH)<br>- Creatine phosphokinase (CPK)<br>- Alkaline phosphatase (ALP)<br>- Total bilirubin<br>- Serum creatinine<br>- Urea<br>- Albumin<br>- Gamma Glutamyl Transferases (γGT)<br>- Glucose	**Investigator Site (at 6h, 12h, 18h and 24h after injection)**
Urinalysis	- Dipstick analysis<br>- Microscopic examination	**Investigator site**
Diabetes-specific analyses	- Glucose<br>- HBA1c<br>- C peptide	Central laboratory
Virology at screening	Anti-HIV antibodies, anti-HCV antibodies, HBsAg/anti-HBc antibodies	Central laboratory
HLA genotype	HLA class II genotype (HLADR3 and HLADR4) and HLA class I genotype HLA A2	Central laboratory
Pregnancy testing	Serum β- Human Chorionic Gonadotropin (HCG)	Central laboratory
	Urine β- HCG	**Investigator site**
Diabetes Auto-antibodies	Diabetes auto-antibodies (to GAD65, IA-2, ZnT8 and insulin)	Central laboratory
Exploratory analyses	T cells specific for proinsulin, GAD65, and IGRP	

Read-out	Marker	Laboratory
	Cytolytic CD4+ T cells against IMCY-0098	Specialized laboratory/ Imcyse

10.4.3.2 Central laboratory assays

All screening (biochemistry, hematology, diabetes auto-antibodies, virology, serum pregnancy testing, HLA genotyping and diabetic specific analyses) and routine laboratory assays (biochemistry, hematology, diabetes auto-antibodies and diabetic specific analyses) will be performed by a central laboratory using standardized techniques. All data will be reviewed by a physician to evaluate and confirm the patient's eligibility at screening (see Section 7.3) and to monitor the patient's general health throughout the trial.

10.4.3.3 Local laboratory assays

Local laboratories at investigator sites will be involved for the following analyses:

- All urinalysis during the study, including β- HCG testing
- Hematology and biochemistry safety analyses (including blood glucose measurement) at 6h, 12h, 18h and 24h after injection for the first 4 patients in each cohort during their 24h hospitalization at each of visit 2, 3, 4 and 5

10.4.3.4 Exploratory immunologic assessments

Exploratory immunologic assessments (corresponding to the objectives listed in Table 2) will be performed at a specialized laboratory or at Imcyse.

Each parameter will be evaluated under different conditions and using different qualified methods.

About 100ml of fresh blood will be taken at each time point (visit 2, 5, 6 and 8) referenced for quantification and characterization of the immune response. These samples will be used for PBMC preparation

10.5 Clinical Assessments

10.5.1 Complete physical examination and temperature measurement

A full physical examination, including assessments of the patient's general appearance, and his/her digestive, cardiovascular, respiratory, neurological and musculoskeletal systems, will be performed.

Body temperature may be taken by any route (see Section 11.2.2 for details). On visits where study product will be administered (Visits 2, 3, 4 and 5), body temperature will be taken prior to, and 1 hour after, study product administration.

Any abnormal finding during physical examination at Visits 2 – 8 should be reported as an unsolicited AE (see Section 11.2.3).

10.5.2 Vital signs

Vital signs include brachial pulse and blood pressure. Measurements are to be performed after patient has been in a supine position for 3 minutes.

At Visits 2, 3, 4, 5, measurement of vital signs will be performed prior to, and 1 hour post, study product administration.

Any abnormal finding should be reported as an unsolicited AE (see Section 11.2.3)

10.5.3 12-lead electrocardiogram

Standard 12-lead ECG recordings will be made at Visits 1 and 8, or at the end of study visit if the patient discontinues the study prior to Visit 8.

Moreover, for the first 4 patients in each cohort (see 10.3.6), in case abnormalities are detected during recording of vital signs during the 24h hospitalization period at any of the 4 injections, the investigator should perform an ECG.

Any abnormal reading at Visit 8/end of study visit should be reported as an unsolicited AE (see Section 11.2.3).

10.5.4 Mixed-meal tolerance test (MMTT)

10.5.4.1 Description of the test.

During the study, secretion of C-peptide will be tested by the Mixed Meal Tolerance Test method (MMTT) using Ensure Plus, a registered mark from Abbott, as standardized meal. It will be provided to sites in bottle of 220 ml, each bottle being used for 1 MMTT.

The MMTT performed in this study is a 2-hour MMTT with 5 collection time points (+/- 3 min for time points 30 and 60 min; +/- 5 min for time points 90 and 120 min). Real time has to be reported on the Requisition form (see Laboratory manual).

Briefly, the test is performed preferably in the morning (between 7 and 10 AM). The participant must be fasting and have had no food or drink (with the exception of water) from 12 (midnight) or 8 hours prior to the start of the test.

Tests should be conducted only if fasting value by capillary blood glucose meter or Freestyle Libre sensor or any other alternative method is between 3.9-11.1mmol/L (= 70 – 200 mg/dl or 0,7 – 2;0 g/l).
<u>Note:</u> *For the maximum glucose value at the start of the test, although the maximum allowed is 11.1 mmol/l (200 mg/dl or 2,0 g/l) as stated above, we recommend to strive to a maximum value of 9.0 mmol/L (= 162 mg/dl or 1,62 g/l) to optimize the results of the test.*

10.5.4.2 Information to be given to the patient:

Minimize physical activity in the morning of the MMTT to the extent possible.

Follow normal exercise and refrain from hard exercise (comparable to and exceeding 5 km running or weightlifting) 24 hours prior to the MMTT.

Participants should withhold taking long acting insulin on the morning of the test. They can take very short acting insulin up to 2 hours before the test. They can take long-acting insulin up to 6 hours before the test. If the participant is using an insulin pump, they should be advised to continue their basal regime but not have a bolus. Participants should be advised that the blood glucose level may rise during the MMTT, but insulin will be given if necessary at the end of the test to correct this.

Participants should be asked to test their blood glucose 2 hours before attending for their MMTT and contact the research team with the result in the 2 following cases:

- If the value is in the hypoglycaemic range (< 3.9mmol/L or < 70mg/dl or < 0,7 g/L) the test should be postponed to a different day within the visit window and hypoglycaemia treated appropriately first. If the test cannot be performed within the allowed visit window, it should be reported as “not done”.
- If the value is > 11.1 mmol/L or 200 mg/dL or 2.0 g/L the participant should be advised to take an appropriate correction bolus of very short acting insulin and be prepared for the possibility that the test may need to be postponed if the glucose is not in the target range after 2 hours. If the test cannot be performed within the allowed visit window, it should be reported as “not done”.

10.5.4.3 Samples collection

The procedures are as follows. Obtain first all other test samples required at that visit (see Table 1 – Table of Study Procedures).

For each MMTT time point blood samples are collected in 2 ml gray top fluoride tube for glucose and 2 ml red top EDTA tube for C-peptide (see Laboratory manual for more details).

A first blood sample is drawn immediately before the participant starts drinking the liquid meal (t=0min). The patient is given the standardized liquid meal: Ensure Plus (220ml; 330 kcal) to be ingested within 5 minutes. Blood samples are drawn at times: 30, 60, 90 and 120 minutes, after the end of ingestion of Ensure Plus (note: time runs from the start of ingestion).

Label, process and ship these tubes as described in appropriate sections of the Laboratory manual.

After the test is completed, the participant eats and receives insulin as appropriate and prescribed by the local investigator.

10.5.4.4 Precautions during the MMTT

In case the plasma glucose exceeds 16.7 mmol/l (300 mg/dl or 3,0 g/L) the MMTT can be stopped at the discretion of the investigator and rescue insulin treatment may be initiated if deemed necessary.

In case of hypoglycaemia during the MMTT, plasma glucose value <3.9 mmol/l (70 mg/dl or 0,7 g/L), the MMTT can be stopped at the discretion of the investigator and rescue treatment can be initiated.

10.5.5 Glycemic profile

Patient-reported glycemic profile will be managed through the provision to the patient of the FreeStyle Libre Flash Glucose Monitoring System. At visit 2, patient will receive the FreeStyle Libre Flash Glucose scanner and the first sensor will be applied.

When starting the Freestyle Libre Flash glucose scanner, lower and upper limits for glucose levels need to be entered in order to define the target range for the patient. Please refer to the scanner manual for practical details. The following limits need to be entered in the scanner:

- Lower glucose level limit: 0, 7 g/L or 70 mg/dL or 3, 9 mmol/L
- Upper limit: 1, 2 g/L or 120 mg/dL or 6, 7 mmol/L

Scanner and sensors will be used according to the manufacturer's instructions as available in the different countries.

At each visit from visit 3 onwards, data from the previous period will be collected and new sensor(s) will be delivered to cover the period until the next study visit. The following data will be recorded directly from the Freestyle Libre reader:

- Average glucose in last 14 days (and unit)
- Average glucose in last 90 days (and unit) – Only at visit 6 and visit 8
- Average morning glucose in last 14 days (and unit)
- Total events of low glucose in last 14 days
- Number of scans per day in last 14 days
- % sensor data captured in last 14 days

In case of issue with the use of the sensors (e.g. local allergy), and only in such situation, the patient will be provided with lancets to allow monitoring of blood glucose through classical finger’s pricking. The same scanner can be used to read these lancets.

11. ADVERSE EVENTS AND SERIOUS ADVERSE EVENTS

The Investigator is responsible for reporting all AEs that are observed or reported during the study, regardless of their relationship to study product.

AEs will be recorded and reported from the time a subject gives informed consent to study completion.

11.1 Definition of an Adverse Event

International Conference on Harmonisation of Technical Requirements for Registration of Pharmaceuticals for Human Use (ICH) E6 Good Clinical Practice (GCP) Guidelines define an AE as any untoward medical occurrence in a subject or subject administered a pharmaceutical product in a clinical investigation regardless of its causal relationship to the study treatment.

An AE can therefore be any unfavorable and unintended sign (including an abnormal laboratory finding), symptom or disease temporally associated with the use of medicinal (investigational) product. The occurrence of an AE may come to the attention of study personnel during study visits (either as a spontaneous report by the patient or following collection of information via the diary cards) or during routine visits of the patient for medical care.

A solicited AE is defined as any of the events prelisted in the CRF and on the diary card. Information on solicited AEs will be collected for a 7-day period (day of study product administration and the subsequent 6 days).

An unsolicited AE is any event, including SAEs, which is not prelisted. Any solicited AE which occurs outside of the 7-day follow-up period will be recorded as an unsolicited AE.

11.2 Assessment of Adverse Event Intensity

11.2.1 Injection site reactions

The intensity of AEs at the site of study product administration will be determined by study center staff, either by visual assessment or through questioning the patient, 1 hour post-study product administration. (Additional time points will be assessed for hospitalized patients; see Section 10.3.56.)

For injection site erythema/redness, inflammation/swelling and induration, the longest diameter of the affected skin area will be measured using a ruler; the result will be recorded (in mm) in the CRF. Grades will be attributed by Imcyse according to the scale provided in Table 5

The patient will be asked about his/her perception of pain/tenderness and itching. The grade, as shown in Table 5, will be recorded in the CRF.

In the evening of the day of administration and for the subsequent 6 days, the patient will measure erythema/redness, inflammation/swelling (using the ruler provided by Imcyse)

and will grade pain/tenderness and itching (following the scale in Table 5); this information will be recorded on the diary card.

All injection site AEs will be considered as reactions (*i.e.* related to study drug).

Table 5 Intensity scale for injection site reactions

**Reaction**	**Mild (Grade 1)**	**Moderate (Grade 2)**	**Severe (Grade 3)**
Erythema/Redness*	> 0 to < 30 mm	≥ 30 to < 120 mm	≥ 120 mm
Inflammation/Swelling*	> 0 to < 30 mm	≥ 30 to < 120 mm	≥ 120 mm
Induration	> 0 to < 30 mm	≥ 30 to < 120 mm	≥ 120 mm
Pain/tenderness	Injection site is painful when pressed	Interferes with activity	Prevents daily activity
Itching**	Mild or localized; topical intervention indicated	Intense or widespread; intermittent; skin changes from scratching (e.g., edema, papulation, excoriations, lichenification, oozing/crusts); oral intervention indicated; limiting instrumental Activities of Daily Living (ADL)	Intense or widespread; constant; limiting self care ADL or sleep; oral corticosteroid or immunosuppressive therapy indicated

ADL = Activities of Daily Living
*Measured at greatest single diameter
**National Cancer Institute (NCI) criteria applicable for grading of pruritus (National Cancer Institute, 2010)

11.2.2 Systemic solicited adverse events

The intensity of systemic AEs will be determined by study center staff 1 hour post study product administration. (Additional time points will be assessed for hospitalized patients; see Section 10.3.56). Temperature will be recorded in °C; any route for temperature measurement can be used, but the same route should be used for a given patient throughout the duration of the study. The route used should be indicated in the CRF. All other solicited AEs will be graded using the definitions provided in Table 6. Any AE not present prior to injection, will need to be evaluated by the investigator to state relationship to the study drug (*i.e.* related or not related).

In the evening of the day of administration and for the subsequent 6 days, the patient will record his temperature. If additional temperature measurements are performed at other times of day, these should be recorded on the diary card. The patient will grade any other events which occur.

During the review of the diary card with the patient, the Investigator/study staff will determine the causality of any systemic solicited AE reported.

Table 6 Intensity scale for systemic solicited adverse events

Systemic AE	Mild (Grade 1)	Moderate (Grade 2)	Severe (Grade 3)
Fever (°C)	37.1 - 38.0	38.1 - 40.0	> 40.0
Headache	Mild	Moderate or severe but transient	Unrelenting and severe
Fatigue	Increased fatigue over baseline, but does not alter activities of daily living	Causing difficulty performing some activities	Loss of ability to perform some activities
Malaise	Mild, able to continue activities of daily living	Impaired daily activity or bedrest < 50% of waking hours	In bed or chair ≥ 50% of waking hours
Myalgia	Mild	Decrease in ability to move	Disabled

ADL = Activities of daily living
TPN = total parenteral nutrition

11.2.3 Unsolicited adverse events

Space will be provided on the diary card for the patient to record of other, unsolicited AEs which may occur.

Abnormal findings at physical examination or upon measurement of vital signs or cardiac function (12-lead ECG) should be reported as AEs or SAEs, if appropriate.

Unsolicited AEs will be graded by the Investigator using the National Cancer Institute (NCI) criteria applicable for grading of the solicited AE (National Cancer Institute, 2010) scale. Changes in the severity of an AE should be documented to allow an assessment of the duration of the event at each level of intensity to be performed. AEs characterized as intermittent require documentation of onset and duration of each episode.

It is the responsibility of the investigator to review all documentation (*e.g.*, hospital progress notes, laboratory, and diagnostic reports) relative to the AE and to establish a diagnosis pertaining to the event based on signs, symptoms, and/or other clinical

information, in so far as possible. The diagnosis should be documented as the AE/SAE in preference to individual signs/symptoms.

11.3 Assessment of Adverse Event Outcome

The investigator will assess the outcome of all unsolicited AEs (including SAEs) recorded during the study as:

- Resolved
- Resolving
- Not resolved
- Resolved with sequelae
- Fatal (SAEs only)

11.4 Assessment of the Relationship to Study Products

The Investigator will assess if AEs are causally related to the study products. The assessment of causality will be made using the following definitions:

- Unrelated: This category is applicable to AEs which are judged to be clearly and incontrovertibly due to extraneous causes (disease, environment, etc.) and do not meet the criteria for drug relationship listed under Unlikely, Possible or Probable.

- Unlikely: In general, this category is applicable to an AE which meets the following criteria (must have the first two):
  - It does not follow a reasonable temporal sequence from administration of the drug.
  - It may readily have been produced by the subject's clinical state, environment or toxic factors, or other modes of therapy administered to the subject.
  - It does not follow a known pattern of response to the suspected drug.
  - It does not reappear or worsen when the drug is re-administered.

- Possible: This category applies to AEs in which the connection with the investigational product administration appears unlikely but cannot be ruled out with certainty. An AE may be considered possible if, or when (must have the first two):
  - It follows a reasonable temporal sequence from administration of the drug.

    - It may have been produced by the subject's clinical state, environment or toxic factors, or other modes of therapy administered to the subject.
    - It follows a known pattern of response to the suspected drug.

- Probable: This category applies to AEs which are considered to be related to the investigational product with a high degree of certainty. An AE may be considered probable, if (must have the first three):
    - It follows a reasonable temporal sequence from administration of the drug.
    - It cannot be reasonably explained by the known characteristics of the subject's clinical state, environment or toxic factors, or other modes of therapy administered to the subject.
    - It disappears or decreases on cessation or reduction in dose.
    - It follows a known pattern of response to the drug.
    - It reappears on re-challenge.

11.5 Serious adverse events

An SAE is defined as an AE that meets one of the following conditions:

- Death.
- Life threatening event.
- An event requiring inpatient hospitalization or prolongation of existing hospitalization during the period of protocol defined surveillance.
- Results in a persistent or significant disability/incapacity.
- Congenital anomaly or birth defect in the offspring of a study participant.
- Any other important medical event that may not result in death, be life threatening, or require hospitalization, may be considered a serious adverse experience when, based upon appropriate medical judgment, the event may jeopardize the patient and may require medical or surgical intervention to prevent one of the outcomes listed above. Examples of such medical events include allergic bronchospasm requiring intensive treatment in an emergency room or at home, blood dyscrasias or convulsions that do not result in inpatient hospitalization, or the development of drug dependency or drug abuse.

An SAE is not necessarily severe; for example, an overnight hospitalization for a diagnostic procedure must be reported as an SAE even though the occurrence is not medically serious. Similarly, a severe AE is not necessarily serious: nausea of several hours' duration may be rated as severe but may not be considered serious.

11.6 Adverse Event Recording

Each AE occurring to a subject, either spontaneously revealed by the subject or observed by the Investigator, whether believed by the Investigator to be related or unrelated to the study product, must be recorded on the patient's CRF.

The Investigator will also determine the intensity, relationship and outcome (as described in Sections 11.2, 11.3 and 11.4, respectively) of any AEs to study product and record this information in the appropriate section of the CRF.

11.7 Adverse Event and Pregnancy Reporting

11.7.1 Adverse event reporting

Any medical condition that is present at the time that the subject gives informed consent should be considered as pre-existing and not reported as an AE. However, if it deteriorates at any time during the study, it should be recorded as an AE.

Any event considered to be serious, as defined in Section 11.5, should be recorded on the SAE reporting form. All SAEs, regardless of relationship, must be reported via fax (see details below) within 24 hours of the Investigator becoming aware of the event. Other supporting documentation may be requested by the Sponsor and should be provided as soon as possible.

Contact for SAE reporting
E-mail: [redacted]
Fax: [redacted]

All SAEs will be followed up until satisfactory resolution or until the Investigator deems the event to be chronic or the patient to be stable.

Following notification from the Investigator, Imcyse's Medical Monitor will report events that are serious, unexpected and are considered to be causally related to the study products (SUSARs) to the CA within the required timelines: fatal and life threatening events within 7 calendar days and all other SAEs within 15 calendar days. The IB provides a list of the AEs considered as "expected" following the administration of the study products.

11.7.2 Pregnancy reporting

Pregnancy in either a participant or the partner of a participant taking trial medication that occurs during the study must be recorded on a pregnancy notification form and reported to Imcyse within 24 hours of the Investigator learning of its occurrence. The pregnancy should be followed up to determine its outcome (*i.e.*, healthy birth, spontaneous or voluntary abortion, presence or absence of any birth defects, congenital abnormality, or complications for the mother and/or newborn). The Investigator should assess if there was any relationship between the outcome and the exposure to study products.

11.8 Emergency Medication

The possibility that the IMP could cause anaphylaxis cannot be excluded. Equipment to manage cardiovascular resuscitation should be readily available at all visits when study product is administered.

12. STATISTICAL ANALYSES

12.1 Determination of Sample Size

The study sample size has been fixed without any statistical power consideration, but is estimated as adequate to provide a reliable safety assessment of the tested doses, to provide a first information on the safety and preliminary efficacy/immunogenicity dose-response, and to obtain preliminary efficacy/immunogenicity assessments to inform the next steps of the clinical development.

12.2 Primary Safety Endpoints

- Occurrence, intensity and relationship of any solicited injection site and systemic AEs during a 7-day follow-up period (*i.e.*, day of study product administration and 6 subsequent days) after each IMCY-0098 or placebo dose
- Occurrence, intensity and relationship of any unsolicited injection site and systemic AEs throughout the study period
- Occurrence and relationship of all SAEs throughout the study period
- Occurrence and relationship of any abnormality in physical examination, vital signs, 12-lead ECG
- Hematological and biochemical levels outside the normal ranges
- C-peptide concentrations throughout the study period

12.3 Secondary Efficacy Endpoints

- Post challenge C-peptide (2 hr AUC of MMTT)
- Fasting C-peptide
- HbA1c
- Insulin dose
- Glycemic profiles as measured by the patient through the use of the Freestyle Libre system.

12.4 Exploratory immunogenicity endpoints

- Suppression of CD4+/CD8+ effector responses by CD4+ T cells specific for IMCY-0098 peptide
- CD4+ T cell response specific for IMCY-0098 peptide
- CD4+ and CD8+ T cell responses specific for insulin, GAD65 and IGRP
- Change in diabetes auto-antibody levels (GADA, IA2A, ZnT8A, insulin)

12.5 Analysis Populations

All patients who are enrolled in the study and who receive at least one dose of study product will be allocated to the safety (SAF) analysis set.

All patients who are enrolled in the study, who receive at least one dose of study product, and who have at least one post-dose efficacy or immunogenicity assessment will be allocated to the intent-to-treat (ITT) analysis set.

All patients from the intent-to-treat analysis set without any major protocol deviations will be allocated to the per-protocol (PP) analysis set.

12.6 Statistical Considerations

All general and safety analyses will be performed on the SAF analysis set. All efficacy and immunogenicity analyses will be performed on both the ITT and the PP analysis sets.

Tables and graphs, as well as patient listings will be presented by dose groups. All parameters will be descriptively analyzed using standard statistical methods. Additional exploratory *post hoc* analyses can be decided upon review of the study results.

12.7 Statistical Methods

This section briefly describes the planned analysis. The Statistical Analysis Plan (SAP) will provide the full details of each analysis.

12.7.1 Subject disposition

The disposition of subjects will be summarized by analysis population, by dose groups and by country/site and at each time point.

The proportion of patients who prematurely discontinue the study will be summarized together with the reason for discontinuation.

12.7.2 Protocol deviations

All protocol deviations will be listed and will be tabulated according to their type (*i.e.*, major or minor).

A review of the protocol deviations will be performed before database lock to identify the subjects that should be excluded from the per-protocol analysis set due to major protocol deviations with a potential impact on the efficacy and/or immunogenicity evaluation.

12.7.3 Demographic, baseline and follow-up characteristics

Demographic data will be summarized by dose group.

Findings for medical and surgical history, concomitant diseases and baseline physical examinations will be tabulated.

Prior and concomitant medications will be coded using WHO_DRUG and will be summarized by dose group.

12.7.4 Analysis of the primary safety endpoints

Clinical safety will be addressed by assessing AEs, physical examinations, laboratory parameters, and vital sign results in a descriptive manner, by dose group and overall.

AEs will be fully described and coded according to the Medical Dictionary for Regulatory Activities (MedDRA). A distinction will be made between AEs before (non-treatment emergent adverse events) or after injection (treatment emergent adverse events [TEAEs]). A treatment-emergent analysis will be performed, counting events that started or worsened after the first dose of study product. Frequency of subjects presenting AEs,

treatment-related AEs, AEs leading to withdrawal and SAEs will be tabulated for each treatment group by System Organ Class and Preferred Term.

Changes in the post-baseline clinical laboratory tests compared to values obtained on Day 1 will be calculated. Descriptive statistics will be computed on the change for each variable. All laboratory values will be categorized according to their normal ranges. A shift table versus baseline will be created.

Vital signs and ECGs will be fully described using descriptive statistics. Shift tables versus baseline will be provided for vital signs according to their normal ranges.

Abnormal findings in physical examinations will be listed.

If deemed relevant from the results of the descriptive analyses, further exploratory statistical analyses or modeling will be performed

12.7.5 Analysis of the secondary efficacy endpoints

The principal/main time point for the evaluation of efficacy is Week 24, but efficacy at other time points will be assessed.

For patient-reported glycemic data over time, the following data will be recorded directly from the Freestyle Libre reader:

- Average glucose in last 14 days (and unit)
- Average glucose in last 90 days (and unit)
- Average morning glucose in last 14 days (and unit)
- Total events of low glucose in last 14 days
- Number of scans per day in last 14 days
- % sensor data captured in last 14 days

If deemed relevant from the results of the descriptive analyses, exploratory statistical analyses or modeling will be performed to characterize the efficacy dose-response.

12.7.6 Analysis of the exploratory immunogenicity endpoints

The immunogenicity endpoints will be descriptive summarized by dose group at each time point.

- Suppression of effector T cells will be reported as % of response observed in presence versus in absence of IMCY-0098 peptide in the cell culture

- Analyses of T cell populations will be reported as percentage of total CD4+ or CD8+ T cells and as fold increase/ decrease comparing each time point to baseline.
- Analyses of auto-antibodies will be reported as absolute value and as fold increase/ decrease comparing each time point to baseline.

Correlation analyses between immunogenicity responses and clinical or biological parameters will be performed on an exploratory basis.

13. STUDY MANAGEMENT

13.1 Regulatory Guidelines

This study will be conducted in accordance with ICH E6 GCP guidelines, local national laws (as applicable) and the guidelines of the Declaration of Helsinki, revised form of 64th World Medical Association (WMA) General Assembly, Fortaleza, Brazil, October 2013 (see APPENDIX 1).

13.2 Independent Ethic Committees

The composition of the committee will conform to state and local guidelines. The IEC will approve all aspects of the study, including protocol, Informed Consent Form (ICF), and advertising to be used and any modifications made to the protocol or informed consent prior to initiation. The IEC's decision concerning conduct of the study will be sent in writing to the Investigator, and a copy will be forwarded to Imcyse. The Investigator agrees to make any required progress reports to the IEC, as well as reports of related SAEs or deaths.

13.3 Informed Consent

Patients will give their informed consent. The Investigator will be responsible for obtaining from every patient, prior to his/her participation in the study, an ICF for study participation signed by the patient in accordance with ICH GCP guidelines.

Patients will be fully informed of the nature of the study, the properties and possible side effects of the investigational products and all relevant aspects of study procedures. They may ask questions to the Investigator or the clinic staff at any time.

Two original copies of the ICF will be signed and dated by the patients in the presence of an Investigator or designee (according to the site delegation of duties list). Participants will be given an original copy of the signed "Information for Patients and Consent Form

for Study Participation" for their records. The second signed and dated original copy will be held on file by the Investigator.

13.4 Insurance and Indemnity

Imcyse has obtained insurance coverage for the conduct of this clinical trial in each country.

Patients will be compensated for local transportation and food allowance costs. Patients who are required by the protocol to remain at the hospital for 24 hours after study product administration will be compensated for loss of earning.

13.5 Protocol Adherence - Amendments

The protocol must be read thoroughly and the instructions must be followed exactly.

Any changes in the protocol will require a formal amendment. Such amendments will be agreed upon and approved in writing by the Investigator and Imcyse. Substantial amendments have to be submitted to the IEC and CA. Changes that are not substantial, which have no significant impact on the medical or scientific validity of the study will be notified to the IEC and the CA, when required.

13.6 Compliance Control and Monitoring

13.6.1 Patient compliance

All study products will be administered to participating patients by suitably trained clinical staff designated and authorized by the Investigator at the study site.

Patients will be required to attend the study site for required visits at the specified times. Patients who are unable to comply may be withdrawn after discussion between the Investigator and the Sponsor.

Patients are free to withdraw their consent at any time.

13.6.2 Site compliance and monitoring

In agreeing to participate, the Investigator undertakes to strictly comply with the study protocol, GCP, and the national regulations. The Investigator also guarantees the authenticity of the data collected in the context of the study and agrees to the legal provisions for quality control by, or on behalf of, the Sponsor.

In compliance with GCP, the regular onsite verification of study-related data will be performed by the study monitor or other person authorized to conduct monitoring. The purpose of the monitoring is to verify that the rights and well-being of the patients are protected, the trial data are accurate, complete and verifiable from source documents, and the conduct of the trial is in compliance with the currently approved protocol, GCP, and the applicable regulatory requirements.

The Investigator undertakes to make him/herself available to the study monitor and provide direct access to source data/documents for study related monitoring, audits, IEC review, and regulatory inspection.

13.6.3 Medical monitor

The Medical Monitor will represent Imcyse in the event of questions regarding subject eligibility, evaluation of AEs, major and minor protocol deviations, and questions relating to the protocol and conduct of the study.

The Medical Monitor for this study is:

Medical Monitor: [redacted]<br>Mobile (24/7): [redacted]<br>e-mail: [redacted]

13.6.4 Case report forms and data entry

The Investigator or a designee will be responsible for recording study data in the CRF provided by Imcyse. It is ultimately the Investigator's responsibility to ensure the accuracy of all data entered in the CRFs.

Data validation will be performed after data entry and verification by computerized logical checks and manual review. Database lock will occur once quality assurance procedures have been completed.

13.7 Reporting and Communication of Results

A clinical study report will be generated by Imcyse when all subjects have completed Week 24, or have discontinued.

Imcyse commits to submit the study results for publication to a peer-reviewed journal within six months following complete analysis and reporting of Week 24 data - see Section 13.12.

13.8 Quality Assurance

The sites will be audited as necessary during the course of the study. The audits will include control of adherence to the protocol, Standard Operating Procedures (SOPs), ICH GCP Guidelines, and national laws. Source data verification and checking of data entered in the CRFs will be used for assessment of complete and reliable documentation.

The Investigator will allow Imcyse or their designated representatives to audit, at mutually convenient time(s) during the study, or after the study has been completed, all CRFs and all corresponding portions of office, clinic, and laboratory records of each study participant.

Regulatory authorities and representatives of the relevant independent IEC will be permitted to conduct inspections at the site. The Investigator should notify Imcyse if the regulatory authority contacts them to schedule an inspection.

13.9 Retention of Records

All source data, clinical records, and laboratory data relating to the study must be archived for 15 years after the completion of the clinical study.

All correspondence relating to this study should be kept in appropriate file folders. If an Investigator moves, withdraws from an investigation or retires, the responsibility for maintaining the records may be transferred to another person (*e.g.*, Imcyse, other Investigator) who will accept the responsibility. Notice of this transfer must be made to and agreed upon by Imcyse.

13.10 Patient Data Protection

Subjects will be informed that their clinical data is held on file by Imcyse and/or its representatives, that this source information may be viewed by Imcyse and/or its representatives and that data may also be seen by external auditors on behalf of either Imcyse or regulatory agencies at the investigational site. They will similarly be informed that information from the study will be prepared and may also be submitted to government agencies or for publication. However, participants of the study will only be

identified in such reports by their study identification number and, if appropriate, gender and age. The Investigator undertakes to hold all personal information in confidence.

13.11 Confidentiality

The Imcyse and any individuals acting on behalf of Imcyse will affirm and uphold the principle of the patient's right to protection against invasion of privacy. Throughout this study, all data will only be identified by an identification number and the subject's gender and age.

All information concerning this study and which was not previously published is considered confidential information. This confidential information shall remain the sole property of Imcyse; it shall not be disclosed to others without written consent of Imcyse and shall not be used except in the performance of this study.

The information compiled during the conduct of this clinical study is also considered confidential and may only be disclosed and/or used by Imcyse as they deem necessary. To allow the use of the information derived from this clinical study and to ensure compliance to current federal regulations, the Investigator is obliged to furnish Imcyse with the complete test results and all data compiled in this study.

13.12 Publication

The Investigator is not entitled to publish the results of the study without the Imcyse's prior written consent, which shall not be unreasonably withheld. Should the Investigator desire to publish the results of this study, the Investigator will request permission from Imcyse, and provide a copy of the manuscript at least 30 days prior to the expected date of submission to the publisher. Imcyse will review the manuscript and, if Imcyse consents to publication, will provide any comments on the manuscript to the Investigator. The Investigator agrees to include any reasonable comments made by Imcyse and further agrees to delay submission of the manuscript for up to six months if requested by Imcyse.

In the event that Imcyse chooses to publish the data from this study, this publication will precede the publication by the investigator. In such case, the Investigator will be provided with a copy of the manuscript at least 30 days prior to the expected date of submission to the publisher. The Investigator will review the manuscript and will provide any comments on the manuscript to Imcyse.

The results of final analysis will first be discussed with the Investigators. The authorship will be discussed at the time of the analysis. Up to four collaborators of Imcyse will be included as authors.

14. REFERENCES

Candia 2016 M, Kratzer B, Pickl WF, On Peptides and Altered Peptide Ligands: From Origin, Mode of Action and Design to Clinical Application (Immunotherapy), Int Arch Allergy Immunol 2016;170:211–233.

Carlier VA, Vander Elst L, Janssens W, Jacquemin MG, Saint-Remy J-MR. Increased synapse formation obtained by T cell epitopes containing a CxxC motif in flanking residues convert CD4+ T cells into cytolytic effectors. *Plos ONE*. 2012; 7(10):e45366.

Kikutani H, Makino S. The murine autoimmune diabetes model NOD and related strains. *Adv Immunol*. 1992; 51:285-322.

Lindblad, EB. Aluminium compounds for use in vaccines. *Immunology and Cell Biology*. 2004; 82, 497–505.

Lumsden JM et al, Protective immunity induced with the RTS,S/AS vaccine is associated with IL-2 and TNF-α producing effector and central memory CD4+ T cells, Plos One 2011; 6 (7): e20775.

Malek Abrahimians E, Carlier VA, Vander Elst L, Saint-Remy J-MR () MHC class II-restricted epitopes containing an oxidoreductase activity prompt $CD4^{+}$ T cells with apoptosis-inducing properties. *Front Immunol.* 2015; 6:449.

National Cancer Institute (NCI); U.S. Department of health and human services, National Institutes of Health. Common Terminology Criteria for Adverse Events (CTCAE) Version 4.0. Published: May 28, 2009 (v4.03: June 14, 2010).

Regulates JA et al, Fractional third and fourth dose of RTS,S/AS01 malaria candidate vaccine: a phase 2a controlled human malaria parasite infection and immunogenicity study, Journal of Infectious Disease 2016; 214:762-771.

Roep BO, Peakman M. Antigen targets of Type 1 diabetes autoimmunity. *Cold Spring Harb Perspect Med*. 2012; 2:a007781.

Simmons KM, Michels AW. Type 1 diabetes: a predictable disease. *World J Diabetes* 2015; 6(3): 380-390.

APPENDIX 1

WORLD MEDICAL ASSOCIATION (WMA) DECLARATION OF HELSINKI
Ethical Principles for Medical Research Involving Human Patients
Adopted by the 64th WMA General Assembly, Fortalez, Brazil October 2013

PREAMBLE

16. The World Medical Association (WMA) has developed the Declaration of Helsinki as a statement of ethical principles for medical research involving human patients, including research on identifiable human material and data.

    The Declaration is intended to be read as a whole and each of its constituent paragraphs should not be applied without consideration of all other relevant paragraphs.

17. Consistent with the mandate of the WMA, the Declaration is addressed primarily to physicians. The WMA encourages others who are involved in medical research involving human patients to adopt these principles.

GENERAL PRINCIPLES

18. The Declaration of Geneva of the WMA binds the physician with the words, "The health of my patient will be my first consideration," and the International Code of Medical Ethics declares that, "A physician shall act in the patient's best interest when providing medical care.".

19. It is the duty of the physician to promote and safeguard the health, well-being and rights of patients, including those who are involved in medical research. The physician's knowledge and conscience are dedicated to the fulfillment of this duty

20. Medical progress is based on research that ultimately must include studies involving human subjects.

21. The primary purpose of medical research involving human subjects is to understand the causes, development and effects of diseases and improve preventive, diagnostic and therapeutic interventions (methods, procedures and treatments). Even the best proven interventions must be evaluated continually through research for their safety, effectiveness, efficiency, accessibility and quality.

22. Medical research is patient to ethical standards that promote respect for all human subjects and protect their health and rights.

23. While the primary purpose of medical research is to generate new knowledge, the goal can never take precedence over the rights and interests of individual research subjects.

24. It is the duty of physicians who participate in medical research to protect the life, health, dignity, integrity, right to self-determination, privacy, and confidentiality of personal information of research subjects. The responsibility for the protection of research subjects must always rest with the physician or other health care professionals and never with the research subjects, even though they have given consent.

25. Physicians must consider the ethical, legal and regulatory norms and standards for research involving human subjects in their own countries as well as applicable international norms and standards. No national or international ethical, legal or regulatory requirement should reduce or eliminate any of the protections for research patients set forth in this Declaration.

26. Medical research should be conducted in a manner that minimises possible harm to the environment.

27. Medical research involving human subjects must be conducted only by individuals with the appropriate ethics and scientific education, training and qualifications. Research on patients or healthy volunteers requires the supervision of a competent and appropriately qualified physician or other health care professional.

28. Groups that are underrepresented in medical research should be provided appropriate access to participation in research.

29. Physicians who combine medical research with medical care should involve their patients in research only to the extent that this is justified by its potential preventative, diagnostic or therapeutic value and if the physician has good reason to believe that participation in the research study will not adversely affect the health of the patients who serve as research subjects.

30. Appropriate compensation and treatment for subjects who are harmed as a result of participating in research must be ensured.

RISKS, BURDENS AND BENEFITS

31. In medical practice and in medical research, most interventions involve risks and burdens.

Medical research involving human patients may only be conducted if the importance of the objective outweighs the risks and burdens to the research subjects.

32. All medical research involving human subjects must be preceded by careful assessment of predicable risks and burdens to the individuals and groups involved in the research in comparison with foreseeable benefits to them and to other individual or groups affected by the condition under investigation.

    Measures to minimise the risks must be implemented. The risks must be continuously monitored, assessed and documented by the researcher.

33. Physicians may not participate in a research study involving human subjects unless they are confident that the risks involved have been adequately assessed and can be satisfactorily managed.

    When the risks are found to outweigh the potential benefits or when there is conclusive proof of definitive outcomes, physicians must assess whether to continue, modify or immediately stop the study.

VULNERABLE GROUPS AND INDIVIDUALS

34. Some groups and individuals are particularly vulnerable and may have increased likelihood of being wronged or of incurring additional harm.

    All vulnerable groups and individuals should receive specifically considered protection.

35. Medical research with a vulnerable group is only justified if the research is responsive to the health needs or priorities of this group and the research cannot be carried out in a non-vulnerable group. In addition, this group should stand to benefit from the knowledge, practices or interventions that result from the research.

SCIENTIFIC REQUIREMENTS AND RESEARCH PROTOCOLS

36. Medical research involving human subjects must conform to generally accepted scientific principles, be based on a thorough knowledge of the scientific literature, other relevant sources of information, and adequate laboratory and, as appropriate, animal experimentation. The welfare of animals used for research must be respected.

37. The design and performance of each research study involving human subjects must be clearly described and justified in a research protocol.

The protocol should contain a statement of the ethical considerations involved and should indicate how the principles in this Declaration have been addressed. The protocol should include information regarding funding, sponsors, institutional affiliations, other potential conflicts of interest, incentives for subjects and provisions for treating and/or compensating subjects who are harmed as a consequence of participation in the research study.

In clinical trials, the protocol must also describe arrangements for post-trial provisions.

RESEARCH ETHICS COMMITTEES

38. The research protocol must be submitted for consideration, comment, guidance and approval to the concerned research ethics committee before the study begins. This committee must be transparent in its functioning, must be independent of the researcher, the sponsor and any other undue influence and must be duly qualified. It must take into consideration the laws and regulations of the country or countries in which the research is to be performed as well as applicable international norms and standards but these must not be allowed to reduce or eliminate any of the protections for research patients set forth in this Declaration.

    The committee must have the right to monitor ongoing studies. The researcher must provide monitoring information to the committee, especially information about any serious adverse events. No amendment to the protocol may be made without consideration and approval by the committee. After the end of the study, the researchers must submit a final report to the committee containing a summary of the study's findings and conclusions.

PRIVACY AND CONFIDENTIALITY

39. Every precaution must be taken to protect the privacy of research subjects and the confidentiality of their personal information.

INFORMED CONSENT

40. Participation by individuals capable of giving informed consent as subjects in medical research must be voluntary. Although it may be appropriate to consult family members or community leaders, no individual capable of giving informed consent may be enrolled in a research study unless he or she freely agrees.

41. In medical research involving human subjects capable of giving informed consent, each potential subject must be adequately informed of the aims, methods, sources

of funding, any possible conflicts of interest, institutional affiliations of the researcher, the anticipated benefits and potential risks of the study and the discomfort it may entail, and any other relevant aspects of the study. The potential subject must be informed of the right to refuse to participate in the study or to withdraw consent to participate at any time without reprisal. Special attention should be given to the specific information needs of individual potential subjects as well as to the methods used to deliver the information.

After ensuring that the potential subject has understood the information, the physician or another appropriately qualified individual must then seek the potential subject's freely-given informed consent, preferably in writing. If the consent cannot be expressed in writing, the non-written consent must be formally documented and witnessed.

All medical research subjects should be given the option of being informed about the general outcome and results of the study.

42. When seeking informed consent for participation in a research study the physician should be particularly cautious if the potential subject is in a dependent relationship with the physician or may consent under duress. In such situations the informed consent should be sought by an appropriately qualified individual who is completely independent of this relationship.

43. For a potential research subject who is incapable of giving informed consent, the physician must seek informed consent from the legally authorised representative. These individuals must not be included in a research study that has no likelihood of benefit for them unless it is intended to promote the health of the group represented by the potential subject, the research cannot instead be performed with persons capable of providing informed consent, and the research entails only minimal risk and minimal burden.

44. When a potential research subject who is deemed incapable of giving informed consent is able to give assent to decisions about participation in research, the physician must seek that assent in addition to the consent of the legally authorised representative. The potential subject’s dissent should be respected.

45. Research involving subjects who are physically or mentally incapable of giving consent, for example, unconscious patients, may be done only if the physical or mental condition that prevents giving informed consent is a necessary characteristic of the research population. In such circumstances the physician must seek informed consent from the legally authorised representative. If no such representative is

available and if the research cannot be delayed, the study may proceed without informed consent provided that the specific reasons for involving subjects with a condition that renders them unable to give informed consent have been stated in the research protocol and the study has been approved by a research ethics committee. Consent to remain in the research should be obtained as soon as possible from the patient or a legally authorised representative.

46. The physician must fully inform the patient which aspects of the care are related to the research. The refusal of a patient to participate in a study or the patient's decision to withdraw from the study must never interfere with the patient-physician relationship.

47. For medical research using identifiable human material or data, such as research on material or data contained in biobanks or similar repositories, physicians must seek informed consent for its collection, storage and/or reuse. There may be exceptional situations where consent would be impossible or impractical to obtain for such research. In such situations the research may be done only after consideration and approval of a research ethics committee.

USE OF PLACEBO

48. The benefits, risks, burdens and effectiveness of a new intervention must be tested against those of the best proven intervention(s), except in the following circumstances:

Where no proven intervention exists, the use of placebo, or no intervention is acceptable; or

Where for compelling and scientifically sound methodological reasons the use of any intervention less effective than the best proven one, the use of placebo, or no intervention is necessary to determine the efficacy or safety of an intervention

and the patients who receive any intervention which is less effective than the best proven one, placebo or no intervention will not be subject to additional risks of serious or irreversible harm as a result of not receiving the best proven intervention.

Extreme care must be taken to avoid abuse of this option.

POST-TRIAL PROVISIONS

49. In advance of a clinical trial, sponsors, researchers and host country governments should make provisions for post-trial access for all participants who still need an

intervention identified as beneficial in the trial. This information must also be disclosed to participants during the informed consent process.

RESEARCH REGISTRATION AND PUBLICATION AND DISSEMINATION OF RESULTS

50. Every research study involving human subjects must be registered in a publicly accessible database before recruitment of the first subject.

51. Researchers, authors, sponsors, editors and publishers all have ethical obligations with regard to the publication of the results of research. Researchers have a duty to make publicly available the results of their research on human subjects and are accountable for the completeness and accuracy of their reports. All parties should adhere to accepted guidelines for ethical reporting. Negative and inconclusive as well as positive results should be published or otherwise made publicly available. Sources of funding, institutional affiliations and conflicts of interest must be declared in the publication. Reports of research not in accordance with the principles of this Declaration should not be accepted for publication.

UNPROVEN INTERVENTIONS IN CLINICAL PRACTICE

52. In the treatment of an individual patient, where proven interventions do not exist or other known interventions have been ineffective, the physician, after seeking expert advice, with informed consent from the patient or a legally authorised representative, may use an unproven intervention if in the physician's judgment it offers hope of saving life, re-establishing health or alleviating suffering. This intervention should be made the object of research, designed to evaluate its safety and efficacy. In all cases, new information should be recorded and, where appropriate, made publicly available.

APPENDIX 2: MODIFIED WHO TOXICITY SCALE

MODIFIED WHO RECOMMENDATIONS FOR GRADING OF ACUTE AND SUBACUTE TOXICITIES.

	Grade 0	Grade 1	Grade 2	Grade 3	Grade 4
**HAEMATOLOGICAL**					
WBC	> 4.0	3.0 - 3.9	2.0 - 2.9	1.0 - 1.9	< 1
PLT	WNL	75.0 - normal	50.0 - 74.9	25.0 - 49.9	< 25.0
Hemoglobin (g/l)	WNL	100 - normal	80 - 100	65 - 79	< 65
(mmol/l)	WNL	6.2 - normal	4.95 - 6.2	4.0 - 4.9	< 4.0
(g/100mL)	WNL	10.0 - normal	8.0 - 10.0	6.5 - 7.9	< 6.5
Granulocytes/bands	≥ 2.0	1.5 - 1.9	1.0 - 1.4	0.5 - 0.9	< 0.5
Hematologic – other	none	mild	moderate	severe	life-threatening
**HAEMORRHAGE** (clinical)	none	mild, no transfusion	gross, 1 - 2 U per episode	gross, 3 - 4 U per episode	massive, > 4 U per episode
**INFECTION**	none	mild, no active treatment	moderate, PO antibiotic	severe, IV antibiotic, anti-fungal or hospitalization	life-threatening
**GASTROINTESTINAL**					
Nausea	none	able to eat reasonable intake	intake significantly decreased but can eat	no significant intake	- -
Vomiting	none	once in 24 hours	2-5 x in 24 hours	6 - 10 x in 24 hours	> 10 x in 24 hours or requiring IV support
Diarrhea	none	increase of 2 - 3 stools/day over pre-Rx	increase of 4 - 6 stools/ day, or nocturnal stools, or moderate cramping	increase of 7 - 9 stools/ day, or incontinence, or severe cramping	increase of > 10 stools/day or grossly bloody diarrhea, or need for parenteral support

	Grade 0	Grade 1	Grade 2	Grade 3	Grade 4
Stomatitis	none	painless ulcers, erythema, or mild soreness	painful erythema, edema, or ulcers but can eat	painful erythema, edema, or ulcers and cannot eat	requires parenteral or enteral support
Esophagitis/ Obstruction	none	painless ulcers erythema, mild soreness or dysphagia	painful erythema, edema, or ulcers or moderate dysphagia but can eat without narcotics	complete dysphagia, cannot eat solids or requires narcotics to eat	requires parenteral or enteral support or narcotics or perforation
Anorexia	none	mild	moderate	severe	life-threatening
Gastritis/ulcer	no	antacid	requires vigorous medical management or nonsurgical treatment	uncontrolled by medical management; requires surgery	perforation or bleeding
Small bowel obstruction	no	- -	intermittent, no intervention	requires intervention	requires operation
Intestinal fistula	no	- -	- -	yes	- -
GI – other	none	mild	moderate	severe	life-threatening
**<u>OTHER MUCOSAL</u>**	none	erythema, or mild pain not requiring treatment	patchy and serosanguinous discharge or non-narcotic for pain	confluent fibrinous mucositis or ulceration or narcotic for pain	necrosis
**LIVER**					
Bilirubin	WNL	- -	< 1.5 x N	1.5 - 3.0 x N	> 3.0 x N
Transaminases (SGOT/AST, SGPT/ALT)	WNL	< 2.5 x N	2.6 - 5.0 x N	5.1 - 20.0 x N	> 20.0 x N
Alkaline phosphatase or 5'nucleotidase	WNL	< 2.5 x N	2.6 - 5.0 x N	5.1 - 20.0 x N	> 20.0 x N
Liver – clinical	no change from baseline	- -	- -	precoma	hepatic coma
Liver – other	- -	mild	moderate	severe	life-threatening
**RENAL & BLADDER**					
Creatinine	WNL	< 1.5 x N	1.5 - 3.0 x N	3.1 - 6.0 x N	> 6.0 x N
Proteinuria	no change	1+ or < 0.3 g% or < 3 g/l	2 - 3 + or 0.3 - 1.0 g% or 3 - 10 g/l	4 + or > 1.0 g% or > 10 g/l	nephrotic syndrome
Hematuria	negative	micro only	gross, no clots	gross + clots	requires transfusion

		Grade 0	Grade 1	Grade 2	Grade 3	Grade 4
BUN	(mg %)	WNL, < 20	21 - 30	31 - 50	> 50	- -
	(mmol/l)	WNL, < 7.5	7.6 - 10.9	11 - 18	> 18	- -
Hemorrhagic cystitis		none	blood on microscopic examination	frank blood, no treatment required	bladder irrigation required	requires cystectomy or transfusion
Renal failure		- -	- -	- -	- -	dialysis required
Incontinence		normal	with coughing, sneezing, etc	spontaneous, some control	no control	- -
Dysuria		none	mild pain	painful or burning urination controlled by pyridium	not controlled by pyridium	- -
Urinary retention		none	residue > 100mL or occasional catheter or difficulty initiating stream	self-catheter required for voiding	surgery required(IUR or dilatation)	- -
Increased frequency/ urgency		no change	increase in frequency or nocturia up to 2 x normal	increase > 2 x normal but < hourly	with urgency and hourly or more or requires catheter	- -
Bladder cramps		none	- -	yes	- -	- -
Ureteral obstruction		none	unilateral, no surgery required	bilateral, no surgery required	incomplete bilateral, but stents, nephrostomy tubes or surgery needed	complete bilateral obstruction
GU fistula		none	- -	- -	yes	- -
Kidney/bladder - other		- -	mild	moderate	severe	life-threatening
**ALOPECIA**		no loss	mild hair loss	pronounced or total hair loss	- -	- -
**PULMONARY**						
Dyspnea		none or no change	asymptomatic, with abnormality in PFTs	dyspnea on significant exertion	dyspnea at normal level of activity	dyspnea at rest
pO2 / pCO2		no change or pO2 > 85 and pCO2 < 40	pO2 71-85<br>pCO2 41-50	pO2 61-70<br>pCO2 51-60	pO2 51-60<br>pCO2 61-70	pO2 ≤ 50 or pCO2 > 71

	Grade 0	Grade 1	Grade 2	Grade 3	Grade 4
DLCO	> 90% of pretreatment	76 - 90% of pretreatment	51 - 75% of pretreatment	26 - 50% of pretreatment	≤ 25% of pretreatment
Pulmonary fibrosis	none	radiographic changes, asymptomatic	- -	changes with symptoms	- -
Pulmonary edema	none	- -	- -	radiographic changes and diuretic needed	requires intubation
Pneumonia (non-infectious)	none	radiographic changes, no steroids needed	steroids required	oxygen required	assisted ventilation required
Pleural effusion	none	present	- -	- -	- -
ARDS	none	mild	moderate	severe	life-threatening
Cough	no change	mild, relieved by OTC medications	requires narcotic antitussive	uncontrolled cough	- -
Pulmonary - other	- -	mild	moderate	severe	life-threatening
**ALLERGY**	none	transient rash product fever < 38°C	urticaria, product fever ≥ 38°C, mild bronchospasm	serum sickness, bronchospasm, parenteral medication	anaphylaxis
**CARDIAC**					
Cardiac dysrhythmias	none	asymptomatic, transient, no therapy required	recurrent or persistent, no therapy required	requires treatment	requires monitoring; or hypotension or ventricular tachycardia or fibrillation
Cardiac function	none	asymptomatic, decline of resting LVEF < 20% of baseline	asymptomatic decline of resting LVEF > 20% of baseline	mild CHF, responsive CHF to therapy	severe or refractory
Cardiac ischemia	none	non-specific T wave flattening	asymptomatic ST and T wave changes for ischemia	angina without evidence for infarction	acute myocardial infarction
Cardiac-pericardial	none	asymptomatic effusion, no intervention	pericarditis (rub, chest pain, ECG changes)	symptomatic effusion; drainage	tamponade; drainage urgently required
Cardiac - other	none	mild	moderate	severe	life-threatening

	Grade 0	Grade 1	Grade 2	Grade 3	Grade 4
Hypertension	none or no change	asymptomatic, transient increase by > 20 mm Hg (D) or to > 150/100 if previously WNL. No treatment	recurrent or persistent increase by > 20 mm Hg (D) or to > 150/100 if previously WNL. No treatment	requires therapy	hypertensive crisis
Hypotension	none or no change	changes not requiring therapy (including transient orthostatic hypotension)	requires fluid replacement or other therapy but not hospitalization	requires therapy and hospitalization; resolves within 48 hrs of stopping the agent	requires therapy and hospitalization for > 48 hrs the agent
Phlebitis/thrombosis embolism	- -	- -	superficial phlebitis(not local)	deep vein thrombosis	major event (cerebral/hepatic/ pulmonary/other infarction) or pulmonary embolism
Edema	none	1+ or dependent in evening only	2+ or dependent throughout day	3+	4+, generalized anasarca
**NEUROLOGIC**					
Neurosensory	none or no change	mild paraesthesias loss of deep tendon reflexes	mild or moderate objective loss; moderate paraesthesias	severe objective sensory loss or paraesthesias that interfere with function	- -
Neuromotor	none or no change	subjective weakness; no objective findings	mild objective weakness but no significant impairment of function	objective weakness with impairment of function	paralysis
Neurocortica	none	mild somnolence or agitation	moderate somnolence or agitation	severe somnolence, agitation, confusion,	coma, seizures, toxic psychosis

	Grade 0	Grade 1	Grade 2	Grade 3	Grade 4
				disorientation, hallucinations	
Neurocerebellar	none	slight incoordination dysdiadochokinesis	intention tremor, dysmetria slurred speech, nystagmus	locomotor ataxia	cerebellar necrosis
Neuromood	no change	mild anxiety or depression	moderate anxiety or depression	severe anxiety or depression	suicidal ideation
Neuroheadache	none	mild	moderate or severe but transient	unrelenting and severe	- -
Neuroconstipation	none or no change	mild	moderate	severe	ileus > 96 hours
Neurohearing	none or no change	asymptomatic, hearing loss on audiometry only	tinnitus	hearing loss interfering with function, correctable with hearing aid	deafness not correctable
Neurovision	none or no change	- -	- -	symptomatic subtotal loss of vision	blindness
Pain	none	mild	moderate	severe	intolerable
Behavioral change	none	change, not disruptive to patients or family	disruptive to patients or family	harmful to others or self	psychotic behavior
Dizziness/vertigo	none	non-disabling	- -	disabling	- -
Taste	normal	slightly altered taste, metallic taste	markedly altered taste	- -	- -
Insomnia	normal	occasional difficulty sleeping, may need pills	- -	difficulty sleeping despite medication	- -
Neurologic - other	- -	mild	moderate	severe	life-threatening
**DERMATOLOGIC**					
Skin	none or no change	scattered macular or papular eruption or erythema that is asymptomatic	scattered macular or macular or papular eruption or erythema with pruritus or other associated symptoms	generalized symptomatic macular, papular, or vesicular eruption	exfoliative dermatitis or ulcerating dermatitis
Local	none	pain	pain and swelling with inflammation or phlebitis	ulceration	plastic surgery indicated

	Grade 0	Grade 1	Grade 2	Grade 3	Grade 4
**FLU-LIKE SYMPTOMS**					
Fever in absence of infection	none	37.1 - 38.0°C<br>98.7 - 100.4°F	38.1 - 40.0°C<br>100.5 - 104.0°F	> 40.0°C<br>(104.0°F) for < 24 hours	> 40.0°C<br>(104.0°F) for<br>> 24 hours or with hypotension
Chills	none	mild or brief	pronounced or prolonged	- -	- -
Myalgia/arthralgia	normal	mild	decrease in ability to move	disabled	- -
Sweats	normal	mild and occasional	frequent or drenching	- -	- -
Malaise	none	mild, able to continue normal activities	impaired normal daily activity or bedrest < 50% of waking hours	in bed or chair > 50% of waking hours	bed ridden or unable to care for self
Flu-like symptoms	- -	mild	moderate	severe	life-threatening
<u>WEIGHT GAIN</u>	< 5%	5.0 - 9.9%	10.0 - 19.9%	≥ 20%	- -
<u>WEIGHT LOSS</u>	< 5%	5.0 - 9.9%	10.0 - 19.9%	≥ 20%	- -
**METABOLIC**					
Hyperglycemia	< 116 mg/dl<br>< 6.2 mmol/l	116 – 160<br>6.2 - 8.9	161 – 250<br>9.0 - 13.9	251 – 500<br>14.0 - 27.8	> 500 or ketoacidosis<br>> 27.8 or ketoacidosis
Hypoglycemia	> 64 mg/dl<br>> 3.6 mmol/l	55 – 64<br>3.1 - 3.6	40 – 54<br>2.2 - 3.0	30 – 39<br>1.7 - 2.1	< 30<br>< 1.7
Amylase	WNL	< 1.5 x N	1.5 - 2.0 x N	2.1 - 5.0 x N	> 5.1 x N
Hypercalcemia	< 10.6 mg/dl<br>< 2.65 mmol/l	10.6 - 11.5<br>2.65 - 2.87	11.6 - 12.5<br>2.88 - 3.12	12.6 - 13.5<br>3.13 - 3.37	≥ 13.5<br>≥ 3.37
Hypocalcemia	> 8.4 mg/dl<br>> 2.1 mmol/l	8.4 - 7.8<br>2.1 - 1.95	7.7 - 7.0<br>1.94 - 1.75	6.9 - 6.1<br>1.74 - 1.51	≤ 6.0<br>≤ 1.50
Hypomagnesia	> 1.4 mmol/l	1.4 - 1.2	1.1 - 0.9	0.8 - 0.6	≤ 0.5
Hyponatremia	WNL or<br>> 135	131 - 135	126 - 130	121 - 125	≤ 120
Hypokalemia	WNL or<br>> 3.5	3.1 - 3.5	2.6 - 3.0	2.1 - 2.5	≤ 2.0
Metabolic - other	- -	mild	moderate	severe	life-threatening
**COAGULATION**					
Fibrinogen	WNL	0.99 - 0.75 x N	0.74 - 0.50 x N	0.49 - 0.25 x N	≤ 0.24
Prothrombin time	WNL	1.01 - 1.25 x N	1.26 - 1.50 x N	1.51 - 2.00 x N	> 2.00 x N

	Grade 0	Grade 1	Grade 2	Grade 3	Grade 4
Partial thromboplastin time	WNL	1.01 - 1.66 x N	1.67 - 2.33 x N	2.34 - 3.00 x N	> 3.00 x N
Coagulation - other	- -	mild	moderate	severe	life-threatening
**ENDOCRINE**					
Impotence/libido	normal	decrease in normal function	- -	absence of function	- -
Sterility	- -	- -	yes	- -	- -
Amenorrhea	no	yes	- -	- -	- -
Gynecomastia	normal	mild	pronounced or painful	- -	- -
Hot flushes	none	mild or < 1/day	moderate and ≥ 1/day	frequent and interferes with normal function	- -
Cushingoid	normal	mild	pronounced	- -	- -
Endocrine - other	- -	mild	moderate	severe	life-threatening
EYE					
Conjunctivitis/ keratitis	none	erythema or chemosis, no steroids or antibiotics	steroids or antibiotics required	corneal ulceration or visible opacification	- -
Dry eye	normal	- -	requires artificial tears	- -	requires enucleation
	no change	- -	- -	yes	- -
Glaucoma	- -	mild	moderate	severe	life-threatening
Eye - other					
