# Supplementary material for: First-in-human, double-blind, randomized phase 1b study of peptide immunotherapy IMCY-0098 in new-onset type 1 diabetes
Source: BMC Med. 2023 May 24;21:190. doi: 10.1186/s12916-023-02900-z (PMC10210318; doi:10.1186/s12916-023-02900-z)
Supplement: Supplementary file 2 — Additional file 2: Fig. S1. Study visits and assessments. Fig S2. Autoantibody levels. Table S1. Adverse events to Week 24. Table S2. Vital signs to Week 24. Table S3. MMTT to Week 24. Table S4. Insulin dose to Week 24. Table S5. Adverse events Week 24-48. Table S6. Clinical chemistry parameters to Week 48. Table S7. Hematology parameters to Week 48. Table S8. Vital signs to Week 48. [file 12916_2023_2900_MOESM2_ESM.docx]

**Title: First-in-human randomized phase 1b study of peptide immunotherapy IMCY-0098 in new-onset type 1 diabetes**

**Jean Van Rampelbergh,^1*†^ Peter Achenbach,^2,3†^ Richard David Leslie,^4†^ Mohammad Alhadj Ali,^5^ Colin Dayan,^5^ Bart Keymeulen,^6^ Katharine R. Owen,^7,8^ Martin Kindermans,^9^ Frédéric Parmentier,^9^ Vincent Carlier,^1^ Roxana R. Ahangarani,^1^ Evelien Gebruers,^1^ Nicolas Bovy,^1^ Luc Vanderelst,^1^ Marcelle Van Mechelen,^1^ Pierre Vandepapelière,^1†^ Christian Boitard^10,11†^**

**Supplementary material**

**Supplementary Figures:**


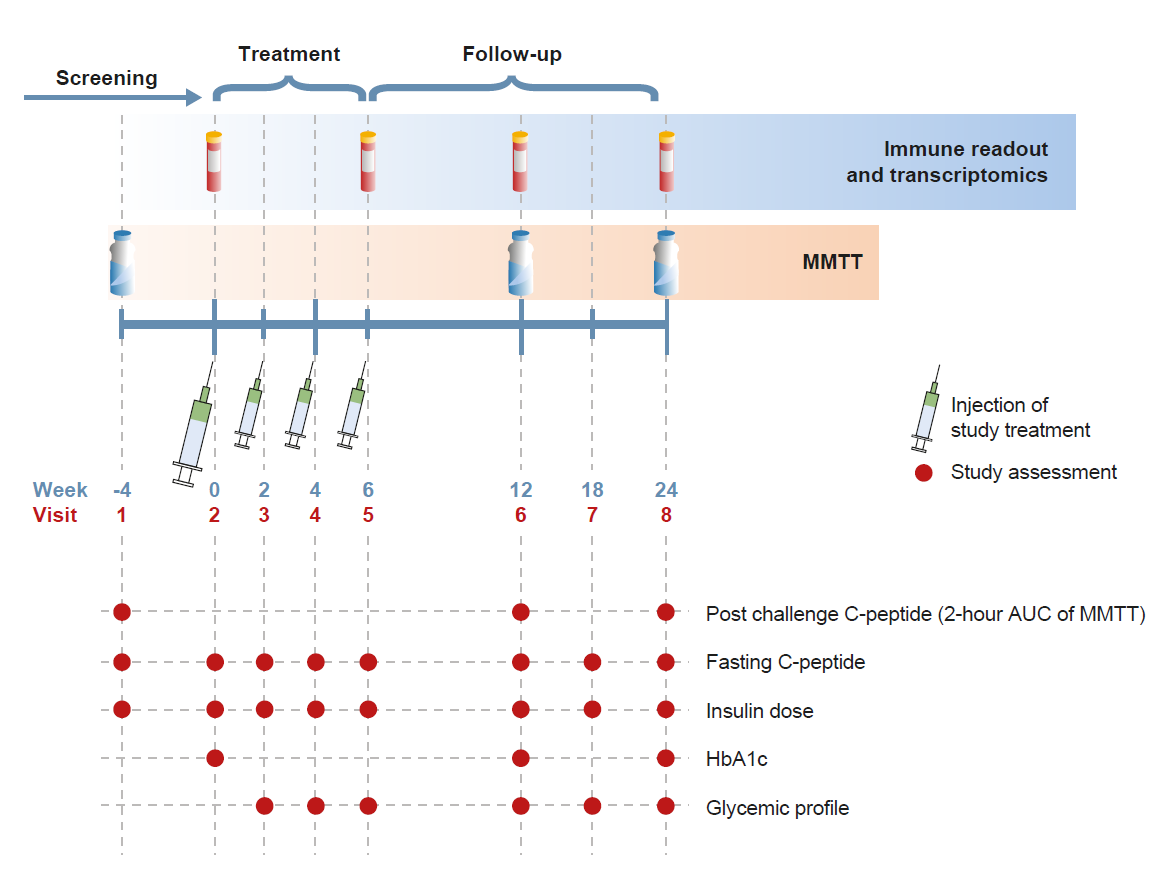


**Fig. S1.** Study visits and assessments.

AUC, area under the curve; HbA1c, glycated hemoglobin; MMTT, Mixed Meal Tolerance Test.


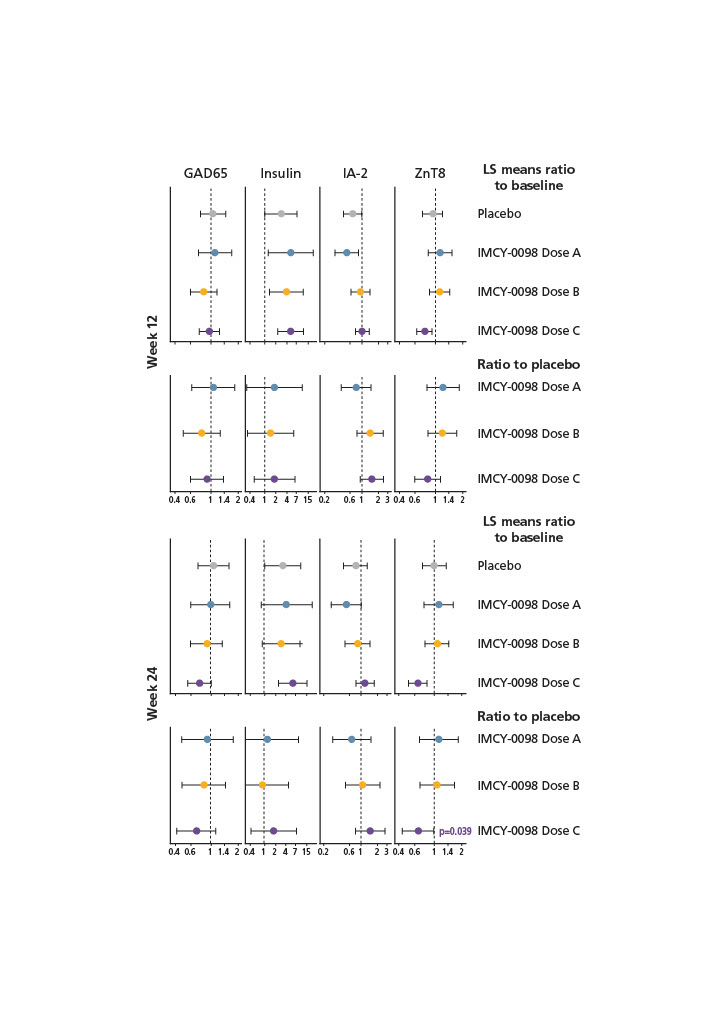


**Fig. S2.** Evolution of autoantibody levels over time – Intent-to-treat population.

Error bars represent 95% confidence intervals. For each autoantibody ratio between Week 12/24 and baseline, and between treatment and placebo, p>0.05 unless indicated otherwise.

Note that an increase in autoantibodies against insulin is in line with previous observations in individuals receiving exogenous insulin.

Dose A: 50 μg at Week 0 followed by 3x25 μg; Dose B: 150 μg at Week 0 followed by 3x75 μg; Dose C: 450 μg at Week 0 followed by 3x225 μg.

GAD65, glutamate decarboxylase; IA-2, insulinoma-associated anigen-2; LS, least square; ZnT8, zinc transporter 8.

**Supplementary Tables:**

**Table S1**. Summary of treatment-emergent adverse events reported to Week 24 – Safety analysis set

| **AEs, n (%)** | **Placebo (n=10)** | **IMCY-0098 Dose A (n=6)** | **IMCY-0098 Dose B (n=9)** | **IMCY-0098 Dose C (n=16)** | **Total (N=41)** |
| --- | --- | --- | --- | --- | --- |
| **Solicited AEs** | | | | | |
| Subjects with >1 solicited AE | 5 (50.0) | 3 (50.0) | 5 (55.6) | 8 (50.0) | 21 (51.2) |
| AEs by grade |  |  |  |  |  |
| Grade 1 | 4 (40.0) | 3 (50.0) | 2 (22.2) | 1 (6.3) | 10 (24.4) |
| Grade 2 | 1 (10.0) | 0 | 3 (33.3) | 6 (37.5) | 10 (24.4) |
| Grade 3 | 0 | 0 | 0 | 0 | 0 |
| Grade 4 | 0 | 0 | 0 | 1* (6.3) | 1* (2.4) |
| Fatigue | 4 (40.0) | 1 (16.7) | 5 (55.6) | 5 (31.3) | 15 (36.6) |
| Malaise | 0 | 1 (16.7) | 2 (22.2) | 2 (12.5) | 5 (12.2) |
| Headache | 0 | 2 (33.3) | 4 (44.4) | 5 (31.3) | 11 (26.8) |
| Myalgia | 1 (10.0) | 0 | 2 (22.2) | 0 | 3 (7.3) |
| **Unsolicited AEs** | | | | | |
| Subjects with >1 solicited AE | 8 (80.0) | 6 (100) | 9 (100) | 16 (100) | 39 (95.1) |
| AEs by grade |  |  |  |  |  |
| Grade 1 | 5 (50.0) | 5 (83.3) | 5 (55.6) | 6 (37.5) | 21 (51.2) |
| Grade 2 | 2 (20.0) | 1 (16.7) | 4 (44.4) | 10 (62.5) | 17 (41.5) |
| Grade 3 | 1 (10.0) | 0 | 0 | 0 | 1 (2.4) |
| Grade 4 | 0 | 0 | 0 | 0 | 0 |
| Nasopharyngitis | 3 (30.0) | 3 (50.0) | 4 (44.4) | 3 (18.8) | 13 (31.7) |
| Upper respiratory tract infection | 0 | 1 (16.7) | 1 (11.1) | 3 (18.8) | 5 (12.2) |
| Rhinitis | 1 (10.0) | 0 | 1 (11.1) | 2 (12.5) | 4 (9.8) |
| Gastroenteritis | 0 | 0 | 0 | 2 (12.5) | 2 (4.9) |
| Tonsilitis | 1 (10.0) | 0 | 1 (11.1) | 0 | 2 (4.9) |
| Viral upper respiratory tract infection | 0 | 0 | 0 | 2 (12.5) | 2 (4.9) |
| Cough | 0 | 0 | 3 (33.3) | 3 (18.8) | 6 (14.6) |
| Oropharyngeal pain | 0 | 0 | 0 | 5 (31.3) | 5 (12.2) |
| Rhinorrhea | 0 | 0 | 2 (22.2) | 2 (12.5) | 4 (9.8) |
| Limb injury | 1 (10.0) | 0 | 0 | 1 (6.3) | 2 (4.9) |
| Headache | 1 (10.0) | 1 (16.7) | 1 (11.1) | 3 (18.8) | 6 (14.6) |
| Back pain | 1 (10.0) | 1 (16.7) | 0 | 1 (6.3) | 3 (7.3) |
| Pain in extremity | 1 (10.0) | 0 | 1 (11.1) | 0 | 2 (4.9) |
| Hypoglycemia | 0 | 0 | 2 (22.2) | 4 (25.0) | 6 (14.6) |
| Diarrhea | 0 | 1 (16.7) | 2 (22.2) | 0 | 3 (7.3) |
| Influenza-like illness | 0 | 0 | 0 | 2 (12.5) | 2 (4.9) |
| Blood creatinine increased | 0 | 0 | 0 | 2 (12.5) | 2 (4.9) |
| Anxiety | 0 | 0 | 1 (11.1) | 1 (6.3) | 2 (4.9) |
| Dysmenorrhea | 0 | 0 | 1 (11.1) | 1 (6.3) | 2 (4.9) |
| Vertigo | 1 (10.0) | 0 | 0 | 1 (6.3) | 2 (4.9) |
| Seasonal allergy | 0 | 1 (16.7) | 1 (11.1) | 0 | 2 (4.9) |

AEs by preferred term are presented only for those that occurred in >1 patient

*****Adverse event was recorded as Grade 4 but site confirmed the event was Grade 1

**Table S2.** Change in vital signs from Screening to Week 24 – Safety analysis

| **Parameter,  Screening mean, ±SD; [and change at Week 24]** | **Placebo (n=10)** | **IMCY-0098 Dose A (n=6)** | **IMCY-0098 Dose B (n=9)** | **IMCY-0098 Dose C (n=16)** | **Total (N=41)** |
| --- | --- | --- | --- | --- | --- |
| Temperature, °C | 36.13, 0.529  [0.15, 0.438] | 36.25, 0.394  [-0.35, 0.472] | 35.69, 0.582  [0.40, 0.608] | 36.09, 0.564  [-0.05, 0.736] | 36.03, 0.554  [0.05, 0.637] |
| Systolic blood pressure, mmHg | 116.3, 9.08  [4.0, 6.20] | 115.0, 12.12  [-0.3, 9.44] | 113.3, 13.74  [1.6, 9.77] | 120.2, 8.96  [0.9, 7.97] | 117.0, 10.61  [1.6, 8.06] |
| Diastolic blood pressure, mmHg | 69.9, 10.37  [-2.2, 10.21] | 66.0, 8.07  [1.7, 8.57] | 64.0, 8.08  [7.3, 6.98] | 70.0, 10.20  [1.5, 10.03] | 68.1, 9.54  [1.9, 9.54] |
| Heart rate, beats/min | 68.1, 10.62  [2.8, 12.13] | 60.0, 16.57  [-2.5, 16.43] | 61.2, 9.12  [7.1, 6.90] | 60.1, 11.07  [-2.8, 8.93] | 62.3, 11.56  [0.8, 11.08] |
| Weight, kg | 71.7, 9.827  [0.44, 4.376] | 73.50, 7.760  [-1.80, 5.638] | 70.81, 7.001  [0.72, 3.077] | 75.33, 11.336  [-0.11, 4.105] | 73.18, 9.535  [-0.05, 4.134] |

**Table S3**. 2 hour AUC of MMTT: analysis of change from screening to Week 24. Intent-to-treat analysis set.

| **Parameter,  Screening mean, ±SD (baseline or Week 24) or SE (differences)** | **Placebo (n=10)** | **IMCY-0098 Dose A (n=6)** | **IMCY-0098 Dose B (n=9)** | **IMCY-0098 Dose C (n=16)** |
| --- | --- | --- | --- | --- |
| Baseline | 0.69, 0.22 | 0.52, 0.16 | 0.70, 0.29 | 0.61, 0.17 |
| Week 24 | 0.62, 0.21 | 0.37, 0.21 | 0.59, 0.31 | 0.57, 0.21 |
| Change from baseline | -0.07, 0.05 | -0.16, 0.06 | -0.07, 0.05 | -0.04, 0.04 |
| Difference vs placebo |  | -0.09, 0.08  p=0.252 | 0.00, 0.07  p=0.994 | 0.02, 0.06  p=0.691 |

p-values show difference vs placebo

AUC, area under-the-curve; MMTT, mixed meal tolerance test; SE, standard error; SD, standard deviation

**Table S4**. Total daily insulin dose per kg: analysis of change from screening to Week 24. Intent-to-treat analysis set.

| **Parameter,  Screening mean, ±SD (baseline or Week 24) or SE (differences)** | **Placebo (n=10)** | **IMCY-0098 Dose A (n=6)** | **IMCY-0098 Dose B (n=9)** | **IMCY-0098 Dose C (n=16)** |
| --- | --- | --- | --- | --- |
| Baseline | 0.32, 0.09 | 0.35, 0.10 | 0.27, 0.18 | 0.36, 0.16 |
| Week 24 | 0.34, 0.14 | 0.36, 0.17 | 0.36, 0.34 | 0.40, 0.19 |
| Change from baseline | 0.02, 0.05 | 0.01, 0.07 | 0.09, 0.06 | 0.04, 0.04 |
| Difference vs placebo |  | -0.01, 0.09  p=0.897 | 0.07, 0.08  p=0.411 | 0.02, 0.07  p=0.831 |

p-values show difference vs placebo

SE, standard error; SD, standard deviation

**Table S5.** Summary of treatment-emergent adverse events reported during the long-term follow up – Safety analysis set

| **AEs, n (%) [number of events]** | **Placebo (n=7)** | **IMCY-0098 Dose A (n=4)** | **IMCY-0098 Dose B (n=7)** | **IMCY-0098 Dose C (n=12)** | **Total (N=30)** |
| --- | --- | --- | --- | --- | --- |
| All AEs | 2 (28.6) [4] | 2 (50) [4] | 3 (42.90) [6] | 6 (50) [10] | 13 (43.3) [24] |
| AEs by grade |  |  |  |  |  |
| Grade 1 | 2 (28.6) [3] | 2 (50) [4] | 3 (42.9) [4] | 5 (41.7) [9] | 12 (40) [20] |
| Grade 2 | 1 (14.3) [1] | 0 | 1 (14.3) [2] | 1 (8.3) [1] | 3 (10) [4] |
| Grade 3 | 0 | 0 | 0 | 0 | 0 |
| Grade 4 | 0 | 0 | 0 | 0 | 0 |
| Nasopharyngitis | 0 | 0 | 2 (28.6) [2] | 0 | 2 (6.7) [2] |
| Upper respiratory tract infection | 0 | 1 (25) [1] | 0 | 1 (8.3) [1] | 2 (6.7) [2] |
| Hypoglycemia | 0 | 0 | 0 | 2 (16.7) [3] | 2 (6.7) [3] |
| Cough | 0 | 0 | 2 (28.6) [2] | 0 | 2 (6.7) [2] |

AEs by preferred term are presented only for those that occurred in >1 patient

**Table S6.** Change in clinical chemistry parameters from Screening to Week 48 – Safety analysis set

| **Parameter, Screening mean, ±SD; [and change at Week 48]** | **Placebo (n=7)** | **IMCY-0098 Dose A (n=4)** | **IMCY-0098 Dose B (n=7)** | **IMCY-0098 Dose C (n=12)** | **Total (N=30)** |
| --- | --- | --- | --- | --- | --- |
| Alanine Aminotransferase U/L | 24.9, 13.7  [-6.0, 13.6] | 16.8, 5.6  [11.0, N/A] | 17.6, 6.2  [-3.7, 7.1] | 28.3, 20.5  [-9.1, 23.0] | 23.5, 15.3  [-4.5, 18.2] |
| Aspartate Aminotransferase, U/L | 28.4, 12.7  [-11.3, 15.6] | 20.8, 6.8  [4.0, N/A] | 23.1, 5.0  [-3.2, 3.5] | 24.8, 8.6  [-4.9, 10.7] | 24.7, 8.8  [-6.0, 10.7] |
| Lactate Dehydrogenase, U/L | 147.7, 20.4  [-2.8, 15.0] | 137.3, 21.8  [-14.0, N/A] | 150.7, 9.2  [-2.8, 15.5] | 151.8, 28.6  [-3.2, 25.5] | 148.7, 22.1  [-3.4, 20.0] |
| Creatine Kinase, U/L | 391.7, 589.3  [-349.3, 635.4] | 173.3, 118.3  [-76.0, N/A] | 96.4, 33.2  [10.3, 25.6] | 148.1, 128.3  [-8.7, 145.5] | 196.2, 304.0  [-92.6, 341.9] |
| Alkaline Phosphatase, U/L | 66.9, 26.0  [-2.5, 22.0] | 59.5, 10.5  [4.0, N/A] | 69.1, 14.5  [2.0, 10.1] | 61.7, 21.8  [1.9, 13.3] | 64.3, 19.7  [1.0, 14.4] |
| Bilirubin, umol/L | 10.5, 6.8  [-3.8, 2.4] | 7.4, 3.1  [2.2, N/A] | 9.1, 6.5  [-1.0, 4.7] | 12.4, 9.8  [0.4, 10.62] | 10.5, 7.7  [-0.8, 7.8] |
| Creatinine, umol/L | 73.5, 12.9  [2.4, 9.0] | 70.1, 10.3  [-6.2, N/A] | 76.8, 16.1  [-2.48, 11.0] | 73.2, 12.1  [0.9, 9.7] | 73.7, 12.6  [0.1, 9.5] |
| Urea, mmol/L | 4.2, 1.0  [-0.4, 0.8] | 4.6, 1.1  [-3.2, N/A] | 4.9, 2.0  [-0.5, 0.9] | 4.4, 0.9  [0.8, 1.1] | 4.5, 1.2  [0.3, 1.3] |
| Albumin, g/L | 48.1, 3.2  [-2.7, 2.7] | 45.3, 1.3  [-1.0, N/A] | 46.1, 4.2  [-1.7, 2.5] | 47.9, 4.3  [-2.5, 3.3] | 47.2, 3.7  [-2.3, 2.8] |
| Gamma Glutamyl Transferase, U/L | 14.1, 3.7  [-1.3, 4.3] | 16.5, 4.8  [9.0, N/A] | 15.0, 5.0  [-2.5, 3.8] | 13.8, 5.7  [2.3, 11.1] | 14.5, 4.8  [0.6, 8.4] |

**Table S7.** Change in hematology parameters relative to normal range from Screening to Week 48 – Safety analysis set.

| **Parameter, n (%)** |  | **Placebo (n=7)** | **IMCY-0098 Dose A (n=4)** | **IMCY-0098 Dose B (n=7)** | **IMCY-0098 Dose C (n=12)** | **Total (N=30)** |
| --- | --- | --- | --- | --- | --- | --- |
| Erythrocytes (10^12/L) | Normal to Normal | 5 (71.4%) | 1 (25.0%) | 5 (71.4%) | 9 (75.0%) | 20 (66.7%) |
|  | Normal to Abnormal NCS | 1 (14.3%) | 0 | 1 (14.3%) | 3 (25.0%) | 5 (16.7%) |
|  | Missing at any of these visits | 1 (14.3%) | 3 (75.0%) | 1 (14.3%) | 0 | 5 (16.7%) |
| Leukocytes (10^9/L) | Normal to Normal | 6 (85.7%) | 1 (25.0%) | 6 (85.7%) | 11 (91.7%) | 24 (80.0%) |
|  | Normal to Abnormal NCS | 0 | 0 | 0 | 1 (8.3%) | 1 (3.3%) |
|  | Missing at any of these visits | 1 (14.3%) | 3 (75.0%) | 1 (14.3%) | 0 | 5 (16.7%) |
| Platelets (10^9/L) | Normal to Normal | 5 (71.4%) | 1 (25.0%) | 6 (85.7%) | 12 (100.0%) | 24 (80.0%) |
|  | Normal to Abnormal NCS | 1 (14.3%) | 0 | 0 | 0 | 1 (3.3%) |
|  | Missing at any of these visits | 0 | 0 | 0 | 0 | 0 |
| Neutrophils (10^9/L) | Normal to Normal | 6 (85.7%) | 1 (25.0%) | 6 (85.7%) | 10 (83.3%) | 23 (76.7%) |
|  | Normal to Abnormal NCS | 0 | 0 | 0 | 2 (16.7%) | 2 (6.7%) |
|  | Missing at any of these visits | 1 (14.3%) | 3 (75.0%) | 1 (14.3%) | 0 | 5 (16.7%) |
| Lymphocytes (10^9/L) | Normal to Normal | 5 (71.4%) | 1 (25.0%) | 6 (85.7%) | 11 (91.7%) | 23 (76.7%) |
|  | Normal to Abnormal NCS | 1 (14.3%) | 0 | 0 | 1 (8.3%) | 2 (6.7%) |
|  | Missing at any of these visits | 1 (14.3%) | 3 (75.0%) | 1 (14.3%) | 0 | 5 (16.7%) |
| Monocytes (10^9/L) | Normal to Normal | 6 (85.7%) | 1 (25.0%) | 5 (71.4%) | 12 (100.0%) | 24 (80.0%) |
|  | Normal to Abnormal NCS | 0 | 0 | 1 (14.3%) | 0 | 1 (3.3%) |
|  | Missing at any of these visits | 1 (14.3%) | 3 (75.0%) | 1 (14.3%) | 0 | 5 (16.7%) |
| Basophils (10^9/L) | Normal to Normal | 6 (85.7%) | 1 (25.0%) | 6 (85.7%) | 12 (100.0%) | 25 (83.3%) |
|  | Normal to Abnormal NCS | 0 | 0 | 0 | 0 | 0 |
|  | Missing at any of these visits | 1 (14.3%) | 3 (75.0%) | 1 (14.3%) | 0 | 5 (16.7%) |
| Eosinophils (10^9/L) | Normal to Normal | 5 (71.4%) | 1 (25.0%) | 5 (71.4%) | 12 (100.0%) | 23 (76.7%) |
|  | Normal to Abnormal NCS | 1 (14.3%) | 0 | 1 (14.3%) | 0 | 2 (6.7%) |
|  | Missing at any of these visits | 1 (14.3%) | 3 (75.0%) | 1 (14.3%) | 0 | 5 (16.7%) |
| Neutrophils/Leukocytes (%) | Normal to Normal | 5 (71.4%) | 1 (25.0%) | 6 (85.7%) | 12 (100.0%) | 24 (80.0%) |
|  | Normal to Abnormal NCS | 1 (14.3%) | 0 | 0 | 0 | 1 (3.3%) |
|  | Missing at any of these visits | 1 (14.3%) | 3 (75.0%) | 1 (14.3%) | 0 | 5 (16.7%) |
| Monocytes/Leukocytes (%) | Normal to Normal | 6 (85.7%) | 1 (25.0%) | 5 (71.4%) | 12 (100.0%) | 24 (80.0%) |
|  | Normal to Abnormal NCS | 0 | 0 | 1 (14.3%) | 0 | 1 (3.3%) |
|  | Missing at any of these visits | 1 (14.3%) | 3 (75.0%) | 1 (14.3%) | 0 | 5 (16.7%) |
| Lymphocytes/ Leukocytes (%) | Normal to Normal | 5 (71.4%) | 1 (25.0%) | 6 (85.7%) | 12 (100.0%) | 24 (80.0%) |
|  | Normal to Abnormal NCS | 1 (14.3%) | 0 | 0 | 0 | 1 (3.3%) |
|  | Missing at any of these visits | 1 (14.3%) | 3 (75.0%) | 1 (14.3%) | 0 | 5 (16.7%) |
| Basophils/Leukocytes (%) | Normal to Normal | 6 (85.7%) | 1 (25.0%) | 6 (85.7%) | 11 (91.7%) | 24 (80.0%) |
|  | Normal to Abnormal NCS | 0 | 0 | 0 | 1 (8.3%) | 1 (3.3%) |
|  | Missing at any of these visits | 1 (14.3%) | 3 (75.0%) | 1 (14.3%) | 0 | 5 (16.7%) |
| Eosinophiles/Leukocytes (%) | Normal to Normal | 5 (71.4%) | 1 (25.0%) | 6 (85.7%) | 11 (91.7%) | 23 (76.7%) |
|  | Normal to Abnormal NCS | 1 (14.3%) | 0 | 0 | 1 (8.3%) | 2 (6.7%) |
|  | Missing at any of these visits | 1 (14.3%) | 3 (75.0%) | 1 (14.3%) | 0 | 5 (16.7%) |

NCS, not clinically significant

**Table S8.** Change in vital signs from Screening to Week 48 – Safety analysis

| **Parameter,  Screening mean, ±SD; [and change at Week 48]** | **Placebo (n=7)** | **IMCY-0098 Dose A (n=4)** | **IMCY-0098 Dose B (n=7)** | **IMCY-0098 Dose C (n=12)** | **Total (N=30)** |
| --- | --- | --- | --- | --- | --- |
| Temperature, °C | 36.2, 40.51  [-0.12, 0.54] | 36.24, 0.51  [0.30, N/A] | 36.24, 0.51  [0.60, 0.74] | 36.24, 0.51  [0.17, 0.55] | 36.24, 0.51  [0.21, 0.62] |
| Systolic blood pressure, mmHg | 112.1, 6.7  [5.2, 8.3] | 118.0, 12.9  [-3.0, N/A] | 115.7, 12.5  [1.2, 3.3] | 119.3, 7.4  [-2.0, 10.0] | 116.6, 9.4  [0.4, 8.5] |
| Diastolic blood pressure, mmHg | 71.9, 11.6  [-1.5, 14.3] | 69.3, 8.3  [-3.0, N/A] | 63.9, 8.7  [1.2, 4.6] | 68.0, 11.0  [-2.4, 10.5] | 68.1, 10.2  [-1.4, 10.0] |
| Heart rate, beats/min | 66.1, 11.5  [0.7, 12.6] | 60.5, 11.7  [-5.0, N/A] | 55.7, 6.4  [6.5, 5.0] | 63.3, 11.3  [-3.8, 9.1] | 1.8, 10.6  [-0.3, 9.8] |
| Weight, kg | 72.41, 11.52  [-0.05, 4.46] | 70.45, 5.84  [4.00, N/A] | 68.41, 5.79  [2.81, 3.11] | 77.74, 11.00  [-0.79, 5.77] | 73.35, 9.95  [0.44, 4.92] |

**List of participating sites:**

| **Institution** | **City** | **Country** |
| --- | --- | --- |
| UZ BRUSSEL | Brussels | Belgium |
| Universitair Ziekenhuis Gent | Gent | Belgium |
| Hôpital Erasme | Brussels | Belgium |
| Bispebjerg and Frederiksberg Hospital | Copenhagen | Denmark |
| Hôpital Lariboisiere AP-HP | Paris | France |
| CHU de Nantes - HGRL | Nantes | France |
| Hopital Cochin | Paris | France |
| Uniklinikum Freiburg | Freiburg I. Breisgau | Germany |
| Helmholtz Zentrum München | München | Germany |
| Universitaetsklinikum Giessen und Marburg GmbH | Gießen | Germany |
| Clinical Study Center for Metabolic Vascular Medicine | Dresden | Germany |
| Barts Health NHS Trust, of Royal London Hospital | London | United Kingdom |
| Cardiff and Vale University Health Board | Cardiff | United Kingdom |
| Guys and St. Thomas NHS Trust Diabetes Day Centre - Guys Hospital | London | United Kingdom |
| Oxford University Hospitals NHS Foundation Trust | Oxford | United Kingdom |
| Royal Devon and Exeter NHS Trust | Exeter | United Kingdom |
| Cambridge University Hospitals NHS Foundation Trust | Cambridge | United Kingdom |
| Royal Victoria Infirmary Newcastle | Newcastle upon Tyne | United Kingdom |
| The Hospital of Lithuanian University of  Health Sciences Kauno klinikos | Kaunas | Lithuania |
| Vilnius University Hospital Santaros Klinikos | Vilnius | Lithuania |
| Klaipeda University Hospital | Klaipeda | Lithuania |
| Clinical Trial Center, CTC, Gothia Forum | Goteborg | Sweden |
| ProbarE Lund | Lund | Sweden |
| ProbarE Stockholm | Stockholm | Sweden |
